# Experimental dissolution of fossil bone under variable pH conditions

**DOI:** 10.1371/journal.pone.0274084

**Published:** 2022-10-13

**Authors:** Colleen A. Sullivan, Sarah W. Keenan

**Affiliations:** Department of Geology and Geological Engineering, South Dakota School of Mines and Technology, Rapid City, South Dakota, United States of America; University of Kurdistan Hewlêr, Kurdistan Region, IRAQ

## Abstract

Fossils exposed at the surface are an integral component of the paleontologic record and provide an archive of past life. However, it is widely known that fossils are not stable indefinitely upon exposure to surface conditions such as physical, chemical, and biological processes, and this last phase of taphonomy is poorly understood. Studies regarding the longevity of fossils subject to weathering, such as acidic precipitation, are absent in the literature. The goal of this study was to experimentally determine vertebrate fossil dissolution rates under variable pH conditions in a controlled laboratory setting. It was hypothesized that fossils would dissolve within acidic solutions and do so at an increasing rate when exposed to increasingly acidic solutions. The experiments were conducted on three fossil vertebrae in triplicate in closed reaction vessels at pH 4, 5, and 6. The fossils were completely submerged for 21 days in a tap water solution with the pH adjusted using 0.1N hydrochloric acid (HCl). Fossil dissolution was quantified by changes to: (1) fossil mass; (2) elemental chemistry of water and fossils with inductively coupled plasma mass spectrometry (ICP-MS); (3) fossil mineralogy with X-ray diffraction (XRD); and (4) histologic structures with thin section analyses. All fossils exhibited mass loss, which increased with decreasing pH conditions, and was greatest under pH 4 (477 to 803 mg loss). The elemental analyses with ICP-MS indicated an increase of both calcium (maximum increase of 315 ppm) and phosphorus (increase of 18 ppm) in aqueous solutions with increasing pH and a loss of those same elements from the fossils (maximum loss of 10 ppm Ca and 6 ppm P). XRD revealed loss of gypsum in all post-dissolution samples. Taken together, the results of ICP-MS and XRD suggest dissolution of the primary mineral phases, including hydroxylapatite, and secondary phases, particularly calcite and gypsum, resulting in an estimated mass loss at pH 4 of 23 to 28 mg per day. Thin section analysis showed degradation of both cortical and trabecular bone in all post-dissolution images, demonstrating physical changes to the fossils as a result of water-rock interactions. These findings constitute the first quantitative analysis of fossil dissolution rates and provide insights into this last stage of taphonomy, addressing a largely understudied potential bias in the vertebrate fossil record.

## Introduction

Fossils represent a physical archive of past life. The processes that lead to the preservation of organisms as fossils, including their bones, culminate in the exposure of material at the surface through weathering, erosion, and stratigraphic uplift [1, 2]. Once exposed, fossils are subject to a suite of taphonomic processes that can damage specimens, such as weathering and abrasion [2], plant growth [3], animal scavenging [4], and acidic precipitation [5]. However, there are still significant questions remaining about the longevity of fossil bones exposed to physical, biological, and chemical weathering, and what types of biases these processes may impart on our vertebrate fossil record.

The interactions between water and rocks or minerals represent one of the main controls on weathering and erosion and drive global biogeochemical cycles [6]. Many rocks and minerals, particularly carbonates and phosphates, are sensitive to variable pH conditions, often undergoing enhanced rates of dissolution with decreasing pH conditions [7]. In environmental research, precipitation specifically is considered acidic if the pH is below 5.6 and alkaline if the pH is above 5.6 [8]. Precipitation pH data for the United States from 1978–2017 indicates that only 12.10% of volume weighted mean (VWM) precipitation values were alkaline, while the remaining 87.90% were acidic [8]. Additionally, a case study examining the pH of snow in the Colorado Rockies of North America revealed nearly an order of magnitude decrease in pH from 5.43 to 4.63 during 1975–1977 [9]. The dominant types of acids found in rainwater include carbonic, sulfuric, and nitric and are controlled by regional atmospheric composition [35]. These persistent acidic pH conditions are potentially of concern for the longevity of fossils exposed at the surface across much of the United States. Due to the abundance of carbonate and phosphate mineral phases comprising fossil bone, and in light of the acidic precipitation found throughout the United States, it is necessary to quantify the rates at which fossils dissolve under variable pH conditions in controlled laboratory settings.

The mineralogical composition of vertebrate fossils in general consists of inorganic materials including phosphate minerals, predominately carbonate-rich hydroxylapatite, with lesser amounts of carbonates, including calcite [10]. Other phases such as pyrite, marcasite, hematite, sphalerite, barite, quartz, siderite, and/or gypsum are typically present as secondary minerals incorporated into void spaces during fossilization [11]. Modern bone consists of 70 weight percent (wt. %) phosphatic minerals, with 60 wt. % comprised of hydroxylapatite [10]. During the fossilization process, hydroxylapatite can recrystallize to a more stable fluorapatite or carbonated fluorapatite phase depending on diagenetic conditions [10, 12].

A dominant environmental factor controlling dissolution or precipitation of phosphate minerals, including phases like apatite, is the pH of the aqueous solutions they are in contact with [13, 14]. When the pH of a solution decreases, the saturation index (SI) of apatite minerals including hydroxylapatite, fluorapatite, and carbonated fluorapatite, may decrease and solubility may increase [13–17]. When the SI is 0 +/- 0.2, the solution is predicted to be in equilibrium; if the SI is negative then it is possible for mineral phases to dissolve; and if the SI is positive then it is possible for the mineral phases to precipitate [14]. Based on prior studies, solutions in contact with hydroxylapatite and a pH below ~7.75, or fluorapatite and a pH below ~7.25, could exhibit dissolution in natural aqueous systems [13]. SI values did not become negative for solutions with carbonated fluorapatite at the pH values studied by Keenan and Engel [13] but were approaching an equilibrium state at pH ~6.25, suggesting lower pH values could enable dissolution of apatite minerals under moderately acidic aqueous geochemical conditions.

The kinetics of hydroxylapatite, fluorapatite, and carbonated fluorapatite dissolution have been examined by several workers at standard temperature conditions (at 25°C). Oliva et al. [14] found that hydroxylapatite dissolution rates decrease with increasing pH in the domain 2.22 ≤ pH ≤ 7.14. They also observed the dissolution rate was relatively constant from pH 2.2–4.5, but once pH >4.5, the dissolution rate started decreasing [14]. This study utilized hydrochloric acid (HCl) in order to buffer the solution to the correct pH. From dissolution experiments by Chaïrat et al. [15], it was found that measured fluorapatite dissolution rates decrease with increasing pH in the domain 3 ≤ pH ≤ 7. This study also used HCl to buffer solution to the correct pH. Harouiya et al. [16] investigated fluorapatite dissolution from pH 1–6 and temperatures 5, 25, and 50°C. While not explicitly stated, Harouiya et al. [16] indicated their findings are consistent with previous studies, including Chaïrat et al. [15] and Guidry and Mackenzie [17], with dissolution rates decreasing with increasing pH (3 ≤ pH ≤ 6) in solutions buffered with HCl. Guidry and Mackenzie [17] also observed that carbonated fluorapatite dissolution rates decrease with increasing pH in the domain 4 ≤ pH ≤ 7 with HCl-buffered solutions. General trends observed in each of these studies include: 1) the use of an HCl buffer to adjust solution pH; 2) the pH range tested was low pH (~ 2) to circumneutral pH (~7); and 3) all three apatite phases previously examined exhibited a negative correlation between dissolution rate and increasing pH.

Importantly, thermodynamic models and experimentally-derived kinetics of the stabilities of apatite phases under low-temperature and circumneutral pH conditions are based on single phase systems, which is not representative of fossil bone [11, 14–17]. The heterogeneous mineralogy of fossils, often consisting of multiple apatite chemistries with secondary mineral phases, leaves the response of fossils to variable pH conditions unknown. Because pH influences hydroxylapatite stability in environmental systems based on thermodynamic and kinetic predictions, and the mineralogy of fossil bones is often complex, there is a need to conduct laboratory-based experiments to determine and to quantify the effects of variable pH on fossil bone stability. Laboratory-based experiments are necessary to control for field variables such as temperature fluctuations, wildlife and plant interactions, wind-based erosion, etc. [2–5].

The goal of this study is to determine dissolution rates of fossil bone, utilizing Mosasauridae specimens from the Pierre Shale Formation, when exposed to acidic aqueous solutions of varying pH under controlled laboratory conditions. This study examines the effects of acidic solutions on fossils towards the end of their taphonomic history, after exposure at the surface to acidic precipitation. The end of a fossil bone’s taphonomic history would be complete degradation and return of ions back into modern ecosystems and nutrient pools. It is hypothesized that Mosasauridae fossils will dissolve when exposed to acidic aqueous solutions, that the rate of dissolution will increase as the pH of the solution decreases, and that dissolution will occur on a short, 3-week time scale. The results of this study have significant implications for understanding fossil long-term stability at the surface in the field where material is exposed to precipitation. Additionally, the results of this study provide, to our knowledge, the first quantitative assessment of fossil bone dissolution rates, which is an understudied and potentially significant bias in our vertebrate fossil record. These results provide a framework for conducting future research to further quantify dissolution rates of fossils with different diagenetic histories, different chemical compositions, and exposure to variable aqueous solutions (e.g., increased salinity).

## Methods and materials

Fossils for this study were selected based on: (1) the abundance of material available for research; (2) the ability to conduct destructive analyses; and (3) the relevance to conservation efforts in the Black Hills Region of South Dakota. This study utilized four fossil vertebrae from a Mosasauridae specimen, the family-level classification of large marine reptiles commonly preserved in the late Cretaceous Pierre Shale Formation (~100–65 million years ago) [18]. Vertebrae are commonly preserved in the fossil record, particularly for Pierre Shale specimens [19]. The Mosasauridae fossils and permission to conduct destructive analyses were provided by the South Dakota Mines Museum of Geology (MoG) directors and curators. Four vertebrae (collection number SDSM 010876) were selected since they were fragmentary, but still recognizable as Mosasauridae. One fossil was used for preliminary results to test how quickly pH changes in an unbuffered solution to select an appropriate time over which to conduct the dissolution experiments. Three fossils (subsequently referred to as F1, F2, F3) were used for each of the three pH conditions tested (pH 4, 5, 6). All physical materials (i.e., thin section slides, remaining powders or bone fragments) are accessioned at the SDSM under the original collection number (SDSM 010876).

### Sample preparation

**Fig 1:**
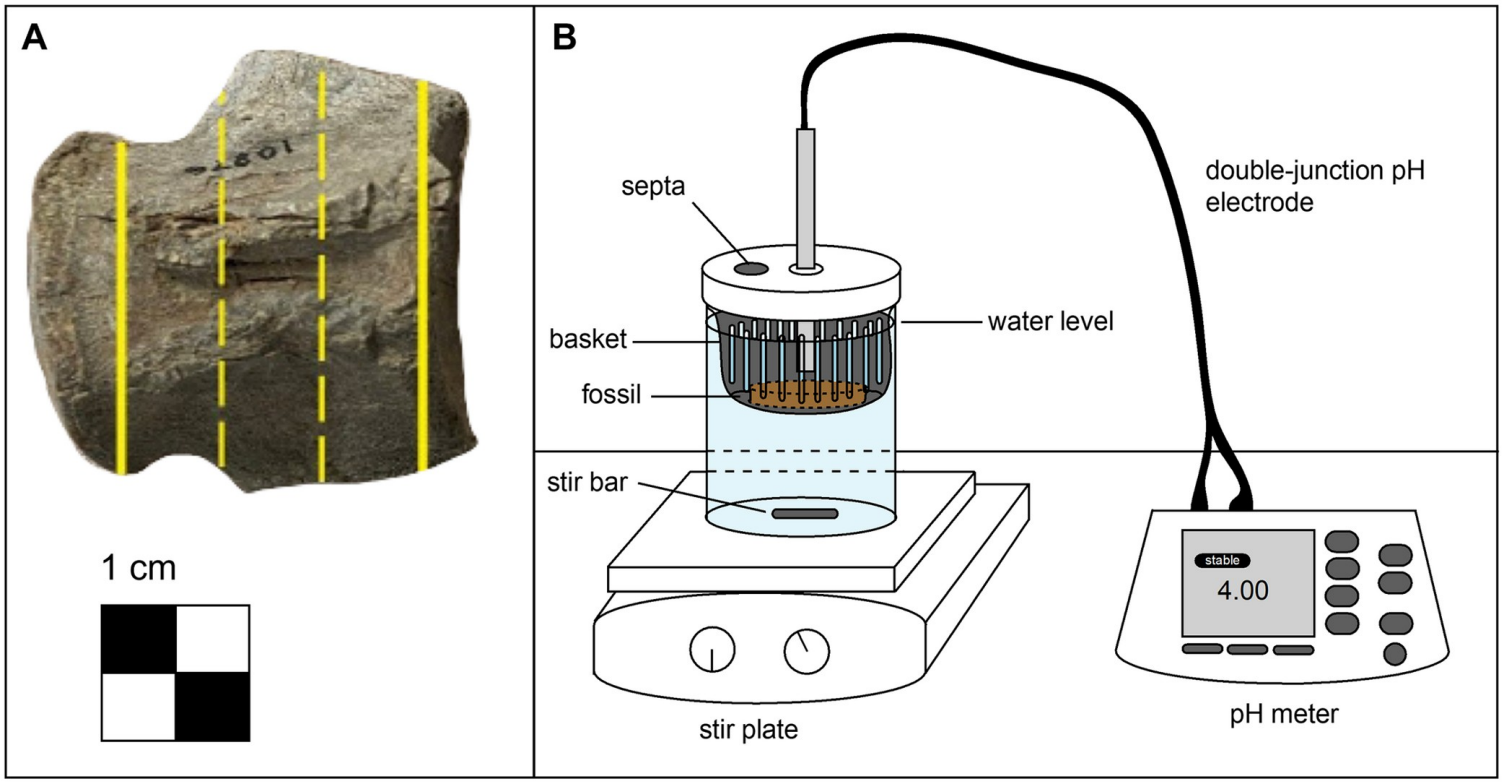
Fossil sectioning and experimental set up. (A) Depiction of the fossil sectioning process. The solid lines indicate cuts made to remove the end caps and dashed lines indicate cuts made to section the fossil into thirds. (B) Schematic of the dissolution experiment closed reaction vessel.

The Mosasauridae specimens used in this study did not have neural or hemal arches preserved, although some specimens retained remnants of the processes observed as small protrusions on the dorsal and ventral surfaces (Fig 1A). The vertebrae used in this study were procoelous, and the anterior and posterior ends of the fossil specimens were removed to maintain a more uniform shape and uniform exposure of cortical and trabecular bone surfaces [20]. Each vertebra was cut into thirds, which allowed the experiment to be conducted in triplicate. The specimens were cut with a diamond blade rock saw (South Dakota Mines, Department of Geology and Geological Engineering). Resulting cross-sectional discs were approximately 1 cm thick with a diameter of 4 cm.

Once cut, the fossils were sterilized in solutions of increasing ethanol concentrations (10%, 50%, 75%, and 95%). The fossils were submerged in each solution for 20 minutes. Following sterilization, the fossil specimens were dried of excess fluid in a fume hood at room temperature for 24 hr. The initial dry mass was measured using an analytical balance (Denver Instrument SI-64) after the fossil completely dried, and samples were stored in sterile Whirl-Pak bags (Nasco) prior to initiating the experiments. The three fossils used in these experiments—F1, F2, F3—were cut into thirds, which allowed each fossil to be used in each pH treatment.

### Dissolution experiments

The experiments were conducted in triplicate using closed systems with continuous stirring. Each experimental system consisted of a ~950 mL (32 oz) mason jar positioned on a stir plate with a stir bar placed at the bottom of the jar, a pH electrode fixed into the lid, a fossil bone suspended and submerged in the water column, and 800 mL of water, described below (Fig 1B). Once the system was completely set up, there was ~2.5 cm of headspace in each jar. The equipment used in the experiments—mason jar, lid, plastic basket, and septa—were sterilized prior to use with a dilute bleach solution. Two, 1.27 cm (0.5 in) diameter holes were cut into the lid of the mason jars prior to sterilization. One hole allowed for the pH electrode to be inserted into the jar and was sealed in place with silicone epoxy (General Electric (GE), manufactured by Momentive Performance Materials, Inc.) to create a closed system. The second hole was sealed with chlorobutyl septa to permit the addition of buffers as needed via sterile syringe and 16-gauge needle to maintain the desired pH. Fossils were placed in a plastic basket originally designed for hydroponics (Growneer, model number NC003-25), suspending the fossils above the base of the jar completely within the water column and out of direct contact with the stir bar. The basket was designed to fit the diameter of the mason jar, while still allowing for the lid to be securely sealed.

These experiments utilized three pH electrodes (Fisher Scientific, Catalog No. 13-620-631), which allowed for experiments to be conducted in triplicate simultaneously. The electrodes were cleaned before each experiment with pH Electrode Cleaning Solution (Oakton, Catalog No. 13-300-179) and deionized (DI) water. During cleaning between experiments, the electrodes were inspected to ensure no precipitate buildup was present on the bulb and that the internal solution (KCl) was full. Finally, the electrodes were calibrated before being sealed into the lid of each mason jar.

The solvent used in the experiments consisted of Rapid City municipal tap water with 0.1N HCl (CAS 7647-01-0, trace metal grade hydrochloric acid, Fisher Chemical) added to each jar to reach the desired pH. Initial water pH was 7.98. Experiments were conducted in triplicate on three separate stir plates. Fossils were exposed to aqueous solutions of pH 4, 5, and 6 for three weeks and monitored every two hours from 08:00 to 18:00 daily. At each two-hour interval, solution pH (starting and ending), quantity of HCl added, and time were recorded (S1 Appendix). Dilute 0.1N HCl was then injected into the closed system via a sterile syringe with a 16-gauge needle through the septa as needed to adjust to the starting pH. The observed maximum threshold for pH variation was: +/- 1.72 for pH 4, +/- 0.90 for pH 5, and +/- 0.56 for pH 6. Maximum variations were observed when the system buffered overnight (14 hr). Any deviations, such as overshooting the desired pH by adding too much HCl or early/late acid treatment due to weather, or additional observations, such as precipitate formation, were also recorded. The temperature was a constant 20°C in a temperature-controlled laboratory. The light exposure was variable, with artificial laboratory lights on while present in the lab and off when no personnel were present. The solution was stirred continuously at approximately 60 rpm for three weeks.

Samples of the initial solution chemistries were collected for all treatments prior to introducing the fossils and sealing the jars (pre-dissolution). At each sampling time, 9 mL of aqueous solutions were collected from each mason jar using pipettes, transferred to a sterile 15 mL Falcon tube, and refrigerated until ICP-MS analyses could be conducted, typically within 1 week. Following initial solution sample collection, fossils were placed into the jar, the lid closed, and a pH electrode sealed into place. Once three weeks elapsed, post-dissolution water samples were collected in the same manner (9 mL, as described above), and the fossils were removed and dried in a fume hood at room temperature for 24 hr. The post-dissolution mass of each bone was measured after drying. Each pH trial of the dissolution experiment was conducted in triplicate with results of each recorded to permit statistical analyses.

Controls were also used for each acid treatment that consisted of a mason jar, lid, plastic basket, tap water, and HCl added to adjust the solution to the correct pH. The controls were allowed to sit for 21 days, the same duration as the treatments with fossils. The controls were utilized to determine potential contributions to the water chemistry from the plastic basket and glass. The control was sampled in the same manner as the aqueous solution for pre-dissolution and post-dissolution samples.

### ICP-MS analyses

Inductively coupled plasma mass spectrometry (ICP-MS) analyses were performed using an Agilent 7900 ICP-MS at the Engineering and Mining Experiment Station (EMES) at South Dakota Mines to determine the elemental composition of the pre- and post-dissolution fossil specimen and water chemistry from the dissolution experiments. To conduct analyses on the fossil samples, approximately 100 mg of sample was powdered to a uniform consistency with a Dremel drill and digested with 5 mL of concentrated (70%) trace metal grade nitric acid (Fisher Chemical, Catalog No. A509P500) overnight on a hot block (50°C) then diluted to a final acid concentration of 2%. The aqueous solution samples were acidified to 2% nitric acid to ensure analytes of interest remain in solution until analyses are conducted. Selected elements were quantified (in parts per million, ppm) including calcium, phosphorus, sodium, potassium, aluminum, magnesium, iron, manganese, barium, and strontium. Quantification was performed using multipoint calibration curve generated using standard solutions prepared by serial dilution of a multielement stock standard solution (SCP Science, Catalog No. 140-130-301). Calibration accuracy was confirmed utilizing a second multielement stock standard solution from a different supplier (High Purity Standards, Catalog No. ICP-MSCS-PE3-A). Three mineral samples were also powdered and digested including two apatite specimens (Eisco, ESNG0065 and Ward’s Scientific, 470226–354) and one calcite (Ward’s research grade, 49–5860). If necessary, samples were diluted prior to analysis to ensure values fell within the calibration range, particularly for calcium. ICP-MS samples were analyzed in triplicate in helium collision mode. Average concentrations and their relative standard deviation are reported for the triplicate analyses of each sample (S2 Appendix).

### XRD analyses

X-ray diffraction (XRD) analyses were conducted using a Rigaku Ultima-Plus X-Ray Diffractometer at the EMES at South Dakota Mines. To prepare samples for analyses, approximately 500 mg of the pre- and post-dissolution bones (cortical and trabecular combined) were powdered with a Dremel drill to a uniform consistency: one pre-dissolution sample of each fossil (n = 3) and all nine post-dissolution fossil samples (n = 3 per treatment) for a total of twelve XRD analyses. Only one pre-dissolution sample per fossil was analyzed because the initial composition of the samples cut from the same fossil were expected to be uniform. The XRD analyses were completed under 40kV and 45mA. Samples were analyzed on a glass sample holder and spread to be a uniform thickness. The sample holder was then set in the middle of the diffractometer sample tray and scanned from 2° to 90° 2θ with Co Kα radiation.

The XRD diffractograms for each sample analyzed were collected with the Data Collector software. The diffractograms were then analyzed with the software JADE to determine mineral content and crystallite size. This software took known mineralogical diffractograms from internal databases and matched them to the resultant diffractograms from the fossil scans to determine which minerals were present in the sample and the weight percent of those identified minerals. A good fit was determined by ensuring the refinement to error ratio (R/E) was below 3.0. Fossil three (F3) had a barite phase detected as a mineralogical constituent. The barite phase needed to be set to “preferred orientation” in order for the R/E to be below 3.0. JADE was used to calculate crystallite size using the Sherrer equation with crystal defects evaluated with the Full Width at the Half Maximum (FWHM) measurements. Outputs were saved as diffractogram images as well as PDF reports with the mineralogical composition in weight percent (S3 Appendix). Stacked diffractograms were created to compare differences between diffractogram patters of pre-dissolution and post-dissolution samples. Mineralogical composition in weight percent (wt. %) and crystallite size are reported. The XRD data were used to aid interpretations of the elemental compositions and sources of elements determined with ICP-MS.

### Thin section preparation

Thin sections of the fossil vertebrae were created for petrographic analysis to determine mineralogical composition and to evaluate histological structures in pre- and post-dissolution samples. Thin sections of pre-dissolution and post-dissolution fossil specimens were generated by National Petrographic Service, Inc. (Rosenberg, TX). The specimens were cut to size (27 x 46 mm; 30 *μm* thickness) and then embedded in an epoxy resin. Thin section preparation followed standard methods used for rocks.

Petrographic analyses of the thin sections were conducted using an Olympus BX60 microscope at South Dakota Mines with plane polarized light (PPL) and cross polarized light (XPL) to visually assess the minerals present and the degree of histologic structure degradation. The accompanying computer software, Olympus cellSense, was used to photograph the thin sections (S4 Appendix).

### Data analyses

To quantify the dissolution rate, the mass loss for each pH treatment was averaged and divided by 21 to obtain a daily dissolution rate. The amount of fossil dissolution due to each acid solution was quantified by changes to the fossil and the water chemistry. Changes to the fossil were evaluated through mass loss, changes to mineralogical composition via XRD, changes to elemental chemistry via ICP-MS, and changes to histologic structures examined in thin section. The mass loss between each pH trial (n = 3) was tested to determine if significant differences (p ≤ 0.05) were observed using paired T-tests. These tests were completed in RStudio (S5 Appendix) [34]. The ICP-MS data from fossil bones comparing the differences in calcium and phosphorus concentrations (n = 3) were also evaluated with paired T-tests. Graphs were created to visually demonstrate the differences in mineralogical composition of each fossil for each pH tested. Finally, thin sections were examined to visually assess changes to histologic structures between pre- and post-dissolution samples.

Dissolution was also quantified via changes to the water chemistry. The pre- and post-dissolution solution chemistry was quantified with ICP-MS to determine the elemental increases and/or decreases due to interactions between the solutions and fossil bones. Paired T-tests were also conducted to evaluate differences in calcium and phosphorus concentrations for both the aqueous solutions and the fossil. The ICP-MS results aided in determining which minerals dissolved based on the elemental chemistry observed in the solution.

## Results

### Mass loss and dissolution rate

The greatest mass loss, reflecting the difference between the pre-dissolution and post-dissolution dry masses of fossil bones, was 802.90 mg, exhibited by F1 during the pH 4 treatment (Table 1). The specimen that exhibited the least mass loss was F3 during the pH 6 treatment, which displayed a total loss of 284.60 mg over the 21-day experiment. The greatest dissolution rate was 39.78 mg/d exhibited by F1 during the pH 5 treatment, and the lowest dissolution rate was 13.55 mg/d exhibited by F3 during the pH 6 treatment. Mass loss and pH are negatively correlated, where increasing pH resulted in decreased mass loss in all three fossils as hypothesized.

**Table 1:** Pre- and post-dissolution masses, total mass loss, percentage mass loss, and dissolution rate per day for all fossils at each pH tested.

Mass Loss						
pH	Sample	Pre-dissolution Mass (mg)	Post-dissolution Mass (mg)	Total Mass Loss (mg)	Percentage Mass Loss (%)	Dissolution Rate (mg/d)
4	F1	51930.30	51127.40	802.90	1.55	38.23
	F2	31247.60	30575.50	672.10	2.15	32.00
	F3	21272.00	20795.40	476.60	2.24	22.70
5	F1	37612.10	36776.80	835.30	2.22	39.78
	F2	27841.20	27551.90	289.30	1.04	13.78
	F3	29305.10	28783.50	521.60	1.77	24.84
6	F1	45499.60	45040.40	459.20	1.01	21.87
	F2	33570.60	33231.90	338.70	1.01	16.13
	F3	27744.10	27459.50	284.60	1.03	13.55

During the pH 4 and pH 5 acid treatments, precipitate formation was observed on the plastic basket, glass jar, and each of the fossils (F1, F2, and F3). Precipitate formation occurred faster in the systems with larger fossils and was first observed in both the pH 4 and pH 5 treatment by day 13. Precipitate formation was not observed during the pH 6 treatment. The precipitate that formed was not extensive enough to be collected or quantitatively analyzed. Each fossil (F1, F2, and F3) from the pH 4 and pH 5 acid treatments that exhibited precipitate formation also exhibited overall mass loss (Table 1). Fossils exposed to pH 4 treatments exhibited a significant difference in terms of mass loss compared to pH 6 treatments as expected (p = 0.0274) (Table 2).

**Table 2:** Paired T-test results of mass loss.

Results of mass loss paired T-tests	
T-test pairs	p-value
pH 4 and pH 5	0.5441
pH 5 and pH 6	0.2724
pH 4 and pH 6	**0.0274**

### ICP-MS

Fossil dissolution was quantified based on the elemental chemistry of the fossils and aqueous solutions with ICP-MS. To calculate changes to the aqueous solution chemistry, the difference between the final and initial elemental concentrations were determined for each aqueous sample at each pH (Table 3). Elements detected in the control were subtracted from the aqueous samples to account for elemental additions or depletions from the glass or plastic basket. Changes to fossil chemistry were calculated by subtracting pre-dissolution concentrations from post-dissolution concentrations (Table 4). There were significant differences in phosphorus concentrations in the aqueous solutions between pH 5 and pH 6 (paired T-tests; p < 0.0001) and pH 4 and pH 6 (p = 0.0234) (Table 5). No significant differences were observed with respect to calcium across the pH treatments, and no significant changes to calcium or phosphorus were observed in the fossils.

**Table 3:** Changes in water chemistry from pre- to post-dissolution samples.

Change in water chemistry (ppm)											
pH	Sample	P	Ca	Na	Mg	Al	K	Mn	Fe	Sr	Ba
4	F1	10.86	314.11	0.88	1.31	1.70	45.51	1.69	2.07	2.58	0.69
	F2	14.36	251.38	0.85	**-0.67**	1.80	148.25	1.23	1.70	2.09	0.40
	F3	18.74	197.15	0.63	**-1.09**	1.45	89.60	1.19	1.55	1.90	0.34
5	F1	6.62	329.77	**-0.39**	0.96	0.02	58.96	1.50	0.68	2.11	0.30
	F2	7.22	109.74	0.64	0.23	**-0.03**	108.23	0.55	0.16	1.06	0.11
	F3	6.69	202.65	**-0.01**	0.27	0.02	75.49	1.39	0.85	1.72	0.11
6	F1	0.00	273.36	0.50	1.49	**-0.04**	37.21	1.16	0.00	1.73	0.20
	F2	0.03	185.48	0.85	0.81	**-0.04**	180.20	0.73	0.00	1.48	0.15
	F3	0.00	174.07	0.09	**-0.80**	**-0.03**	122.76	1.20	**-0.01**	1.36	0.13

The bold values represent elemental depletions in the post-dissolution water chemistry.

**Table 4:** Changes in fossil chemistry from pre- to post-dissolution samples.

Change in fossil chemistry (ppm)											
pH	Sample	P	Ca	Na	Mg	Al	K	Mn	Fe	Sr	Ba
4	**F1**	**-0.89**	**-9.29**	746.36	1459.22	9478.36	763.18	944.01	15098.75	1933.32	2329.25
	**F2**	**-6.07**	**-9.83**	1325.18	1276.14	10889.98	881.96	970.64	12327.87	2203.96	3640.96
	**F3**	3.25	12.40	1671.53	1223.84	3277.19	336.20	1426.04	8263.85	3193.45	**-307.69**
5	**F1**	**-3.15**	**-3.57**	**-1574.20**	556.13	3803.75	358.16	297.07	4373.55	486.07	412.39
	**F2**	**-1.59**	3.44	1795.86	1642.23	15163.92	1153.82	1243.49	16234.07	3040.20	5065.77
	**F3**	0.73	12.72	1962.50	1400.34	3849.33	421.69	1677.51	9670.80	3846.44	605.66
6	**F1**	**-1.81**	**-2.17**	189.99	1909.23	**-970.80**	10.09	860.03	3520.59	2434.29	2007.74
	**F2**	**-6.17**	9.70	58.80	2524.78	**-4867.49**	113.30	1022.28	5201.06	2185.98	1435.97
	**F3**	**-0.69**	9.24	437.70	1244.57	**-267.54**	129.91	1387.32	7990.36	2657.96	9875.53

The bold values represent depletions in the post-dissolution fossil samples.

**Table 5:** Paired T-test results of changes in phosphorus (P) and calcium (Ca) concentrations for aqueous solutions and fossils.

ICP-MS p-values of paired T-tests					
Aqueous Solution	P	Ca	Fossil	P	Ca
pH 4 vs pH 5	0.07564	0.5122	pH 4 vs pH 5	0.9692	0.2287
pH 5 vs pH 6	**6.89E-04**	0.9459	pH 5 vs pH 6	0.4596	0.6693
pH 4 vs pH 6	**0.0234**	0.07355	pH 4 vs pH 6	0.2924	0.355

### XRD

**Fig 2:**
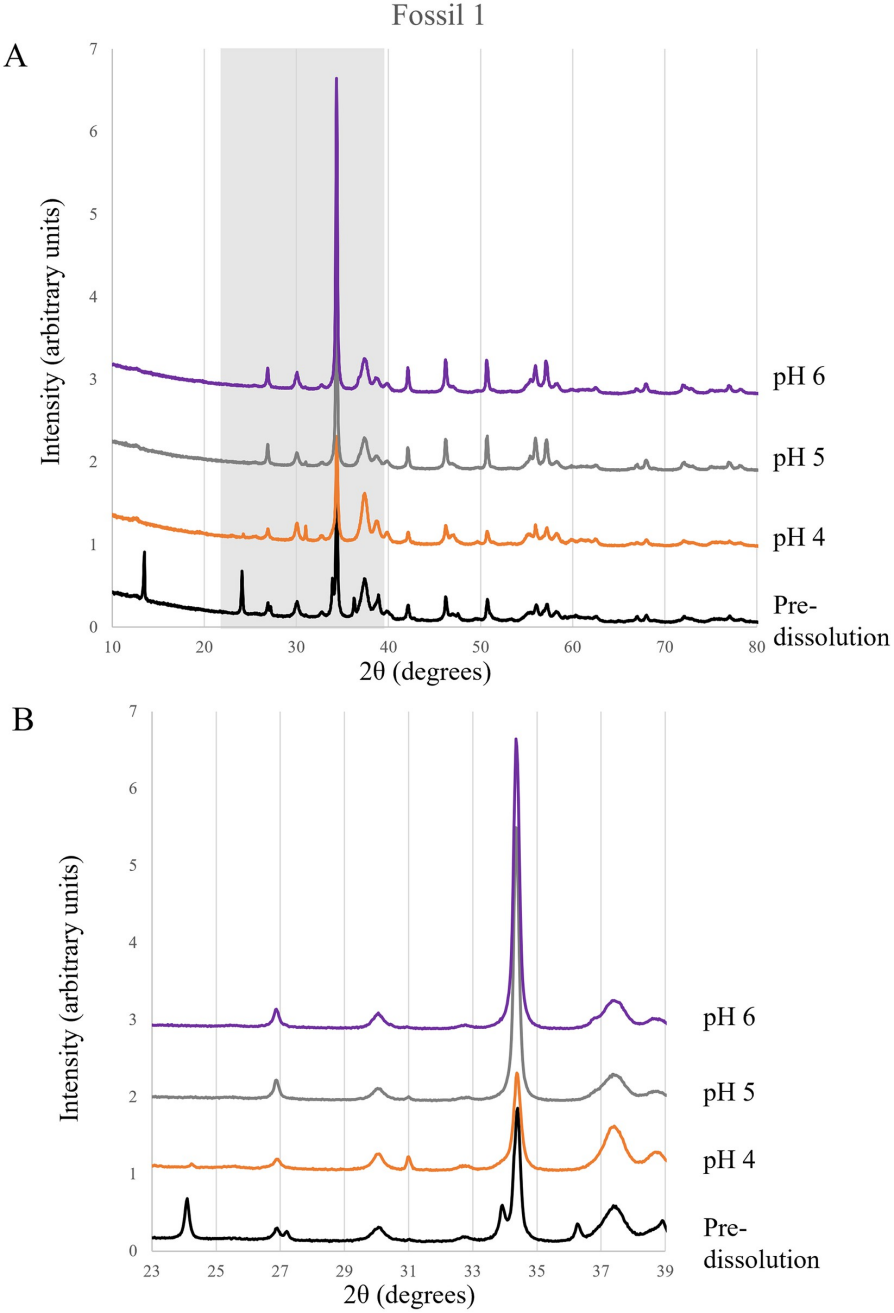
XRD diffractogram patterns for all F1 samples. (A) Stacked diffractograms for all F1 samples. The area highlighted in gray (23–39 degrees) is shown in greater detail in B. (B) Detailed view of the F1 diffractogram pattern from 23–39 degrees.

**Fig 3:**
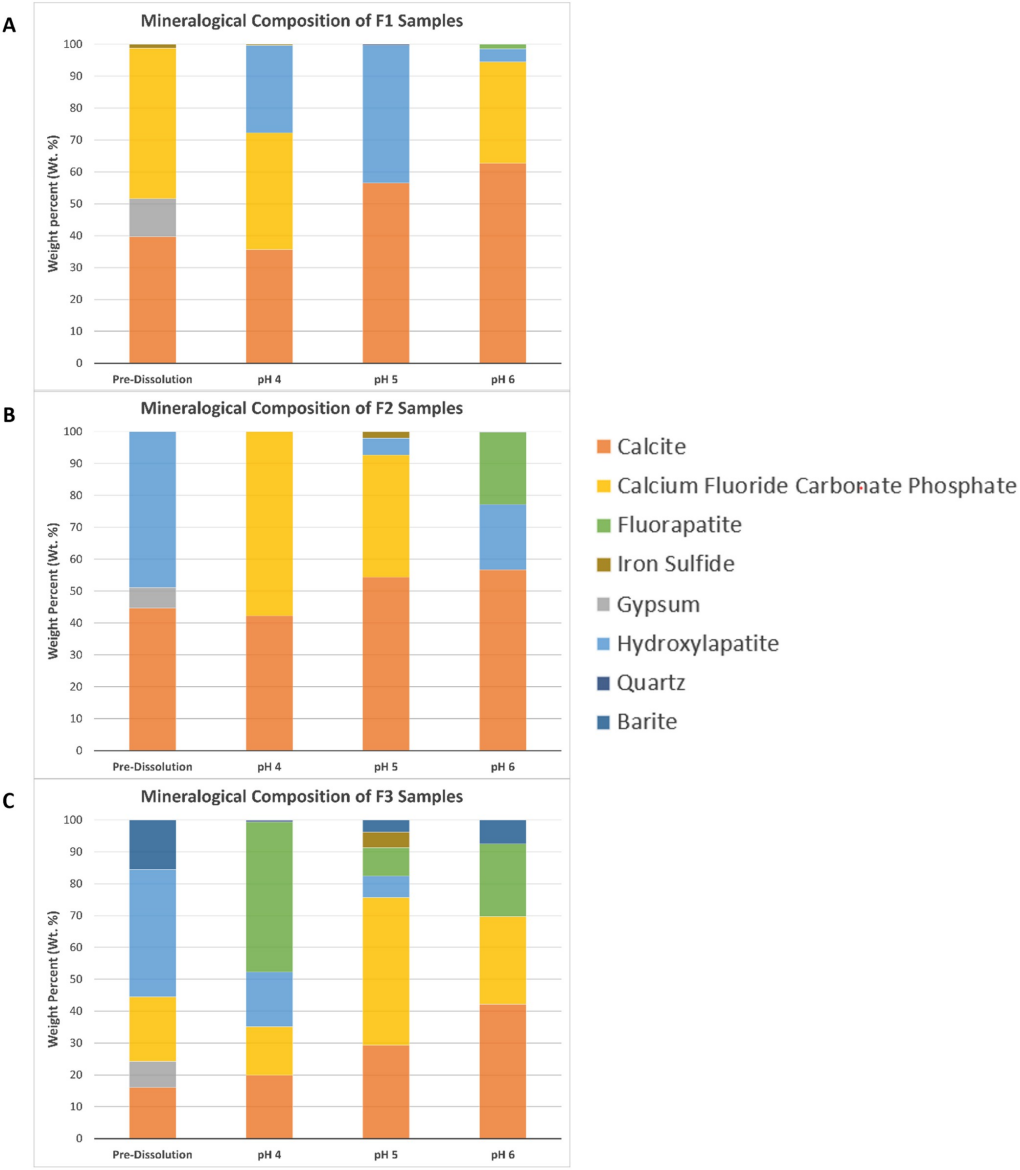
Bar graph of fossil mineralogical composition. Mineralogical composition of pre- and post-dissolution fossils based on XRD analyses. (A) F1 samples; (B) F2 samples; (C) F3 samples.

Fossil mineralogy was determined with XRD and reported as a wt. % (Figs 2 and 3; S3 Appendix). Minerals identified with XRD include calcite, gypsum, three apatite phases (hydroxylapatite, fluorapatite, and carbonated fluorapatite), quartz, iron sulfide, and barite. Iron sulfide minerals include multiple iron and sulfur bearing phases (i.e., marcasite, pyrite, etc.). Because the contributions to the total weight percent of the samples was minimal (4.9 wt. % at the highest detected amount, and 0.34 wt. % the lowest detected amount), iron and sulfur-bearing minerals were considered together.

In each sample, gypsum was detected in the pre-dissolution fossils, ranging from 11.9 wt. % to 6.5 wt. %, but was not identified in any of the post-dissolution samples (Fig 3). The amount of calcite measured in pH 4 samples tended to be less than the pre-dissolution contributions, but in pH 5 and pH 6 samples, calcite wt. % contributions were typically higher than pre-dissolution samples. Barite was only detected in the F3 bone and had the largest wt.% contribution (15.54 wt.%) in the pre-dissolution sample. Barite was absent in the pH 4 sample but was identified in pH 5 (3.8 wt.%) and in pH 6 (7.5 wt.%) fossils. The amount of barite represented in a sample increased between pH 5 and 6. Clear trends were not identified for the iron sulfide phases, quartz, or the three apatite phases.

Analysis of F1 identified only the carbonated fluorapatite phase in the pre-dissolution sample, but varying amounts of hydroxylapatite and carbonated fluorapatite phases in the post-dissolution samples. F1 only had minor amounts of fluorapatite detected in the pH 6 treatment (Fig 3A). F2 only had the hydroxylapatite phase present in the pre-dissolution sample. Varying quantities of fluorapatite and carbonated fluorapatite were present in all post-dissolution samples. Hydroxylapatite in F2 was only identified in pH 4 and pH 5 samples (Fig 3B). F3 had carbonated fluorapatite and hydroxylapatite detected in the pre-dissolution sample. All three apatite phases were present in the pH 4 and pH 5 samples; hydroxylapatite was absent at pH 6, but the fluorapatite and carbonated fluorapatite phases were identified (Fig 3C).

In addition to bulk mineral composition, crystallite size and crystallinity of the three identified apatite phases were evaluated to determine if the pH treatments had an effect on the average crystal size and structural order. The crystallite size and crystallinity was relatively consistent for all pre-dissolution samples. Overall, crystallinity tended to increase with increasing pH, an example being fluorapatite (FAP) in F3 (Table 6). Each fossil (F1, F2, and F3) exhibited exceptions to this trend, for example hydroxylapatite in F3 (Table 6). Additionally, the post-dissolution treatments at each pH tended to have higher crystallinity values than pre-dissolution treatments as observed for carbonated fluorapatite (carbonated FAP) in F2 (Table 6).

**Table 6:** Crystallite size and structural order of detected apatite phases calculated using the Sherrer equation.

Crystallinity (nm)									
Treatment	F1			F2			F3		
	Carbonated FAP	HAP	FAP	Carbonated FAP	HAP	FAP	Carbonated FAP	HAP	FAP
Pre-dissolution	15.6	N/A	N/A	N/A	14.3	N/A	N/A	16.1	N/A
pH 4	23.3	11.3	N/A	16.7	N/A	N/A	24.4	12.6	9.3
pH 5	N/A	17.6	4.7	17.7	28.0	7.9	20.5	6.7	16.1
pH 6	16.9	41.4	9.8	N/A	13.2	34.3	17.1	4.6	23.5

### Thin sections

**Fig 4:**
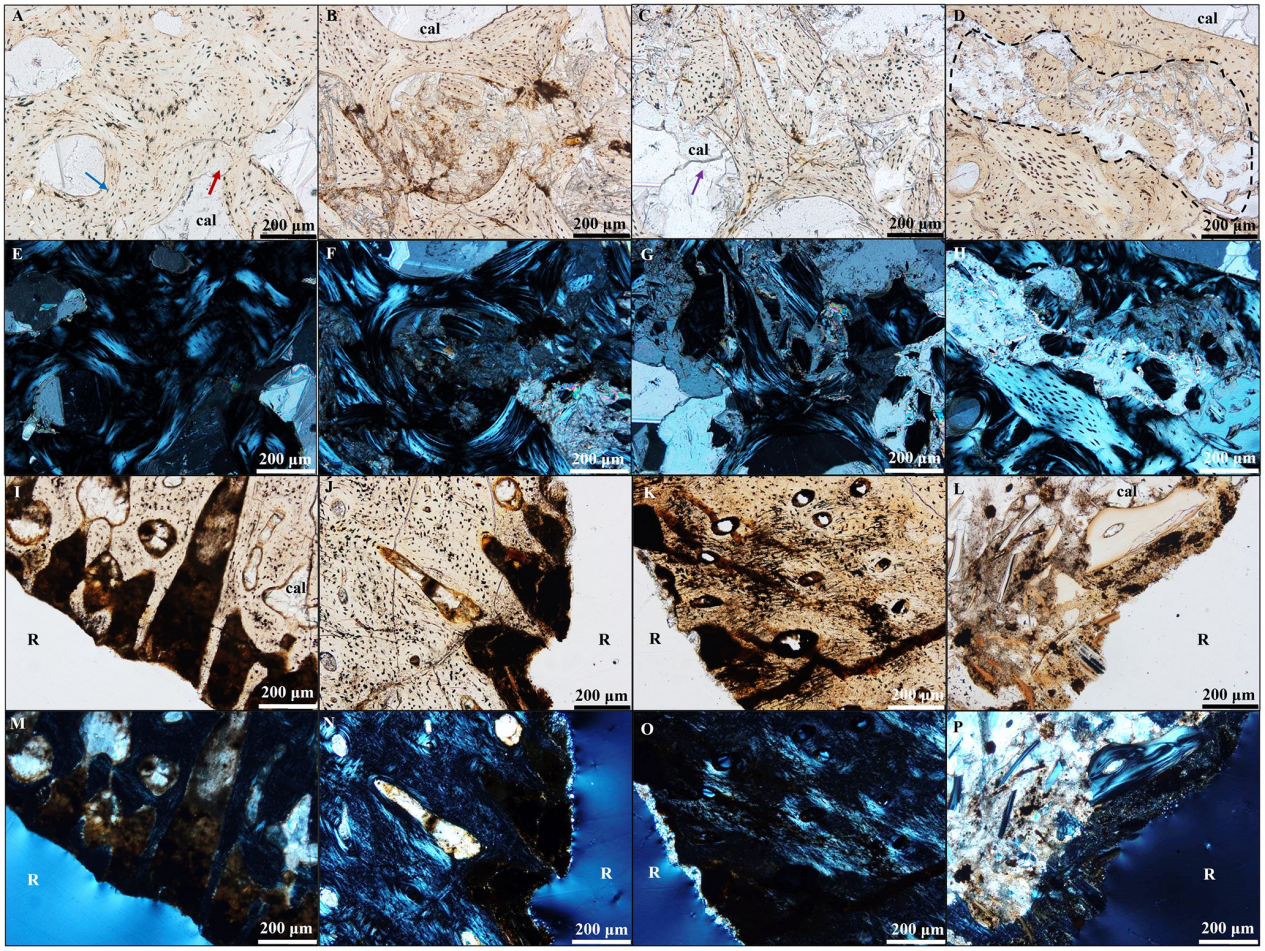
F1 pre-dissolution, pH 6, pH 5, and pH 4 thin sections of cortical and trabecular bone. Degree of dissolution exhibited in trabecular (A-H) and cortical (I-P) bone thin sections of Fossil 1 (F1) viewed under plane polarized light (PPL) and cross polarized light (XPL). (A) Pre-dissolution PPL image of trabecular bone with relatively intact trabeculae (red arrow) and osteocytes (blue arrow). (B) At pH 6, trabecular bone exhibited with the lowest degree of damage to histologic structures, with increased damange under exposure to (C) pH 5. Degradation of secondary mineral infilling (purple arrow) in a marrow space is present as large cracks and breaks in the mineral, observed in the left of the image. (D) F1 dissolved at pH 4 PPL image of trabecular bone. The trabeculae show a high degree of degradation, evident by small (10’s of *μ*m-sized) bone fragments toward the center of the image (area surrounded by a dashed line). XPL images of trabecular bone (E) pre-dissolution, (F) after exposure to pH 6 solution, and (G) dissolved at pH 5, and (H) dissolved at pH 4. (I)Pre-dissolution PPL image of cortical bone with relatively intact histologic structures. (J) F1 dissolved at pH 6 PPL image of cortical bone with the lowest degree of damage to histologic structures. Dissolution can be observed at the margin of the fossil to the right of the image. (K) After exposure to pH 5 solutions, cortical bone exhibited increased degradation. (L) F1 dissolved at pH 4 PPL image of cortical bone with highest degree of degradation to histologic structures. XPL images of cortical bone (M) pre-dissolution, (N) after exposure to pH 6 solution, (O) dissolved at pH 5, and (P) dissolved at pH 4. Abbreviations: R, resin; cal, calcite.

Thin sections were made of pre-dissolution samples (n = 3) as well as post-dissolution samples at each pH to evaluate the mineralogy and location of specific mineral phases in each bone and to view the effects of dissolution at the microstructural level (Fig 4). The three fossils exhibited similar trends as pH increased. The pre-dissolution thin sections of each fossil all appear to have intact histologic structures, including trabeculae and osteocytes. Larger marrow spaces have calcite and gypsum mineral infilling.

Post-dissolution thin sections reveal changes to bone microstructure, including fragmentation of bone and loss of secondary phases, including gypsum and calcite. The pH 4 thin sections show large amounts of degradation exhibited through the breakage of trabeculae into small fragments (mm to micron scale). Some thin sections displayed a preferential pathway of dissolution with the degradation of trabeculae radiating outward from a fracture. The pH 5 thin sections showed less degradation to trabeculae than those in pH 4 thin sections based on the overall size of bone fragments, which were typically larger in pH 5 samples. The pH 6 thin sections usually exhibited the least amount of degradation with trabeculae fragments typically larger and more intact compared to pH 4 thin sections (Fig 4). Degradation was observed in both cortical and trabecular bone but was more pronounced in the trabecular bone.

## Discussion

Exposure of fossil bone to variable pH conditions resulted in substantial dissolution of secondary and primary mineral phases, supporting our hypothesis. The results of mass loss, ICP-MS analyses of bone and solution, and XRD indicate that quantifiable dissolution of secondary minerals and the primary fossil bone apatite occurred after exposure to variable pH conditions. Mass loss, one of the most direct measures of fossil dissolution, increased with decreasing pH (Table 1). This trend was statistically demonstrated through the significant difference between mass loss at pH 4 vs pH 6 (p = 0.0274).

In addition to mass loss, the elemental differences between pre-dissolution and post-dissolution samples strongly point toward dissolution of fossil bone. The ICP-MS analyses identified increases of calcium and phosphorus in solution, with the paired T-tests yielding significant phosphorus values between pH 4 and pH 6 (p = 0.0234) as well as pH 5 and pH 6 (p < 0.0001). Sources of calcium in these fossils include multiple apatite phases, calcite, and gypsum, of which calcite and gypsum are secondary minerals [11–13]. Due to three main sources of calcium in a fossil, including highly soluble gypsum, observed increases of calcium concentrations in aqueous solutions were predicted and consistent with findings from prior studies of single mineral phase systems [14–17, 21, 22]. As the aqueous solutions interacted with the fossils during the experiments, calcium was released into solution. Although the increase of phosphorus in solution was not statistically significant, there was a measurable increase that tended to be higher in lower pH experiments as expected (Tables 2 and 4). In contrast to potential sources of calcium in the experiment, apatite phases were the only phosphorus bearing minerals expected to be present. The most likely source of phosphorus observed to increase in solutions is through the dissolution of one or more of the apatite phases present in fossil bone.

An important consideration when interpreting the ICP-MS data and trends is the exact sources and sinks of measured elements are not conclusive. The expectation was to see increases in phosphorus, calcium, and potassium in the solution, with decreases of those same elements in the fossils, which would indicate dissolution. While some elements exhibited this trend, many elements exhibited other relationships and are further described below (Table 7).

**Table 7:** Interpretation of elemental relationships between fossil and solution samples, based on data presented in Tables 3 and 4^a^^,^^b^^,^^c^.

Scenario	Fossil	Solution	Interpretation	Example
**A**	Decrease	Increase	Dissolution of mineral in fossil bearing element “X”	Phosphorus decreases in the fossil and increase in solution (F1 in pH 4)
**B**	Decrease	Decrease	Precipitate or amorphous phase formation of element “X”	Aluminum decreases in both the fossil and solution (F1, F2, and F3 in pH 6)
**C**	Decrease	No change	Precipitate or amorphous phase formation of element “X”	Phosphorus decreases in the fossil and no change is observed in solution (F1 and F3 in pH 6)
**D**	Increase	Increase	Dissolution of bone exposing deeper portion of the fossil; elements are dissolved into solution and exposed in new areas of the fossil; potential for sampling a site concentrated in element “X” due to heterogeneous fossil composition	Calcium increases in both the fossil and solution (F3 in pH 4)
**E**	Increase	Decrease	Sorption and/or precipitation of element “X” in fossil due to charged sites exposed by dissolution	Magnesium increases in the fossil and decreases in solution (F3 in pH 4)
**F**	Increase	No change	Potential for sampling a site concentrated in element “X” due to heterogeneous fossil composition	Iron increases in the fossil and no change is observed in solution (F1 in pH 6)

^a^Decrease refers to elemental depletions (negative values in Tables 3 and 4)

^b^Increase refers to elemental increases (positive values in Tables 3 and 4)

^c^No change refers to the sample exhibiting no elemental changes in pre-dissolution and post-dissolution samples (zero values in Tables 3 and 4)

The ICP-MS results enable discussion of distinct scenarios that occur after fossil and solution interactions. Scenario A is the expected result of exposure of fossil bone to an aqueous solution where the fossil dissolves, releasing elements into the water. Scenarios B and C, where there are elemental depletions from the fossil and either elemental depletions or no change in the water chemistry, could be explained by the formation of precipitates or amorphous phases in solution.

Scenario D describes when the fossil and solution both exhibit elemental increases. This relationship could be achieved by the dissolution of fossil bone along surfaces, exposing deeper portions of the bone with higher concentrations of a particular element. The sampled area of the post-dissolution bone would then exhibit an increase in the concentration of that element as comparatively depleted regions were dissolved and released into solution. The dissolution of the bone itself would account for increases of specific elements in the water chemistry. An alternative interpretation could be the potential to sample an area of the fossil concentrated in a particular element, which could create an artificially increased concentration of that element. Scenario E, where the fossil displays elemental increases and the solution exhibits elemental decreases, could be interpreted as sorption of the element on the fossil, or precipitation of a mineral containing the particular element in or on the bone, or out of solution. Scenario F is similar to D and can be interpreted as elemental increases to the fossil by sampling an area of the fossil concentrated in a particular element, resulting in an increased concentration of that element. These interpretations highlight the complexity of water-fossil interactions and help to frame our understanding of the various dissolution and precipitation processes potentially occurring in the experimental systems.

XRD data provide additional support of results from ICP-MS and thin section analyses. Based on XRD, gypsum was present in all pre-dissolution samples, but absent in all post-dissolution samples. This is strongly indicative of dissolution, especially since gypsum is highly soluble in aqueous solutions [22]. A similar trend was exhibited by barite in F3. Barite is not known to be soluble in traditional acids like hydrochloric acid [23]; however, results from both XRD and ICP-MS analyses suggest barite dissolution within the experiments. The pre-dissolution sample of F3 had the highest detected weight percent of barite at 15.54%, and post-dissolution analyses of F3 at pH 4 had no detected barite, pH 5 had 3.8%, and pH 6 had 7.5%. These trends directly correlate with pH, where the proportion of barite found in each sample increased with increasing pH. In addition to the XRD results, fossil chemistry measured with ICP-MS support barite dissolution, releasing barium into solution. F3 exposed to pH 4 solution was the only sample to exhibit depletion of barium from the fossil sample (loss of 307.69 ppm) and no barite was detected with XRD.

The three apatite phases identified by XRD—hydroxylapatite, fluorapatite, and carbonated fluorapatite—do not exhibit clear trends like observed for gypsum and barite. A potential source of the difference in apatite phases present in pre- and post-dissolution fossils is the degree of dissolution. The outer portions of cortical bone, which are exposed to ambient environmental conditions during fossilization and diagenesis [24], may have had a thermodynamically more stable apatite phase present, such as carbonated fluorapatite [13]. During bone digenesis and fossilization, the interior of the fossil can be more difficult to alter as diffusion gradients are established, restricting the movement of ions into inner portions of the bone or teeth [24]. Based on geochemical differences observed previously in fossil bone and thermodynamic expectations, it is likely that the inner portions of the fossil here contained more of the hydroxylapatite and fluorapatite phases identified with XRD. As the secondary mineral infilling and interior portions of the fossil trabeculae were exposed due to dissolution of infilling secondary minerals like calcite and gypsum, the other apatite phases that were detected in post-dissolution samples were exposed. Loss of secondary phases through dissolution and exposure of other apatite phases found within trabeculae may help to account for the changes in apatite mineralogy observed in pre- and post-dissolution fossils.

Dissolution is also accompanied by changes to apatite crystallinity. The apatite crystallite size and structural order for post-dissolution samples are typically smallest at pH 4 and increase with increasing pH treatments (Table 6). One explanation may be the preferential dissolution of smaller apatite crystallites under high pH conditions, leaving larger crystallites behind. Under low pH conditions, fossils underwent the greatest extent of dissolution. Potentially under the most acidic conditions, dissolution occurs equally to smaller and larger apatite crystallites as they are exposed by removal of secondary minerals, resulting in almost no perceived changes to crystallinity compared to pre-dissolution samples.

XRD peak fitting identified end member apatite compositions, but fossils may not contain perfectly stoichiometric minerals [25]. The data presented provides information on total apatite content of the fossil, but the relationships of the end members and their various locations within the fossil bone are not spatially resolved here or well-understood more broadly for fossils in general. However, the varying apatite phases detected by XRD in pre- and post-dissolution fossils are indicative of changes to the apatite constituents of fossil bone.

**Fig 5:**
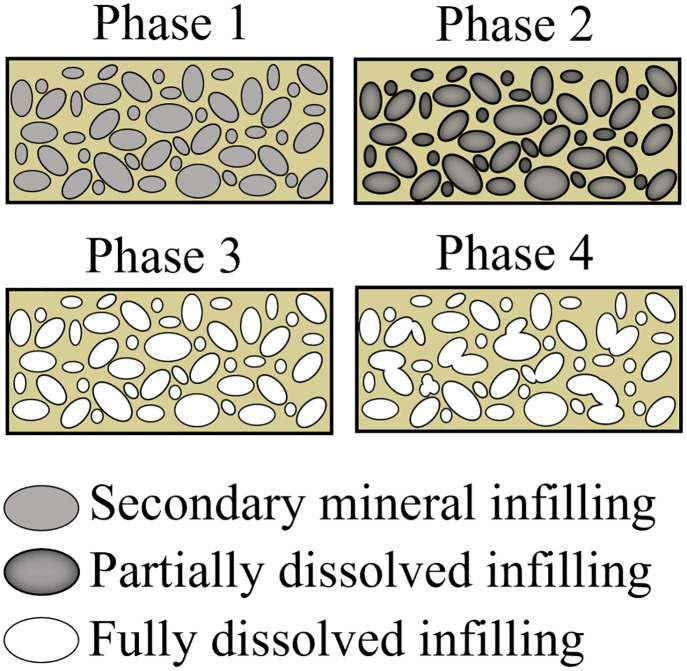
Interpreted effects of dissolution on fossil bone surface area. Cross-sectional schematic diagram demonstrating surface area increases as secondary mineral infilling is dissolved. Phase 1 depicts a fossil with the pore spaces infilled completely by secondary mineral growth. This phase represents the pre-dissolution samples. Phase 2 represents partial dissolution of secondary minerals. Dissolution has not penetrated through the fossil, but dissolution of mineral infilling has created voids that increase pore space, effectively increasing surface area of exposed bone. Phase 3 represents complete dissolution of secondary minerals, and the maximum surface area of the fossil has been attained. Phase 4 is when surface area starts to decrease as pore spaces coalesce.

Exposure to acidic solutions resulted in the partial dissolution of fossil bones, which includes degradation of histologic structures (Fig 4). Degradation was observed in both cortical and trabecular bone in post-dissolution samples, but was more pronounced in trabecular bone. The features observed in post-dissolution thin sections, including fracturing and breakdown of the trabeculae, were not evident in pre-dissolution samples, which strongly supports degradation is due to dissolution rather an artifact from cutting with a rock saw or epoxy resin embedding. A consequence of physical breakdown of the bone as observed in thin section is an increase in surface area. Surface area is an important control on dissolution reaction rates [26]. For bones exposed to acidic precipitation in the field, holding surface area constant in rate estimates may not provide an accurate model based on how dissolution is expected to affect surface area over time. As fossils dissolve, rather than surface area decreasing, therefore decreasing reaction rates, surface area would actually be expected to increase. Because fossils are not one single uniform mineral type, not all phases will dissolve at the same rate. The secondary minerals that infill the porous bone tend to be more soluble than the primary, “biominerals”, or bone itself. As the secondary minerals dissolve and leave the more resistant and highly vascularized trabecular bone as well as fragmented trabecular bone, the surface area increases. Thus, the surface area in dissolving fossils will continue to increase until all infilling secondary minerals have been dissolved; only then will surface area be expected to decrease (Fig 5). The dissolution of infilling secondary minerals followed by dissolution of primary apatite could then potentially lead to pore space coalescence, joining adjacent pores into one large void space. It is possible the initial increase in surface area caused by the preferential removal of secondary mineral phases infilling bone could increase the dissolution rate, but further research is required to understand this relationship, and whether fossil dissolution in general should be considered as a linear or non-linear process.

In addition to surface area controls on rates of dissolution, reaction kinetics are also likely to influence reaction rates in field settings. Dissolution reactions are typically divided into surface-controlled or diffusion-controlled processes. Surface controlled dissolution occurs when pitting or etching happens on the mineral surface. Over time, the pitting will expand and various pits and etches will merge together, creating the driving process for surface-controlled dissolution [21]. Diffusion-controlled processes occur when chemical bonds break, making molecules detach from the mineral surface. When molecular detachment happens faster than diffusion allows the molecules to transfer into bulk solution, a molecular barrier forms at the interface of the water and mineral [17, 21, 27]. Diffusion limitation can occur in systems that are poorly mixed or where fluid movement is restricted. Individual mineralogical studies of calcite, hydroxylapatite, fluorapatite, and carbonated fluorapatite indicate these reactions are primarily surface-controlled [14, 17, 21]. Here, the use of a stir bar ensured continuous water circulation, which should not allow for the molecular barrier to be created, making this experimental system surface-controlled. The surface-controlled nature of these experiments in tandem with the brief time scale of the experiment justifies the linearly-derived dissolution rates (Table 1). However, in a field setting, reaction rates may also be influenced by diffusion-limitation. Evaluating how transferable the results of controlled experiments to the field is the next major challenge to quantifying rates of fossil bone dissolution.

Temperature was also held constant in these experiments to view the effects of pH on fossil dissolution. However, in a real-world setting, temperature will also play a vital role in determining dissolution rates. First, two of the major minerals in this system, calcite and apatite, exhibit retrograde solubility, with dissolution rates increasing at lower temperatures [13, 28]. In natural environmental settings, temperature changes will impact the dissolution rate of minerals depending on the season. Specifically, dissolution driven by abiotic processes would likely occur faster in colder temperatures. In field settings, however, biology would also factor into dissolution. As temperatures begin to warm, biological processes that utilize minerals and elements found within bone will begin to increase [29–31], which could contribute to a faster dissolution rate, even when temperatures are elevated. Bacteria and fungi are known to degrade or bioerode minerals, actively targeting limiting nutrients such as phosphorus by excreting organic acids or other solubilizing compounds [32, 33].

Additional considerations in field settings are freeze-thaw processes, which are expected to result in the development and propagation of cracks in fossils [34]. Cracks would be expected to increase surface area and enhance fluid movement within fossil bone, allowing for fluid to penetrate further into the fossil and potentially dissolve the fossil and/or facilitate the precipitation of secondary minerals within fractures.

In field settings, the dominant acids in precipitation expected to interact with bone are likely carbonic, sulfuric, and nitric, depending on regional atmospheric conditions [35]. The use of hydrochloric acid here was guided by prior studies focused on single-phase mineral dissolution, allowing for direct comparisons with our mixed-phase fossils. While the experimentally-determined dissolution rates presented here do have limitations for real world applications, these results provide an initial, quantitative dissolution rate to help constrain fossil longevity in natural field settings. Exploring the interactions between fossil bone and other types of acids may help to better constrain the responses of fossils to acidic precipitation in the field.

### Implications

The results of this study indicate that fossils exposed at the surface will dissolve when exposed to an acidic solution and provide the first attempt to quantify the rate of fossil bone dissolution. These results, quantifying fossil dissolution rates, have direct importance for multiple areas of paleontology. First, fossil dissolution rates can help field workers better understand the longevity of fossils to make better informed management decisions. Second, gaining quantitative values of fossil dissolution rates can help paleontologists better understand biases in the fossil record. When preservation biases are evaluated in paleontology most commonly the focus is on controls of the fossilization process, such as which types of organisms are more likely to fossilize and what types of environments are more conducive to fossilization. This study shows the importance of examining preservation biases once fossils are exhumed at the surface. Dissolution rates calculated based on experimental data suggest that preservation biases related to potentially rapid dissolution may play a larger role in the fossil record than is currently understood. If the rates calculated here are extrapolated to a fossil with a mass of 100 g and assuming a linear rate, complete dissolution could occur on the order of decades as a first order approximation. Lastly, this study demonstrates a need for further taphonomic research. Similar to preservation biases, when paleontologists refer to taphonomy it is usually in regard to the transition of bone to a fossil within the subsurface [36]; however, the last stage of the fossilization process where the fossils are unearthed is poorly understood. This research suggests the latter portion of taphonomy is swift and active in areas where water-rock interactions occur regularly.

While this study provides a first order estimate of fossil dissolution rates, rates can be refined by examining other variables that affect fossil dissolution. Potential avenues of future research include examining the effects of changing surface area, differing fossil shapes, differing fossil skeletal elements, taxonomic variation, temperature variation, differing acids, salinity, or sediment type on fossil dissolution rates. Additionally, future research should explore non-destructive thermodynamic modeling. If the fossil bone mineralogical composition can be non-destructively determined, along with the geochemical composition of local precipitation or water, thermodynamic modeling can be conducted to determine theoretical dissolution rates. Should simulations closely match the experimentally-derived data, this could potentially eliminate the need for future destructive analyses on non-renewable fossil resources.

## Conclusions

Our results demonstrate that fossil bones exhibit dissolution due to exposure to acidic solutions and that dissolution is accompanied by degradation of histological structures. The evidence that points toward fossil dissolution include overall mass loss, XRD analyses showing mineralogical changes to post-dissolution fossil samples, ICP-MS analyses showing elemental increases likely from fossil bone dissolution into the post-dissolution aqueous solution, and direct damage to bone observed through degradation of histologic structures seen in thin section.

The current dissolution rates have impacts for how preservation biases and taphonomy are evaluated in paleontology. It is important to understand the likelihood of preservation not just during burial and lithification, but after exhumation. Future research could indicate certain types of fossils may dissolve more easily than others, which would reveal yet another type of preservation bias within the fossil record. Similarly, taphonomy does not stop once bone has been chemically altered to fossil. Fossils unearthed at the surface are experiencing the last stage of taphonomy where the fossil is destroyed, in this instance from weathering processes like acidic precipitation. Due to the lack of dissolution rates within the literature, this indicates a need for further research on this stage of taphonomy.

To summarize, this research provides the first quantitative approach towards determining fossil bone dissolution due to exposure to acidic aqueous solutions. Based on current pH trends of precipitation throughout the United States, these values provide a representative approach to understanding fossil dissolution throughout the country. These results provide new insights into taphonomic processes, and a better understanding of preservation biases which aid in fossil conservation efforts.

## Supporting information

S1 Appendix Hourly experiment pH records. (DOCX)

S2 Appendix Complete ICP-MS datasets. (DOCX)

S3 Appendix XRD diffractograms. (DOCX)

S4 Appendix Additional thin section images under plane and cross polarized light. (DOCX)

S5 Appendix R code used in data analysis. (DOCX)
