# Supplementary material for: Experimental dissolution of fossil bone under variable pH conditions
Source: PLoS One. 2022 Oct 13;17(10):e0274084. doi: 10.1371/journal.pone.0274084 (PMC9560490; doi:10.1371/journal.pone.0274084)
Supplement: S1 Appendix — (DOCX) [file pone.0274084.s001.docx]

| **pH 4 Station 1** | | | | | | |
| --- | --- | --- | --- | --- | --- | --- |
| **Date** | **Initial Time** | **Initial pH** | **HCl added (mL)** | **Final Time** | **Final pH** | **Notes** |
| 10-May | 8:00 | - | 22.00 | ~9:00 | 4.00 | Finished set up @ 9:00AM |
|  | 10:01 | 4.91 | 2.00 | 10:01 | 3.76 |  |
|  | 12:00 | 5.08 | 1.75 | 12:01 | 3.80 |  |
|  | 14:00 | 5.02 | 1.25 | 14:02 | 3.95 |  |
|  | 16:00 | 5.02 | 1.10 | 16:01 | 3.99 |  |
|  | 18:00 | 4.97 | 1.00 | 18:01 | 4.00 |  |
| 11-May | 8:00 | 5.72 | 3.25 | 8:02 | 3.95 |  |
|  | 10:00 | 4.87 | 1.75 | 10:02 | 4.00 |  |
|  | 12:00 | 4.88 | 1.25 | 12:01 | 3.99 |  |
|  | 14:00 | 4.85 | 1.25 | 14:02 | 4.00 |  |
|  | 16:00 | 4.80 | 1.50 | 16:01 | 3.97 |  |
|  | 18:00 | 4.78 | 1.50 | 18:01 | 3.99 |  |
| 12-May | 8:00 | 5.58 | 3.25 | 8:01 | 3.96 |  |
|  | 10:00 | 4.68 | 1.25 | 10:00 | 3.92 |  |
|  | 12:00 | 4.67 | 1.25 | 12:00 | 3.97 |  |
|  | 14:00 | 4.68 | 1.25 | 14:00 | 4.00 |  |
|  | 16:00 | 4.65 | 1.00 | 16:00 | 3.96 |  |
|  | 18:00 | 4.63 | 1.25 | 18:01 | 3.97 |  |
| 13-May | 8:00 | 5.43 | 3.00 | 8:00 | 3.98 |  |
|  | 10:00 | 4.55 | 1.00 | 10:01 | 3.99 |  |
|  | 12:00 | 4.56 | 1.50 | 12:01 | 3.98 |  |
|  | 14:00 | 4.55 | 1.50 | 14:01 | 3.92 |  |
|  | 16:00 | 4.51 | 1.25 | 16:01 | 3.98 |  |
|  | 18:00 | 4.54 | 1.25 | 18:01 | 3.99 |  |
| 14-May | 8:00 | 5.36 | 3.00 | 8:01 | 3.93 | Overshot |
|  | 10:00 | 4.45 | 1.25 | 10:01 | 3.81 |  |
|  | 12:00 | 4.38 | 1.00 | 12:01 | 3.97 |  |
|  | 14:00 | 4.50 | 1.00 | 14:00 | 3.98 |  |
|  | 16:00 | 4.50 | 1.25 | 16:01 | 3.96 |  |
|  | 18:00 | 4.47 | 1.25 | 18:01 | 4.00 |  |
| 15-May | 8:00 | 5.26 | 3.00 | 8:02 | 3.97 |  |
|  | 10:00 | 4.43 | 1.25 | 10:01 | 3.99 |  |
|  | 12:00 | 4.44 | 1.00 | 12:01 | 4.00 |  |
|  | 14:00 | 4.44 | 1.00 | 14:01 | 3.96 |  |
|  | 16:00 | 4.41 | 1.00 | 16:00 | 3.98 |  |
|  | 18:00 | 4.43 | 1.00 | 18:00 | 4.00 |  |
| 16-May | 8:00 | 5.18 | 3.00 | 8:01 | 3.98 |  |
|  | 10:00 | 4.39 | 1.00 | 10:01 | 3.97 |  |
|  | 12:00 | 4.39 | 1.00 | 12:01 | 3.97 |  |
|  | 14:00 | 4.39 | 1.00 | 14:01 | 3.99 |  |
|  | 16:00 | 4.39 | 1.00 | 16:01 | 4.00 |  |
|  | 18:00 | 4.39 | 1.00 | 18:00 | 3.98 |  |
| 17-May | 8:00 | 5.11 | 3.00 | 8:01 | 3.90 |  |
|  | 10:00 | 4.31 | 1.00 | 10:01 | 3.91 |  |
|  | 12:00 | 4.31 | 1.00 | 12:00 | 3.97 |  |
|  | 14:00 | 4.33 | 1.00 | 14:00 | 3.97 |  |
|  | 16:00 | 4.35 | 1.00 | 16:01 | 3.99 |  |
|  | 18:00 | 4.35 | 1.00 | 18:01 | 3.96 |  |
| **pH 4 Station 1 (Continued)** | | | | | | |
| **Date** | **Initial Time** | **Initial pH** | **HCl added (mL)** | **Final Time** | **Final pH** | **Notes** |
| 18-May | 8:00 | 5.09 | 2.75 | 8:03 | 3.99 |  |
|  | 10:00 | 4.32 | 1.00 | 10:00 | 4.00 |  |
|  | 12:00 | 4.32 | 1.00 | 12:01 | 3.96 |  |
|  | 14:00 | 4.29 | 1.00 | 14:01 | 4.00 |  |
|  | 16:00 | 4.32 | 1.00 | 16:00 | 4.00 |  |
|  | 18:00 | 4.34 | 1.00 | 18:00 | 3.99 |  |
| 19-May | 8:00 | 5.05 | 2.50 | 8:01 | 3.96 |  |
|  | 10:00 | 4.30 | 1.00 | 10:02 | 3.95 |  |
|  | 12:00 | 4.26 | 1.00 | 12:01 | 3.94 |  |
|  | 14:00 | 4.25 | 0.75 | 14:01 | 3.99 |  |
|  | 16:00 | 4.28 | 0.75 | 16:01 | 3.97 |  |
|  | 18:00 | 4.26 | 1.00 | 18:00 | 3.98 |  |
| 20-May | 8:00 | 4.98 | 2.75 | 8:03 | 3.99 | Below pH 5 over night |
|  | 10:00 | 4.29 | 1.00 | 10:00 | 3.95 |  |
|  | 12:00 | 4.24 | 0.75 | 12:00 | 3.99 |  |
|  | 14:00 | 4.26 | 0.75 | 14:01 | 4.00 |  |
|  | 16:00 | 4.29 | 0.75 | 16:00 | 4.00 |  |
|  | 18:00 | 4.27 | 0.75 | 18:00 | 3.94 |  |
| 21-May | 8:00 | 4.93 | 2.75 | 8:01 | 4.00 |  |
|  | 10:00 | 4.30 | 1.00 | 10:01 | 4.00 |  |
|  | 12:00 | 4.25 | 0.75 | 12:00 | 3.97 |  |
|  | 14:00 | 4.24 | 1.00 | 14:02 | 3.99 |  |
|  | 16:00 | 4.24 | 1.00 | 16:01 | 3.97 |  |
|  | 18:00 | 4.24 | 1.00 | 18:00 | 3.92 |  |
| 22-May | 8:00 | 4.89 | 2.50 | 8:02 | 4.00 | Removed ~40mL of solution from jar (jar was too full); pH= 4.30 after removal, then overshot trying to lower pH No acid added at 12:00 |
|  | 10:00 | 4.27 | 1.00 | 10:07 | 3.70 |  |
|  | 12:00 | 3.99 | - | - | - |  |
|  | 14:00 | 4.27 | 0.75 | 14:01 | 4.00 |  |
|  | 16:00 | 4.25 | 0.75 | 16:00 | 4.00 |  |
|  | 18:00 | 4.24 | 0.75 | 18:00 | 4.00 |  |
| 23-May | 8:00 | 4.89 | 2.50 | 8:02 | 3.99 | Left early due to storm |
|  | 10:00 | 4.19 | 1.00 | 10:00 | 3.93 |  |
|  | 12:00 | 4.17 | 0.50 | 12:00 | 3.96 |  |
|  | 14:00 | 4.20 | 0.50 | 14:00 | 3.98 |  |
|  | 16:00 | 4.17 | 0.50 | 16:00 | 3.94 |  |
|  | 17:53 | 4.17 | 0.50 | 17:55 | 3.97 |  |
| 24-May | 8:00 | 4.78 | 2.50 | 8:03 | 3.87 | Overshot |
|  | 10:00 | 4.12 | 0.25 | 10:00 | 3.99 |  |
|  | 12:00 | 4.23 | 0.75 | 12:00 | 3.99 |  |
|  | 14:00 | 4.18 | 0.50 | 14:01 | 3.97 |  |
|  | 16:00 | 4.90 | 0.50 | 16:01 | 4.00 |  |
|  | 18:00 | 4.23 | 0.75 | 18:01 | 3.96 |  |
| **pH 4 Station 1 (Continued)** | | | | | | |
| **Date** | **Initial Time** | **Initial pH** | **HCl added (mL)** | **Final Time** | **Final pH** | **Notes** |
| 25-May | 8:01 | 4.83 | 2.25 | 8:02 | 3.97 |  |
|  | 10:00 | 4.19 | 0.50 | 10:01 | 4.00 |  |
|  | 12:00 | 4.21 | 1.00 | 12:01 | 3.98 |  |
|  | 14:00 | 4.19 | 0.75 | 14:01 | 3.93 |  |
|  | 16:00 | 4.15 | 0.50 | 16:01 | 4.00 |  |
|  | 18:00 | 4.21 | 0.75 | 18:00 | 4.00 |  |
| 26-May | 8:00 | 4.81 | 2.00 | 8:01 | 3.97 |  |
|  | 10:00 | 4.21 | 0.75 | 10:01 | 3.99 |  |
|  | 12:00 | 4.20 | 0.75 | 12:01 | 3.93 |  |
|  | 14:00 | 4.13 | 0.25 | 14:01 | 4.00 |  |
|  | 16:00 | 4.19 | 0.50 | 16:01 | 4.00 |  |
|  | 18:00 | 4.20 | 0.75 | 18:00 | 3.97 |  |
| 27-May | 8:00 | 4.77 | 2.50 | 8:02 | 3.93 |  |
|  | 10:00 | 4.14 | 0.50 | 10:02 | 3.97 |  |
|  | 12:00 | 4.16 | 0.75 | 12:01 | 3.99 |  |
|  | 14:00 | 4.16 | 0.50 | 14:01 | 3.96 |  |
|  | 16:00 | 4.16 | 0.50 | 16:00 | 3.98 |  |
|  | 18:00 | 4.16 | 0.75 | 18:00 | 3.99 |  |
| 28-May | 8:00 | 4.77 | 2.50 | 8:01 | 3.96 |  |
|  | 10:00 | 4.14 | 0.50 | 10:00 | 3.97 |  |
|  | 12:00 | 4.17 | 0.50 | 12:01 | 3.99 |  |
|  | 14:00 | 4.17 | 0.50 | 14:00 | 3.98 |  |
|  | 16:00 | 4.15 | 0.50 | 16:00 | 3.99 |  |
|  | 18:00 | 4.17 | 0.50 | 18:01 | 3.95 |  |
| 29-May | 8:00 | 4.73 | 2.50 | 8:02 | 3.95 | Overshot  Barely pressed plunger |
|  | 10:00 | 4.15 | 0.50 | 10:00 | 3.92 |  |
|  | 12:00 | 4.12 | 0.50 | 12:00 | 3.85 |  |
|  | 14:00 | 4.05 | <0.25 | 14:00 | 3.98 |  |
|  | 16:00 | 4.15 | 0.25 | 16:00 | 3.99 |  |
|  | 18:00 | 4.15 | 0.25 | 18:00 | 3.97 |  |
| 30-May | 8:00 | 4.73 | 1.75 | 8:01 | 3.88 | Overshot |
|  | 10:00 | 4.05 | 0.25 | 10:00 | 3.97 |  |
|  | 12:00 | 4.15 | 0.25 | 12:00 | 3.97 |  |
|  | 14:00 | 4.14 | 0.25 | 14:00 | 3.97 |  |
|  | 16:00 | 4.15 | 0.25 | 16:00 | 3.99 |  |
|  | 18:00 | 4.14 | 0.25 | 18:01 | 3.99 |  |

| **pH 4 Station 2** | | | | | | |  |  |
| --- | --- | --- | --- | --- | --- | --- | --- | --- |
| **Date** | **Initial Time** | **Initial pH** | **HCl added (mL)** | **Final Time** | **Final pH** | **Notes** |  |  |
| 10-May | 8:00 | - | 21.00 | ~9:00 | 3.99 | Finished set up ~9:00 AM |  |  |
|  | 10:04 | 4.85 | 1.50 | 10:05 | 3.91 |  |  |  |
|  | 12:02 | 4.96 | 1.25 | 12:03 | 3.94 |  |  |  |
|  | 14:03 | 4.85 | 1.25 | 14:04 | 3.98 |  |  |  |
|  | 16:02 | 4.89 | 1.10 | 16:03 | 3.98 |  |  |  |
|  | 18:02 | 4.86 | 1.00 | 18:04 | 3.99 |  |  |  |
| 11-May | 8:03 | 5.67 | 2.50 | 8:05 | 3.98 |  |  |  |
|  | 10:02 | 4.76 | 1.00 | 10:04 | 3.94 |  |  |  |
|  | 12:02 | 4.73 | 1.25 | 12:04 | 3.99 |  |  |  |
|  | 14:02 | 4.72 | 1.25 | 14:04 | 3.91 |  |  |  |
|  | 16:01 | 4.63 | 1.00 | 16:03 | 3.98 |  |  |  |
|  | 18:02 | 4.67 | 0.75 | 18:04 | 3.97 |  |  |  |
| 12-May | 8:02 | 5.53 | 2.00 | 8:03 | 3.94 | Overshot |  |  |
|  | 10:01 | 4.53 | 1.00 | 10:02 | 3.99 |  |  |  |
|  | 12:01 | 4.58 | 1.00 | 12:02 | 3.94 |  |  |  |
|  | 14:01 | 4.53 | 1.00 | 14:02 | 3.94 |  |  |  |
|  | 16:01 | 4.51 | 1.25 | 16:02 | 3.98 |  |  |  |
|  | 18:01 | 4.53 | 1.25 | 18:03 | 3.82 |  |  |  |
| 13-May | 8:01 | 5.39 | 2.00 | 8:02 | 3.98 |  |  |  |
|  | 10:02 | 4.46 | 1.00 | 10:03 | 3.97 |  |  |  |
|  | 12:02 | 4.45 | 1.00 | 12:02 | 3.97 |  |  |  |
|  | 14:01 | 4.45 | 1.00 | 14:02 | 3.95 |  |  |  |
|  | 16:01 | 4.42 | 1.00 | 16:02 | 3.94 |  |  |  |
|  | 18:01 | 4.40 | 1.00 | 18:02 | 3.92 |  |  |  |
| 14-May | 8:02 | 5.32 | 2.00 | 8:03 | 3.97 | Overshot |  |  |
|  | 10:01 | 4.38 | 1.00 | 10:02 | 3.83 |  |  |  |
|  | 12:01 | 4.27 | 0.50 | 12:02 | 4.00 |  |  |  |
|  | 14:01 | 4.40 | 1.00 | 14:02 | 3.97 |  |  |  |
|  | 16:02 | 4.38 | 1.00 | 16:02 | 3.97 |  |  |  |
|  | 18:02 | 4.38 | 1.00 | 18:03 | 3.95 |  |  |  |
| 15-May | 8:03 | 5.24 | 2.00 | 8:04 | 3.99 |  |  |  |
|  | 10:02 | 4.37 | 1.00 | 10:02 | 3.90 |  |  |  |
|  | 12:01 | 4.28 | 0.75 | 12:02 | 3.97 |  |  |  |
|  | 14:02 | 4.34 | 1.00 | 14:02 | 3.97 |  |  |  |
|  | 16:01 | 4.34 | 1.00 | 16:02 | 3.92 |  |  |  |
|  | 18:01 | 4.29 | 0.50 | 18:02 | 3.99 |  |  |  |
| 16-May | 8:01 | 5.16 | 2.00 | 8:02 | 3.91 |  |  |  |
|  | 10:01 | 4.25 | 0.75 | 10:02 | 3.98 |  |  |  |
|  | 12:01 | 4.32 | 0.75 | 12:03 | 3.97 |  |  |  |
|  | 14:02 | 4.31 | 0.75 | 14:03 | 3.94 |  |  |  |
|  | 16:01 | 4.28 | 0.75 | 16:02 | 3.98 |  |  |  |
|  | 18:01 | 4.32 | 0.75 | 18:02 | 3.93 |  |  |  |
| **pH 4 Station 2 (Continued)** | | | | | | |  |  |
| **Date** | **Initial Time** | **Initial pH** | **HCl added (mL)** | **Final Time** | **Final pH** | **Notes** |  |  |
| 17-May | 8:02 | 5.11 | 2.00 | 8:03 | 3.88 | Overshot |  |  |
|  | 10:01 | 4.19 | 0.50 | 10:02 | 4.00 |  |  |  |
|  | 12:01 | 4.30 | 0.75 | 12:02 | 3.98 |  |  |  |
|  | 14:01 | 4.28 | 0.75 | 14:02 | 3.98 |  |  |  |
|  | 16:01 | 4.28 | 0.75 | 16:02 | 4.00 |  |  |  |
|  | 18:01 | 4.30 | 0.75 | 18:02 | 3.97 |  |  |  |
| 18-May | 8:03 | 5.06 | 1.75 | 8:04 | 3.88 | Overshot  Overshot 16:01, needs less acid |  |  |
|  | 10:01 | 4.16 | 0.50 | 10:01 | 3.93 |  |  |  |
|  | 12:01 | 4.22 | 0.75 | 12:03 | 3.98 |  |  |  |
|  | 14:01 | 4.25 | 1.00 | 14:02 | 3.98 |  |  |  |
|  | 16:01 | 4.26 | 1.00 | 16:01 | 3.86 |  |  |  |
|  | 18:01 | 4.16 | 0.50 | 18:02 | 3.94 |  |  |  |
| 19-May | 8:02 | 4.99 | 1.25 | 8:03 | 3.98 | Below pH 5 overnight |  |  |
|  | 10:02 | 4.25 | 0.50 | 10:03 | 3.99 |  |  |  |
|  | 12:01 | 4.24 | 0.75 | 12:03 | 3.97 |  |  |  |
|  | 14:01 | 4.23 | 0.75 | 14:02 | 3.97 |  |  |  |
|  | 16:01 | 4.22 | 0.50 | 16:02 | 3.95 |  |  |  |
|  | 18:01 | 4.21 | 0.50 | 18:01 | 3.98 |  |  |  |
| 20-May | 8:03 | 4.95 | 1.50 | 8:05 | 3.90 |  |  |  |
|  | 10:01 | 4.14 | 0.25 | 10:02 | 3.99 |  |  |  |
|  | 12:01 | 4.22 | 0.75 | 12:02 | 3.99 |  |  |  |
|  | 14:02 | 4.22 | 0.75 | 14:03 | 3.91 |  |  |  |
|  | 16:01 | 4.15 | 0.50 | 16:02 | 3.94 |  |  |  |
|  | 18:01 | 4.18 | 0.50 | 18:01 | 3.97 |  |  |  |
| 21-May | 8:01 | 4.19 | 1.50 | 8:03 | 3.97 |  |  |  |
|  | 10:02 | 4.24 | 0.75 | 10:02 | 3.92 |  |  |  |
|  | 12:01 | 4.14 | 0.50 | 12:01 | 3.99 |  |  |  |
|  | 14:02 | 4.21 | 0.75 | 14:03 | 4.00 |  |  |  |
|  | 16:02 | 4.21 | 0.50 | 16:03 | 3.97 |  |  |  |
|  | 18:01 | 4.18 | 0.50 | 18:01 | 3.97 |  |  |  |
| 22-May | 8:02 | 4.87 | 2.00 | 8:03 | 3.91 | Removed ~12mL from solution pH=4.14 after |  |  |
|  | 10:08 | 4.14 | 0.50 | 10:14 | 3.97 |  |  |  |
|  | 12:01 | 4.16 | 0.25 | 12:01 | 4.00 |  |  |  |
|  | 14:01 | 4.22 | 0.50 | 14:02 | 4.00 |  |  |  |
|  | 16:01 | 4.19 | 0.50 | 16:01 | 3.97 |  |  |  |
|  | 18:01 | 4.18 | 0.50 | 18:02 | 3.98 |  |  |  |
| 23-May | 8:02 | 4.84 | 1.25 | 8:03 | 3.98 | Left early due to storm |  |  |
|  | 10:01 | 4.17 | 0.50 | 10:01 | 3.98 |  |  |  |
|  | 12:00 | 4.18 | 0.50 | 12:01 | 3.98 |  |  |  |
|  | 14:01 | 4.19 | 0.50 | 14:01 | 3.98 |  |  |  |
|  | 16:00 | 4.18 | 0.50 | 16:01 | 3.98 |  |  |  |
|  | 17:56 | 4.18 | 0.50 | 17:56 | 3.95 |  |  |  |
| **pH 4 Station 2 (Continued)** | | | | | | |  |  |
| **Date** | **Initial Time** | **Initial pH** | **HCl added (mL)** | **Final Time** | **Final pH** | **Notes** |  |  |
| 24-May | 8:03 | 4.81 | 1.25 | 8:04 | 3.99 |  |  |  |
|  | 10:01 | 4.19 | 0.50 | 10:02 | 3.97 |  |  |  |
|  | 12:01 | 4.16 | 0.50 | 12:01 | 4.00 |  |  |  |
|  | 14:01 | 4.18 | 0.50 | 14:02 | 3.95 |  |  |  |
|  | 16:01 | 4.14 | 0.50 | 16:02 | 3.97 |  |  |  |
|  | 18:02 | 4.15 | 0.50 | 18:03 | 3.95 |  |  |  |
| 25-May | 8:03 | 4.77 | 1.25 | 8:04 | 3.99 |  |  |  |
|  | 10:01 | 4.18 | 0.50 | 10:02 | 4.00 |  |  |  |
|  | 12:01 | 4.18 | 0.50 | 12:02 | 3.96 |  |  |  |
|  | 14:01 | 4.14 | 0.50 | 14:02 | 4.00 |  |  |  |
|  | 16:01 | 4.17 | 0.75 | 16:02 | 4.00 |  |  |  |
|  | 18:01 | 4.18 | 0.75 | 18:02 | 3.94 |  |  |  |
| 26-May | 8:03 | 4.74 | 1.50 | 8:04 | 3.94 |  | |  |
|  | 10:01 | 4.12 | 0.25 | 10:02 | 4.00 |  |  |  |
|  | 12:02 | 4.18 | 0.50 | 12:03 | 3.98 |  |  |  |
|  | 14:02 | 4.15 | 0.50 | 14:03 | 3.98 |  |  |  |
|  | 16:02 | 4.14 | 0.50 | 16:03 | 3.99 |  |  |  |
|  | 18:01 | 4.15 | 0.50 | 18:02 | 3.97 |  |  |  |
| 27-May | 8:02 | 4.73 | 1.50 | 8:03 | 3.97 |  | |  |
|  | 10:02 | 4.14 | 0.50 | 10:05 | 4.00 |  |  |  |
|  | 12:02 | 4.16 | 0.50 | 12:02 | 3.94 |  |  |  |
|  | 14:01 | 4.10 | 0.25 | 14:02 | 4.00 |  |  |  |
|  | 16:01 | 4.15 | 0.50 | 16:02 | 3.98 |  |  |  |
|  | 18:00 | 4.14 | 0.50 | 18:01 | 3.92 |  |  |  |
| 28-May | 8:01 | 4.67 | 1.50 | 8:03 | 3.99 |  | |  |
|  | 10:01 | 4.16 | 0.50 | 10:02 | 3.99 |  |  |  |
|  | 12:01 | 4.14 | 0.50 | 12:02 | 3.97 |  |  |  |
|  | 14:01 | 4.12 | 0.25 | 14:01 | 4.00 |  |  |  |
|  | 16:01 | 4.15 | 0.50 | 16:02 | 3.96 |  |  |  |
|  | 18:01 | 4.12 | 0.50 | 18:02 | 3.97 |  |  |  |
| 29-May | 8:02 | 4.67 | 1.50 | 8:03 | 3.99 |  | |  |
|  | 10:01 | 4.15 | 0.25 | 10:02 | 3.98 |  |  |  |
|  | 12:00 | 4.13 | 0.25 | 12:01 | 3.96 |  |  |  |
|  | 14:01 | 4.12 | 0.25 | 14:01 | 3.95 |  |  |  |
|  | 16:00 | 4.10 | 0.25 | 16:01 | 3.93 |  |  |  |
|  | 18:01 | 4.08 | 0.25 | 18:01 | 3.98 |  |  |  |
| 30-May | 8:02 | 4.65 | 1.00 | 8:03 | 3.94 | Overshot  Barely pressed plunger | | |
|  | 10:02 | 4.11 | 0.25 | 10:01 | 3.88 |  |  |  |
|  | 12:02 | 4.03 | <0.25 | 12:01 | 3.98 |  |  |  |
|  | 14:01 | 4.12 | 0.25 | 14:01 | 3.95 |  |  |  |
|  | 16:01 | 4.09 | 0.25 | 16:01 | 3.96 |  |  |  |
|  | 18:01 | 4.10 | 0.25 | 18:02 | 3.94 |  |  |  |

| **pH 4 Station 3** | | | | | | |
| --- | --- | --- | --- | --- | --- | --- |
| **Date** | **Initial Time** | **Initial pH** | **HCl added (mL)** | **Final Time** | **Final pH** | **Notes** |
| 10-May | 8:00 | - | 20.00 | ~9:00 | 4.01 | Finished set up ~9:00AM |
|  | 10:06 | 4.57 | 1.00 | 10:08 | 3.84 |  |
|  | 12:04 | 4.49 | 0.75 | 12:05 | 3.93 |  |
|  | 14:04 | 4.54 | 1.00 | 14:07 | 4.00 |  |
|  | 16:04 | 4.56 | 0.75 | 16:05 | 4.00 |  |
|  | 18:05 | 4.56 | 1.00 | 18:07 | 3.95 |  |
| 11-May | 8:06 | 5.37 | 2.00 | 8:07 | 3.96 | Overshot, needs less acid already |
|  | 10:05 | 4.35 | 0.50 | 10:06 | 3.97 |  |
|  | 12:04 | 4.41 | 0.75 | 12:06 | 3.96 |  |
|  | 14:05 | 4.38 | 0.50 | 14:06 | 3.99 |  |
|  | 16:04 | 4.39 | 1.00 | 16:05 | 3.98 |  |
|  | 18:05 | 4.39 | 1.00 | 18:06 | 3.87 |  |
| 12-May | 8:04 | 5.18 | 2.25 | 8:06 | 3.93 | Overshot |
|  | 10:03 | 4.21 | 0.75 | 10:04 | 4.00 |  |
|  | 12:02 | 4.32 | 1.00 | 12:04 | 3.96 |  |
|  | 14:03 | 4.29 | 0.75 | 14:04 | 3.97 |  |
|  | 16:03 | 4.30 | 0.50 | 16:04 | 4.00 |  |
|  | 18:04 | 4.31 | 0.75 | 18:05 | 3.81 |  |
| 13-May | 8:04 | 5.07 | 1.75 | 8:04 | 3.99 |  |
|  | 10:04 | 4.22 | 0.50 | 10:05 | 3.93 |  |
|  | 12:03 | 4.21 | 0.50 | 12:04 | 3.98 |  |
|  | 14:03 | 4.26 | 0.50 | 14:03 | 3.99 |  |
|  | 16:02 | 4.26 | 0.75 | 16:03 | 3.98 |  |
|  | 18:02 | 4.25 | 0.75 | 18:04 | 3.93 |  |
| 14-May | 8:03 | 4.98 | 1.75 | 8:05 | 3.95 | Stayed under pH 5 overnight.; Overshot |
|  | 10:03 | 4.14 | 0.50 | 10:04 | 3.79 |  |
|  | 12:03 | 4.05 | 0.25 | 12:03 | 3.98 |  |
|  | 14:02 | 4.22 | 0.50 | 14:03 | 4.00 |  |
|  | 16:02 | 4.22 | 0.50 | 16:04 | 3.90 |  |
|  | 18:03 | 4.14 | 0.50 | 18:05 | 3.93 |  |
| 15-May | 8:00 | 4.96 | 1.50 | 8:06 | 4.00 | Overshot Overshot |
|  | 10:00 | 4.17 | 0.75 | 10:04 | 3.95 |  |
|  | 12:00 | 4.17 | 0.50 | 12:03 | 3.87 |  |
|  | 14:00 | 4.09 | 0.40 | 14:04 | 3.89 |  |
|  | 16:00 | 4.11 | 0.25 | 16:03 | 3.98 |  |
|  | 18:00 | 4.19 | 0.50 | 18:04 | 3.98 |  |
| 16-May | 8:03 | 4.87 | 1.25 | 8:04 | 3.98 |  |
|  | 10:03 | 4.13 | 0.25 | 10:03 | 4.00 |  |
|  | 12:03 | 4.18 | 0.50 | 12:04 | 4.00 |  |
|  | 14:03 | 4.19 | 0.30 | 14:04 | 3.96 |  |
|  | 16:02 | 4.15 | 0.30 | 16:03 | 3.95 |  |
|  | 18:02 | 4.13 | 0.25 | 18:03 | 3.99 |  |
| **pH 4 Station 3 (Continued)** | | | | | | |
| **Date** | **Initial Time** | **Initial pH** | **HCl added (mL)** | **Final Time** | **Final pH** | **Notes** |
| 17-May | 8:03 | 4.81 | 1.25 | 8:05 | 3.93 |  |
|  | 10:02 | 4.07 | 0.25 | 10:03 | 3.97 |  |
|  | 12:02 | 4.14 | 0.30 | 12:03 | 3.97 |  |
|  | 14:02 | 4.14 | 0.25 | 14:03 | 3.99 |  |
|  | 16:03 | 4.13 | 0.25 | 16:03 | 3.98 |  |
|  | 18:03 | 4.15 | 0.25 | 18:03 | 3.99 |  |
| 18-May | 8:05 | 4.78 | 1.25 | 3.89 | 3.89 | Overshot Barely tapped plunger   Overshot |
|  | 10:02 | 4.02 | <0.25 | 3.97 | 3.97 |  |
|  | 12:03 | 4.13 | 0.25 | 3.99 | 3.99 |  |
|  | 14:03 | 4.15 | 0.25 | 3.99 | 3.99 |  |
|  | 16:02 | 4.14 | 0.25 | 3.96 | 3.96 |  |
|  | 18:02 | 4.12 | 0.25 | 3.89 | 3.89 |  |
| 19-May | 8:04 | 4.68 | 1.25 | 8:06 | 3.97 |  |
|  | 10:04 | 4.08 | 0.25 | 10:05 | 3.98 |  |
|  | 12:03 | 4.13 | 0.25 | 12:04 | 3.97 |  |
|  | 14:02 | 4.12 | 0.25 | 14:03 | 3.96 |  |
|  | 16:02 | 4.11 | 0.25 | 16:03 | 3.97 |  |
|  | 18:02 | 4.12 | 0.25 | 18:03 | 3.92 |  |
| 20-May | 8:05 | 4.66 | 1.25 | 8:06 | 3.94 | Barely tapped plunger |
|  | 10:02 | 4.04 | <0.25 | 10:03 | 3.95 |  |
|  | 12:03 | 4.09 | 0.25 | 12:03 | 3.96 |  |
|  | 14:03 | 4.09 | 0.25 | 14:04 | 3.95 |  |
|  | 16:02 | 4.09 | 0.25 | 16:03 | 3.94 |  |
|  | 18:02 | 4.08 | 0.25 | 18:02 | 3.96 |  |
| 21-May | 8:03 | 4.62 | 1.00 | 8:04 | 3.97 | Barely tapped plunger |
|  | 10:00 | 4.06 | <0.25 | 10:03 | 4.00 |  |
|  | 12:00 | 4.12 | 0.25 | 12:02 | 3.98 |  |
|  | 14:00 | 4.11 | 0.25 | 14:05 | 4.00 |  |
|  | 16:00 | 4.12 | 0.25 | 16:04 | 3.99 |  |
|  | 18:00 | 4.11 | 0.25 | 18:02 | 3.97 |  |
| 22-May | 8:03 | 4.60 | 1.00 | 8:04 | 3.95 | Removed ~11mL of solution pH=4.03 after  Barely tapped plunger |
|  | 10:15 | 4.04 | <0.25 | 10:19 | 3.97 |  |
|  | 12:02 | 4.07 | 0.25 | 12:03 | 3.92 |  |
|  | 14:03 | 4.05 | 0.25 | 14:04 | 3.97 |  |
|  | 16:02 | 4.10 | 0.25 | 16:03 | 3.90 |  |
|  | 18:02 | 4.03 | <0.25 | 18:03 | 3.95 |  |
| 23-May | 8:03 | 4.60 | 1.00 | 8:05 | 4.00 | Left early due to storm |
|  | 10:02 | 4.09 | 0.25 | 10:03 | 3.98 |  |
|  | 12:01 | 4.09 | 0.25 | 12:02 | 3.97 |  |
|  | 14:02 | 4.09 | 0.25 | 14:02 | 3.95 |  |
|  | 16:02 | 4.07 | 0.25 | 16:02 | 3.96 |  |
|  | 17:57 | 4.08 | 0.25 | 17:57 | 3.95 |  |
| **pH 4 Station 3 (Continued)** | | | | | | |
| **Date** | **Initial Time** | **Initial pH** | **HCl added (mL)** | **Final Time** | **Final pH** | **Notes** |
| 24-May | 8:05 | 4.56 | 1.00 | 8:06 | 3.99 |  |
|  | 10:02 | 4.08 | 0.25 | 10:03 | 4.00 |  |
|  | 12:02 | 4.12 | 0.25 | 12:03 | 3.97 |  |
|  | 14:02 | 4.08 | 0.25 | 14:03 | 3.93 |  |
|  | 16:02 | 4.05 | 0.25 | 16:03 | 3.98 |  |
|  | 18:03 | 4.09 | 0.25 | 18:04 | 3.98 |  |
| 25-May | 8:04 | 4.54 | 1.25 | 8:06 | 3.99 |  |
|  | 10:02 | 4.08 | 0.25 | 10:03 | 3.96 |  |
|  | 12:02 | 4.06 | 0.25 | 12:03 | 3.97 |  |
|  | 14:03 | 4.07 | 0.25 | 14:03 | 4.00 |  |
|  | 16:03 | 4.10 | 0.25 | 16:03 | 3.98 |  |
|  | 18:02 | 4.08 | 0.25 | 18:03 | 3.97 |  |
| 26-May | 8:05 | 4.53 | 1.00 | 8:07 | 3.99 | No acid added |
|  | 10:02 | 4.07 | 0.25 | 10:03 | 3.97 |  |
|  | 12:03 | 4.09 | 0.25 | 12:04 | 3.98 |  |
|  | 14:03 | 4.07 | 0.25 | 14:04 | 3.95 |  |
|  | 16:03 | 4.09 | 0.25 | 16:04 | 3.90 |  |
|  | 18:00 | 4.00 | - | 18:00 | - |  |
| 27-May | 8:04 | 4.53 | 1.00 | 8:04 | 3.98 | Barely tapped plunger |
|  | 10:06 | 4.07 | 0.25 | 10:07 | 3.99 |  |
|  | 12:03 | 4.09 | 0.25 | 12:04 | 3.97 |  |
|  | 14:03 | 4.07 | 0.25 | 14:03 | 3.99 |  |
|  | 16:02 | 4.09 | 0.25 | 16:03 | 3.96 |  |
|  | 18:02 | 4.05 | <0.25 | 18:03 | 3.99 |  |
| 28-May | 8:03 | 4.51 | 1.00 | 8:04 | 3.97 | Barely tapped plunger  Barely tapped plunger |
|  | 10:02 | 4.04 | <0.25 | 10:03 | 3.98 |  |
|  | 12:03 | 4.08 | 0.25 | 12:03 | 3.95 |  |
|  | 14:02 | 4.04 | <0.25 | 14:02 | 3.98 |  |
|  | 16:02 | 4.08 | 0.25 | 16:03 | 4.00 |  |
|  | 18:02 | 4.09 | 0.25 | 18:03 | 3.99 |  |
| 29-May | 8:04 | 4.47 | 0.75 | 8:05 | 3.98 | Barely tapped plunger  Barely tapped plunger  Barely tapped plunger, overshot |
|  | 10:02 | 4.06 | 0.25 | 10:03 | 3.92 |  |
|  | 12:01 | 4.01 | <0.25 | 12:02 | 3.98 |  |
|  | 14:02 | 4.08 | 0.25 | 14:03 | 3.97 |  |
|  | 16:01 | 4.06 | <0.25 | 16:02 | 3.94 |  |
|  | 18:02 | 4.04 | <0.25 | 18:03 | 3.81 |  |
| 30-May | 8:03 | 4.36 | 0.25 | 8:05 | 3.96 | Barely tapped plunger  Barely tapped plunger  Barely tapped plunger Barely tapped plunger  Barely tapped plunger |
|  | 10:02 | 4.03 | <0.25 | 10:03 | 3.97 |  |
|  | 12:02 | 4.05 | <0.25 | 12:02 | 3.99 |  |
|  | 14:02 | 4.08 | <0.25 | 14:02 | 3.97 |  |
|  | 16:02 | 4.08 | <0.25 | 16:03 | 3.95 |  |
|  | 18:03 | 4.03 | <0.25 | 18:04 | 3.96 |  |

| **pH 5 Station 1** | | | | | | |
| --- | --- | --- | --- | --- | --- | --- |
| **Date** | **Initial Time** | **Initial pH** | **HCl added (mL)** | **Final Time** | **Final pH** | **Notes** |
| 1-Jun | 8:00 | - | 17.00 | 8:45 | 4.99 | ~8:45 start Overshot  Had wrong electrode plugged in, 14:00 initial is approximate |
|  | 10:00 | 5.37 | 1.00 | 10:02 | 4.89 |  |
|  | 12:00 | 5.27 | 0.75 | 12:01 | 4.98 |  |
|  | 14:00 | ~5.26 | 0.75 | 14:02 | 4.95 |  |
|  | 16:00 | 5.29 | 0.75 | 16:00 | 5.00 |  |
|  | 18:00 | 5.30 | 0.75 | 18:00 | 5.00 |  |
| 2-Jun | 8:00 | 5.85 | 3.50 | 8:02 | 4.89 | Overshot |
|  | 10:00 | 5.24 | 0.75 | 10:01 | 4.98 |  |
|  | 12:00 | 5.29 | 0.50 | 12:02 | 4.98 |  |
|  | 14:00 | 5.28 | 0.50 | 14:02 | 4.98 |  |
|  | 16:00 | 5.28 | 0.50 | 16:01 | 4.92 |  |
|  | 18:00 | 5.28 | 0.50 | 18:00 | 4.99 |  |
| 3-Jun | 8:00 | 5.82 | 3.25 | 8:02 | 4.93 |  |
|  | 10:00 | 5.27 | 0.50 | 10:02 | 4.94 |  |
|  | 12:00 | 5.21 | 0.50 | 12:00 | 4.93 |  |
|  | 14:00 | 5.21 | 0.50 | 14:01 | 5.00 |  |
|  | 16:00 | 5.26 | 0.50 | 16:00 | 4.94 |  |
|  | 18:00 | 5.23 | 0.50 | 18:00 | 4.98 |  |
| 4-Jun | 8:00 | 5.81 | 3.00 | 8:01 | 4.98 |  |
|  | 10:00 | 5.28 | 0.50 | 10:01 | 4.99 |  |
|  | 12:00 | 5.25 | 0.50 | 12:01 | 4.97 |  |
|  | 14:00 | 5.26 | 0.50 | 14:02 | 5.00 |  |
|  | 16:00 | 5.28 | 0.75 | 16:01 | 4.96 |  |
|  | 18:00 | 5.24 | 0.50 | 18:00 | 4.93 |  |
| 5-Jun | 8:01 | 5.80 | 3.25 | 8:05 | 4.97 |  |
|  | 10:00 | 5.21 | 0.75 | 10:00 | 4.93 |  |
|  | 12:00 | 5.19 | 0.50 | 12:00 | 5.00 |  |
|  | 14:00 | 5.27 | 0.75 | 14:00 | 4.97 |  |
|  | 16:00 | 5.23 | 0.75 | 16:00 | 5.00 |  |
|  | 18:00 | 5.27 | 0.50 | 18:00 | 4.92 |  |
| 6-Jun | 8:00 | 5.80 | 3.50 | 8:01 | 4.97 |  |
|  | 10:00 | 5.27 | 0.50 | 10:01 | 5.00 |  |
|  | 12:00 | 5.27 | 0.50 | 12:00 | 4.97 |  |
|  | 14:00 | 5.19 | 0.75 | 14:00 | 5.00 |  |
|  | 16:00 | 5.22 | 0.50 | 16:00 | 5.00 |  |
|  | 18:00 | 5.22 | 0.50 | 18:00 | 4.97 |  |
| 7-Jun | 8:06 | 5.83 | 3.75 | 8:09 | 4.95 |  |
|  | 10:00 | 5.21 | 0.75 | 10:02 | 5.00 |  |
|  | 12:00 | 5.27 | 0.75 | 12:01 | 4.94 |  |
|  | 14:00 | 5.17 | 0.50 | 14:02 | 4.98 |  |
|  | 16:00 | 5.25 | 0.75 | 16:02 | 4.99 |  |
|  | 18:00 | 5.21 | 0.50 | 18:00 | 4.99 |  |
| 8-Jun | 8:00 | 5.79 | 3.75 | 8:02 | 4.98 | Overshot |
|  | 10:00 | 5.26 | 0.75 | 10:02 | 4.99 |  |
|  | 12:00 | 5.24 | 0.75 | 12:03 | 4.89 |  |
|  | 14:00 | 5.19 | 0.50 | 14:02 | 5.00 |  |
|  | 16:00 | 5.20 | 0.75 | 16:01 | 5.00 |  |
|  | 18:00 | 5.21 | 0.75 | 18:01 | 4.99 |  |

| **pH 5 Station 1 (Continued)** | | | | | | |
| --- | --- | --- | --- | --- | --- | --- |
| **Date** | **Initial Time** | **Initial pH** | **HCl added (mL)** | **Final Time** | **Final pH** | **Notes** |
| 9-Jun | 8:00 | 5.75 | 3.25 | 8:03 | 4.98 | Overshot |
|  | 10:00 | 5.25 | 0.75 | 10:01 | 4.99 |  |
|  | 12:00 | 5.19 | 0.75 | 12:01 | 4.99 |  |
|  | 14:00 | 5.23 | 0.75 | 14:02 | 4.94 |  |
|  | 16:00 | 5.18 | 0.50 | 16:01 | 4.96 |  |
|  | 18:00 | 5.20 | 0.75 | 18:01 | 4.79 |  |
| 10-Jun | 8:00 | 5.69 | 4.00 | 8:04 | 4.98 |  |
|  | 10:00 | 5.22 | 0.50 | 10:01 | 4.98 |  |
|  | 12:00 | 5.21 | 0.50 | 12:01 | 4.99 |  |
|  | 14:01 | 5.16 | 0.75 | 14:03 | 4.99 |  |
|  | 16:00 | 5.16 | 0.50 | 16:01 | 4.98 |  |
|  | 18:00 | 5.21 | 0.50 | 18:00 | 4.98 |  |
| 11-Jun | 8:00 | 5.70 | 4.50 | 8:03 | 4.99 |  |
|  | 10:00 | 5.19 | 0.75 | 10:01 | 4.99 |  |
|  | 12:00 | 5.21 | 1.00 | 12:01 | 4.99 |  |
|  | 14:00 | 5.18 | 0.75 | 14:00 | 4.98 |  |
|  | 16:00 | 5.13 | 0.75 | 16:02 | 5.00 |  |
|  | 18:00 | 5.21 | 0.75 | 18:00 | 5.00 |  |
| 12-Jun | 8:00 | 5.66 | 4.50 | 8:02 | 4.95 |  |
|  | 10:00 | 5.17 | 0.50 | 10:00 | 4.97 |  |
|  | 12:00 | 5.16 | 0.50 | 12:00 | 4.95 |  |
|  | 14:00 | 5.16 | 0.50 | 14:00 | 4.93 |  |
|  | 16:00 | 5.15 | 0.50 | 16:01 | 4.93 |  |
|  | 18:00 | 5.15 | 0.50 | 18:00 | 4.93 |  |
| 13-Jun | 8:00 | 5.65 | 3.00 | 8:00 | 4.97 | Precipitate on basket    Overshot |
|  | 10:00 | 5.16 | 0.50 | 10:00 | 4.94 |  |
|  | 12:00 | 5.17 | 0.75 | 12:01 | 4.92 |  |
|  | 14:00 | 5.13 | 0.50 | 14:00 | 4.93 |  |
|  | 16:00 | 5.12 | 0.50 | 16:01 | 4.87 |  |
|  | 18:00 | 5.11 | 0.50 | 18:00 | 4.90 |  |
| 14-Jun | 8:02 | 5.62 | 3.00 | 8:04 | 4.97 |  |
|  | 10:00 | 5.18 | 0.50 | 10:00 | 4.93 |  |
|  | 12:00 | 5.15 | 0.50 | 12:00 | 4.94 |  |
|  | 14:00 | 5.12 | 0.50 | 14:01 | 4.94 |  |
|  | 16:00 | 5.12 | 0.50 | 16:00 | 4.95 |  |
|  | 18:00 | 5.17 | 0.50 | 18:00 | 4.93 |  |
| 15-Jun | 8:00 | 5.59 | 3.00 | 8:02 | 4.98 |  |
|  | 10:00 | 5.15 | 0.50 | 10:01 | 4.99 |  |
|  | 12:00 | 5.14 | 0.50 | 12:02 | 4.99 |  |
|  | 14:00 | 5.13 | 0.50 | 14:01 | 4.96 |  |
|  | 16:00 | 5.11 | 0.25 | 16:01 | 4.96 |  |
|  | 18:00 | 5.17 | 0.50 | 18:00 | 4.95 |  |
| 16-Jun | 8:00 | 5.58 | 3.00 | 8:02 | 4.98 |  |
|  | 10:00 | 5.18 | 0.50 | 10:01 | 4.99 |  |
|  | 12:00 | 5.12 | 0.50 | 12:01 | 4.99 |  |
|  | 14:00 | 5.12 | 0.50 | 14:00 | 4.96 |  |
|  | 16:00 | 5.10 | 0.50 | 16:00 | 4.96 |  |
|  | 18:00 | 5.12 | 0.50 | 18:00 | 4.95 |  |
| **pH 5 Station 1 (Continued)** | | | | | | |
| **Date** | **Initial Time** | **Initial pH** | **HCl added (mL)** | **Final Time** | **Final pH** | **Notes** |
| 17-Jun | 8:00 | 5.58 | 3.00 | 8:01 | 4.90 | Checked early- Dr. apt. |
|  | 10:00 | 5.10 | 0.50 | 10:01 | 4.92 |  |
|  | 12:00 | 5.11 | 0.50 | 12:02 | 4.97 |  |
|  | 14:00 | 5.11 | 0.50 | 14:02 | 4.95 |  |
|  | 16:00 | 5.10 | 0.50 | 16:01 | 4.96 |  |
|  | 17:40 | 5.12 | 0.50 | 17:41 | 4.91 |  |
| 18-Jun | 8:00 | 5.59 | 3.25 | 8:02 | 4.97 |  |
|  | 10:00 | 5.14 | 0.50 | 10:01 | 5.00 |  |
|  | 12:00 | 5.11 | 0.50 | 12:01 | 4.98 |  |
|  | 14:00 | 5.11 | 0.50 | 14:01 | 4.98 |  |
|  | 16:00 | 5.15 | 0.50 | 16:00 | 4.99 |  |
|  | 18:00 | 5.10 | 0.50 | 18:00 | 4.98 |  |
| 19-Jun | 8:00 | 5.55 | 3.00 | 8:01 | 4.95 |  |
|  | 10:00 | 5.12 | 0.50 | 10:00 | 4.97 |  |
|  | 12:00 | 5.08 | 0.40 | 12:01 | 5.00 |  |
|  | 14:00 | 5.11 | 0.50 | 14:00 | 5.00 |  |
|  | 16:00 | 5.13 | 0.50 | 16:00 | 4.99 |  |
|  | 18:00 | 5.13 | 0.50 | 18:00 | 5.00 |  |
| 20-Jun | 8:01 | 5.57 | 3.00 | 8:03 | 4.98 |  |
|  | 10:00 | 5.13 | 0.50 | 10:00 | 4.94 |  |
|  | 12:00 | 5.07 | 0.50 | 12:00 | 4.93 |  |
|  | 14:00 | 5.06 | 0.50 | 14:01 | 4.99 |  |
|  | 16:00 | 5.11 | 0.50 | 16:01 | 4.95 |  |
|  | 18:00 | 5.08 | 0.50 | 18:01 | 4.92 |  |
| 21-Jun | 8:02 | 5.53 | 3.00 | 8:05 | 5.00 |  |
|  | 10:00 | 5.08 | 0.50 | 10:01 | 4.98 |  |
|  | 12:00 | 5.09 | 0.50 | 12:00 | 4.96 |  |
|  | 14:00 | 5.08 | 0.50 | 14:00 | 4.94 |  |
|  | 16:00 | 5.09 | 0.50 | 16:00 | 4.98 |  |
|  | 18:00 | 5.07 | 0.50 | 18:00 | 4.92 |  |

| **pH 5 Station 2** | | | | | | |
| --- | --- | --- | --- | --- | --- | --- |
| **Date** | **Initial Time** | **Initial pH** | **HCl added (mL)** | **Final Time** | **Final pH** | **Notes** |
| 1-Jun | 8:00 | - | 23.00 | ~8:30 | 4.99 | ~8:30 finished set up    Overshot |
|  | 10:03 | 5.51 | 1.00 | 10:04 | 5.00 |  |
|  | 12:02 | 5.41 | 0.75 | 12:03 | 4.95 |  |
|  | 14:03 | 5.36 | 0.75 | 14:03 | 4.99 |  |
|  | 16:00 | 5.36 | 0.75 | 16:01 | 4.81 |  |
|  | 18:01 | 5.29 | 0.50 | 18:02 | 4.97 |  |
| 2-Jun | 8:02 | 5.90 | 2.25 | 8:04 | 4.90 | Overshot Overshot |
|  | 10:01 | 5.29 | 0.50 | 10:02 | 4.84 |  |
|  | 12:02 | 5.26 | 0.30 | 12:04 | 4.82 |  |
|  | 14:02 | 5.25 | 0.25 | 14:04 | 5.00 |  |
|  | 16:01 | 5.23 | 0.50 | 16:02 | 5.00 |  |
|  | 18:01 | 5.33 | 0.50 | 18:02 | 4.91 |  |
| 3-Jun | 8:02 | 5.88 | 1.75 | 8:04 | 4.94 | Overshot  Overshot   Overshot Overshot |
|  | 10:01 | 5.31 | 0.50 | 10:03 | 4.84 |  |
|  | 12:02 | 5.25 | 0.25 | 12:02 | 4.70 |  |
|  | 14:02 | 5.21 | 0.25 | 14:02 | 5.00 |  |
|  | 16:01 | 5.34 | 0.50 | 16:02 | 4.72 |  |
|  | 18:01 | 5.21 | 0.30 | 18:02 | 4.87 |  |
| 4-Jun | 8:01 | 5.84 | 1.50 | 8:03 | 4.94 | Overshot @10:02 Added acid very slowly, mixing problem? Overshot |
|  | 10:02 | 5.27 | 0.25 | 10:02 | 4.93 |  |
|  | 12:02 | 5.24 | 0.25 | 12:03 | 4.79 |  |
|  | 14:02 | 5.17 | 0.25 | 14:04 | 4.94 |  |
|  | 16:01 | 5.27 | 0.50 | 16:03 | 4.75 |  |
|  | 18:01 | 5.21 | 0.25 | 18:02 | 4.92 |  |
| 5-Jun | 8:05 | 5.89 | 1.50 | 8:07 | 4.37 | Overshot- put the same amount of acid in as yesterday with minimal difference in starting pH… Overhshot  Overshot |
|  | 10:01 | 5.03 | <0.25 | 10:01 | 4.92 |  |
|  | 12:01 | 5.30 | 0.25 | 12:02 | 4.99 |  |
|  | 14:01 | 5.34 | 0.50 | 14:02 | 4.91 |  |
|  | 16:01 | 5.27 | 0.25 | 16:02 | 4.89 |  |
|  | 18:01 | 5.25 | 0.25 | 18:02 | 4.82 |  |
| 6-Jun | 8:01 | 5.87 | 0.75 | 8:03 | 4.86 | Overshot- tilting needle away from electrode helps pH not jump as drastically (septa where needle is inserted is right over the electrode)  Overshot |
|  | 10:01 | 5.23 | 0.25 | 10:02 | 4.82 |  |
|  | 12:00 | 5.25 | 0.25 | 12:01 | 4.95 |  |
|  | 14:03 | 5.30 | 0.25 | 14:04 | 4.90 |  |
|  | 16:01 | 5.26 | 0.25 | 16:02 | 4.93 |  |
|  | 18:01 | 5.30 | 0.25 | 18:01 | 4.76 |  |
| 7-Jun | 8:09 | 5.90 | 1.00 | 8:10 | 4.82 | Overshot Overshot  Overshot |
|  | 10:02 | 5.21 | <0.25 | 10:03 | 4.87 |  |
|  | 12:01 | 5.26 | <0.25 | 12:02 | 4.98 |  |
|  | 14:02 | 5.30 | 0.25 | 14:02 | 4.88 |  |
|  | 16:02 | 5.23 | 0.25 | 16:03 | 4.98 |  |
|  | 18:01 | 5.29 | 0.25 | 18:02 | 4.91 |  |
| 8-Jun | 8:02 | 5.92 | 0.75 | 8:04 | 4.83 | Overshot Overshot |
|  | 10:03 | 5.25 | 0.25 | 10:04 | 4.89 |  |
|  | 12:04 | 5.30 | <0.25 | 12:05 | 4.96 |  |
|  | 14:02 | 5.32 | 0.25 | 14:03 | 4.99 |  |
|  | 16:01 | 5.33 | 0.25 | 16:02 | 4.93 |  |
|  | 18:02 | 5.30 | 0.25 | 18:04 | 4.91 |  |

| **pH 5 Station 2 (Continued)** | | | | | | | |
| --- | --- | --- | --- | --- | --- | --- | --- |
| **Date** | **Initial Time** | **Initial pH** | **HCl added (mL)** | **Final Time** | **Final pH** | **Notes** |  |
| 9-Jun | 8:04 | 5.95 | 0.75 | 8:06 | 4.84 | Overshot    Overshot |  |
|  | 10:01 | 5.26 | 0.25 | 10:02 | 4.95 |  |  |
|  | 12:02 | 5.32 | 0.25 | 12:03 | 4.99 |  |  |
|  | 14:03 | 5.30 | 0.25 | 14:04 | 4.88 |  |  |
|  | 16:02 | 5.26 | 0.25 | 16:03 | 4.95 |  |  |
|  | 18:01 | 5.30 | 0.25 | 18:02 | 4.98 |  |  |
| 10-Jun | 8:05 | 5.94 | 0.75 | 8:06 | 4.71 | Overshot Overshot   Overshot |  |
|  | 10:01 | 5.15 | <0.25 | 10:03 | 4.86 |  |  |
|  | 12:01 | 5.27 | 0.25 | 12:02 | 5.00 |  |  |
|  | 14:03 | 5.33 | 0.25 | 14:05 | 4.96 |  |  |
|  | 16:01 | 5.30 | 0.25 | 16:02 | 4.85 |  |  |
|  | 18:01 | 5.24 | <0.25 | 18:02 | 5.00 |  |  |
| 11-Jun | 8:03 | 5.94 | 0.75 | 8:05 | 4.89 | Overshot  Overshot |  |
|  | 10:01 | 5.26 | <0.25 | 10:02 | 4.86 |  |  |
|  | 12:01 | 5.24 | 0.25 | 12:02 | 4.97 |  |  |
|  | 14:01 | 5.30 | 0.25 | 14:02 | 4.98 |  |  |
|  | 16:02 | 5.32 | 0.25 | 16:03 | 4.93 |  |  |
|  | 18:01 | 5.27 | 0.25 | 18:02 | 4.92 |  |  |
| 12-Jun | 8:03 | 5.92 | 0.75 | 8:04 | 4.91 | Overshot    Overshot |  |
|  | 10:01 | 5.27 | <0.25 | 10:02 | 4.87 |  |  |
|  | 12:00 | 5.24 | <0.25 | 12:01 | 4.93 |  |  |
|  | 14:01 | 5.28 | <0.25 | 14:02 | 4.94 |  |  |
|  | 16:01 | 5.29 | <0.25 | 16:02 | 4.95 |  |  |
|  | 18:01 | 5.28 | <0.25 | 18:02 | 4.89 |  |  |
| 13-Jun | 8:01 | 5.91 | 0.50 | 8:02 | 4.95 | Precipitate on basket  Overshot  Overshot  Overshot   Overshot |  |
|  | 10:00 | 5.28 | 0.25 | 10:01 | 4.85 |  |  |
|  | 12:01 | 5.21 | 0.25 | 12:03 | 4.80 |  |  |
|  | 14:01 | 5.19 | <0.25 | 14:02 | 4.85 |  |  |
|  | 16:01 | 5.09 | <0.25 | 16:02 | 4.92 |  |  |
|  | 18:00 | 5.27 | 0.25 | 18:01 | 4.85 |  |  |
| 14-Jun | 8:05 | 5.89 | 0.50 | 8:06 | 4.61 | Overshot Overshot     Overshot |  |
|  | 10:01 | 5.05 | <0.25 | 10:02 | 4.80 |  |  |
|  | 12:01 | 5.20 | <0.25 | 12:02 | 4.90 |  |  |
|  | 14:02 | 5.26 | 0.25 | 14:02 | 4.99 |  |  |
|  | 16:01 | 5.30 | 0.25 | 16:02 | 4.95 |  |  |
|  | 18:01 | 5.28 | <0.25 | 18:01 | 4.85 |  |  |
| 15-Jun | 8:01 | 5.87 | 0.50 | 8:02 | 4.70 | Overshot  Overshot Overshot  Overshot |  |
|  | 10:03 | 5.11 | <0.25 | 10:04 | 4.99 |  |  |
|  | 12:03 | 5.31 | <0.25 | 12:04 | 4.88 |  |  |
|  | 14:02 | 5.17 | <0.25 | 14:03 | 4.86 |  |  |
|  | 16:01 | 5.23 | <0.25 | 16:02 | 4.96 |  |  |
|  | 18:01 | 5.26 | <0.25 | 18:02 | 4.88 |  |  |
| 16-Jun | 8:02 | 5.86 | 0.50 | 8:03 | 4.77 | Overshot    Overshot   Overshot |  |
|  | 10:01 | 5.12 | <0.25 | 10:02 | 4.98 |  |  |
|  | 12:01 | 5.22 | 0.25 | 12:02 | 4.90 |  |  |
|  | 14:01 | 5.22 | 0.25 | 14:01 | 4.79 |  |  |
|  | 16:01 | 5.15 | <0.25 | 16:01 | 4.94 |  |  |
|  | 18:01 | 5.24 | 0.25 | 18:02 | 4.82 |  |  |
| **pH 5 Station 2 (Continued)** | | | | | | |  |
| **Date** | **Initial Time** | **Initial pH** | **HCl added (mL)** | **Final Time** | **Final pH** | **Notes** |  |
| 17-Jun | 8:02 | 5.84 | 0.50 | 8:03 | 4.70 | Overshot Overshot  Overshot  Checked early- Dr. apt. |  |
|  | 10:02 | 5.06 | <0.25 | 10:04 | 4.67 |  |  |
|  | 12:03 | 5.07 | <0.25 | 12:04 | 4.85 |  |  |
|  | 14:03 | 5.17 | <0.25 | 14:03 | 4.90 |  |  |
|  | 16:02 | 5.20 | <0.25 | 16:02 | 4.99 |  |  |
|  | 17:41 | 5.21 | <0.25 | 17:42 | 4.90 |  |  |
| 18-Jun | 8:03 | 5.84 | 0.50 | 8:03 | 4.53 | Overshot  No acid added, didn't recover from overshoot yet |  |
|  | 10:01 | 4.91 | - | 10:01 | - |  |  |
|  | 12:01 | 5.22 | <0.25 | 12:02 | 4.97 |  |  |
|  | 14:01 | 5.16 | <0.25 | 14:02 | 4.92 |  |  |
|  | 16:00 | 5.20 | <0.25 | 16:01 | 4.90 |  |  |
|  | 18:01 | 5.19 | <0.25 | 18:01 | 4.96 |  |  |
| 19-Jun | 8:02 | 5.84 | 0.25 | 8:03 | 4.88 | Overshot Overshot   Overshot  Overshot |  |
|  | 10:01 | 5.16 | <0.25 | 10:01 | 4.78 |  |  |
|  | 12:01 | 5.12 | <0.25 | 12:02 | 4.91 |  |  |
|  | 14:00 | 5.20 | <0.25 | 14:01 | 4.99 |  |  |
|  | 16:01 | 5.26 | 0.25 | 16:01 | 4.83 |  |  |
|  | 18:01 | 5.12 | <0.25 | 18:02 | 4.79 |  |  |
| 20-Jun | 8:04 | 5.79 | 0.25 | 8:05 | 4.73 | Overshot   Overshot Overshot |  |
|  | 10:00 | 5.04 | <0.25 | 10:01 | 4.97 |  |  |
|  | 12:01 | 5.26 | <0.25 | 12:03 | 4.98 |  |  |
|  | 14:02 | 5.19 | <0.25 | 14:03 | 4.86 |  |  |
|  | 16:02 | 5.15 | <0.25 | 16:03 | 4.69 |  |  |
|  | 18:01 | 5.04 | <0.25 | 18:02 | 5.00 |  |  |
| 21-Jun | 8:06 | 5.82 | 0.25 | 8:09 | 4.94 | Overshot  Overshot Overshot |  |
|  | 10:01 | 5.22 | <0.25 | 10:02 | 4.79 |  |  |
|  | 12:01 | 5.12 | <0.25 | 12:02 | 4.91 |  |  |
|  | 14:01 | 5.19 | <0.25 | 14:01 | 4.85 |  |  |
|  | 16:00 | 5.15 | <0.25 | 16:01 | 4.78 |  |  |
|  | 18:01 | 5.10 | <0.25 | 18:01 | 4.91 |  |  |

| **pH 5 Station 3** | | | | | | |
| --- | --- | --- | --- | --- | --- | --- |
| **Date** | **Initial Time** | **Initial pH** | **HCl added (mL)** | **Final Time** | **Final pH** | **Notes** |
| 1-Jun | 8:00 | - | 21.00 | ~8:15 | 4.98 | ~8:15 start Overshot |
|  | 10:05 | 5.35 | 1.00 | 10:06 | 4.89 |  |
|  | 12:04 | 5.19 | 0.50 | 12:04 | 4.96 |  |
|  | 14:04 | 5.19 | 0.50 | 14:05 | 4.98 |  |
|  | 16:02 | 5.20 | 0.50 | 16:03 | 4.98 |  |
|  | 18:02 | 5.21 | 0.50 | 18:03 | 4.99 |  |
| 2-Jun | 8:05 | 5.67 | 2.25 | 8:08 | 4.99 |  |
|  | 10:03 | 5.18 | 0.50 | 10:04 | 5.00 |  |
|  | 12:05 | 5.18 | 0.50 | 12:07 | 4.99 |  |
|  | 14:04 | 5.18 | 0.50 | 14:06 | 5.00 |  |
|  | 16:03 | 5.18 | 0.50 | 16:04 | 5.00 |  |
|  | 18:03 | 5.19 | 0.50 | 18:04 | 4.97 |  |
| 3-Jun | 8:04 | 5.63 | 2.00 | 8:06 | 4.99 |  |
|  | 10:04 | 5.16 | 0.25 | 10:05 | 4.96 |  |
|  | 12:03 | 5.14 | 0.25 | 12:04 | 4.99 |  |
|  | 14:03 | 5.16 | 0.25 | 14:04 | 5.00 |  |
|  | 16:03 | 5.16 | 0.50 | 16:05 | 4.98 |  |
|  | 18:02 | 5.15 | 0.25 | 18:04 | 4.99 |  |
| 4-Jun | 8:04 | 5.60 | 2.25 | 8:07 | 5.00 | Starting minute not recorded at 14:00 |
|  | 10:03 | 5.15 | 0.50 | 10:05 | 4.98 |  |
|  | 12:04 | 5.14 | 0.50 | 12:05 | 4.96 |  |
|  | 14:03(?) | 5.14 | 0.50 | 14:05 | 4.95 |  |
|  | 16:03 | 5.15 | 0.50 | 16:04 | 4.99 |  |
|  | 18:03 | 5.15 | 0.50 | 18:04 | 5.00 |  |
| 5-Jun | 8:08 | 5.63 | 2.25 | 8:11 | 4.95 | Overshot |
|  | 10:02 | 5.12 | 0.50 | 10:03 | 5.00 |  |
|  | 12:02 | 5.16 | 0.50 | 12:03 | 4.98 |  |
|  | 14:03 | 5.15 | 0.25 | 14:03 | 4.96 |  |
|  | 16:03 | 5.12 | 0.50 | 16:04 | 4.98 |  |
|  | 18:02 | 5.15 | 0.50 | 18:03 | 4.80 |  |
| 6-Jun | 8:04 | 5.55 | 2.00 | 8:06 | 4.98 |  |
|  | 10:03 | 5.11 | 0.25 | 10:04 | 4.98 |  |
|  | 12:02 | 5.11 | 0.25 | 12:03 | 4.97 |  |
|  | 14:05 | 5.13 | 0.50 | 14:06 | 5.00 |  |
|  | 16:02 | 5.14 | 0.50 | 16:04 | 4.99 |  |
|  | 18:02 | 5.16 | 0.50 | 18:03 | 4.95 |  |
| 7-Jun | 8:11 | 5.58 | 2.25 | 8:12 | 4.97 |  |
|  | 10:04 | 5.11 | 0.25 | 10:05 | 4.97 |  |
|  | 12:03 | 5.11 | 0.50 | 12:04 | 4.99 |  |
|  | 14:04 | 5.14 | 0.50 | 14:05 | 4.98 |  |
|  | 16:04 | 5.13 | 0.50 | 16:05 | 5.00 |  |
|  | 18:03 | 5.15 | 0.50 | 18:04 | 4.90 |  |
| 8-Jun | 8:04 | 5.55 | 2.00 | 8:06 | 5.00 |  |
|  | 10:05 | 5.14 | 0.50 | 10:06 | 4.93 |  |
|  | 12:07 | 5.12 | 0.50 | 12:09 | 5.00 |  |
|  | 14:04 | 5.14 | 0.50 | 14:05 | 4.94 |  |
|  | 16:03 | 5.11 | 0.25 | 16:04 | 4.98 |  |
|  | 18:05 | 5.14 | 0.50 | 18:05 | 4.95 |  |

| **pH 5 Station 3 (Continued)** | | | | | | |
| --- | --- | --- | --- | --- | --- | --- |
| **Date** | **Initial Time** | **Initial pH** | **HCl added (mL)** | **Final Time** | **Final pH** | **Notes** |
| 9-Jun | 8:06 | 5.57 | 2.00 | 8:08 | 4.98 |  |
|  | 10:03 | 5.11 | 0.25 | 10:04 | 5.00 |  |
|  | 12:03 | 5.14 | 0.50 | 12:04 | 4.92 |  |
|  | 14:05 | 5.08 | 0.50 | 14:06 | 4.94 |  |
|  | 16:03 | 5.09 | 0.50 | 16:04 | 4.96 |  |
|  | 18:03 | 5.10 | 0.50 | 18:04 | 4.92 |  |
| 10-Jun | 8:07 | 5.56 | 2.00 | 8:08 | 4.90 | Overshot |
|  | 10:03 | 5.07 | 0.25 | 10:05 | 4.83 |  |
|  | 12:03 | 5.02 | <0.25 | 12:04 | 4.91 |  |
|  | 14:06 | 5.08 | 0.25 | 14:07 | 4.99 |  |
|  | 16:03 | 5.13 | 0.25 | 16:04 | 5.00 |  |
|  | 18:03 | 5.14 | 0.25 | 18:04 | 4.99 |  |
| 11-Jun | 8:06 | 5.57 | 2.50 | 8:08 | 4.99 | Overshot |
|  | 10:03 | 5.10 | 0.25 | 10:04 | 4.96 |  |
|  | 12:03 | 5.10 | 0.25 | 12:04 | 4.94 |  |
|  | 14:03 | 5.09 | <0.25 | 14:04 | 5.00 |  |
|  | 16:04 | 5.13 | 0.50 | 16:05 | 4.95 |  |
|  | 18:03 | 5.12 | 0.50 | 18:04 | 4.71 |  |
| 12-Jun | 8:05 | 5.47 | 2.00 | 8:06 | 4.98 | Overshot |
|  | 10:02 | 5.11 | 0.25 | 10:03 | 4.99 |  |
|  | 12:02 | 5.12 | 0.50 | 12:03 | 4.90 |  |
|  | 14:02 | 5.06 | 0.25 | 14:03 | 4.85 |  |
|  | 16:03 | 5.02 | <0.25 | 16:04 | 4.99 |  |
|  | 18:02 | 5.12 | 0.25 | 18:03 | 5.00 |  |
| 13-Jun | 8:03 | 5.55 | 2.00 | 8:05 | 4.96 | Precipitate on basket Overshot |
|  | 10:02 | 5.09 | 0.50 | 10:03 | 4.84 |  |
|  | 12:04 | 5.01 | <0.25 | 12:06 | 4.98 |  |
|  | 14:03 | 5.10 | 0.25 | 14:04 | 4.99 |  |
|  | 16:02 | 5.12 | 0.25 | 16:03 | 4.96 |  |
|  | 18:02 | 5.10 | 0.25 | 18:03 | 4.95 |  |
| 14-Jun | 8:08 | ~5.55 | 1.75 | 8:09 | 4.98 | Had wrong electrode plugged in at first, initial reading approximate |
|  | 10:02 | 5.08 | 0.25 | 10:03 | 4.92 |  |
|  | 12:02 | 5.05 | 0.25 | 12:03 | 4.99 |  |
|  | 14:03 | 5.11 | 0.25 | 14:04 | 5.00 |  |
|  | 16:02 | 5.11 | 0.25 | 16:03 | 4.99 |  |
|  | 8:02 | 5.10 | 0.25 | 18:03 | 4.95 |  |
| 15-Jun | 8:03 | 5.49 | 1.75 | 8:05 | 4.96 |  |
|  | 10:05 | 5.07 | 0.25 | 10:08 | 5.00 |  |
|  | 12:04 | 5.11 | 0.25 | 12:05 | 4.99 |  |
|  | 14:03 | 5.11 | 0.25 | 14:04 | 4.99 |  |
|  | 16:02 | 5.10 | 0.25 | 16:03 | 5.00 |  |
|  | 18:02 | 5.10 | 0.25 | 18:03 | 5.00 |  |
| 16-Jun | 8:04 | 5.50 | 2.25 | 8:05 | 4.99 |  |
|  | 10:02 | 5.11 | 0.25 | 10:04 | 4.95 |  |
|  | 12:03 | 5.09 | 0.25 | 12:04 | 4.99 |  |
|  | 14:02 | 5.10 | 0.25 | 14:02 | 4.98 |  |
|  | 16:02 | 5.09 | 0.25 | 16:03 | 5.00 |  |
|  | 18:02 | 5.10 | 0.25 | 18:03 | 4.98 |  |

| **pH 5 Station 3 (Continued)** | | | | | | |
| --- | --- | --- | --- | --- | --- | --- |
| **Date** | **Initial Time** | **Initial pH** | **HCl added (mL)** | **Final Time** | **Final pH** | **Notes** |
| 17-Jun | 8:03 | 5.47 | 1.75 | 8:05 | 5.00 | Checked early- Dr. apt. |
|  | 10:05 | 5.09 | 0.25 | 10:05 | 4.97 |  |
|  | 12:04 | 5.09 | 0.25 | 12:05 | 4.99 |  |
|  | 14:05 | 5.09 | 0.25 | 14:06 | 4.99 |  |
|  | 16:03 | 5.08 | 0.25 | 16:03 | 4.97 |  |
|  | 17:43 | 5.06 | <0.25 | 17:44 | 4.95 |  |
| 18-Jun | 8:06 | 5.47 | 1.75 | 8:07 | 4.97 |  |
|  | 10:02 | 5.08 | 0.25 | 10:03 | 4.96 |  |
|  | 12:02 | 5.07 | 0.25 | 12:03 | 4.99 |  |
|  | 14:03 | 5.09 | 0.25 | 14:03 | 4.96 |  |
|  | 16:01 | 5.07 | 0.25 | 16:02 | 4.98 |  |
|  | 18:02 | 5.09 | 0.25 | 18:03 | 4.96 |  |
| 19-Jun | 8:04 | 5.44 | 1.50 | 8:05 | 4.92 |  |
|  | 10:03 | 5.03 | <0.25 | 10:04 | 4.99 |  |
|  | 12:02 | 5.09 | 0.25 | 12:03 | 5.00 |  |
|  | 14:02 | 5.12 | 0.25 | 14:03 | 4.99 |  |
|  | 16:02 | 5.09 | 0.25 | 16:03 | 4.97 |  |
|  | 18:02 | 5.07 | 0.25 | 18:03 | 4.94 |  |
| 20-Jun | 8:03 | 5.42 | 1.50 | 8:07 | 4.90 | No acid added |
|  | 10:01 | 5.00 | - | 10:01 | - |  |
|  | 12:03 | 5.09 | 0.25 | 12:04 | 4.97 |  |
|  | 14:04 | 5.07 | 0.25 | 14:04 | 4.95 |  |
|  | 16:03 | 5.05 | 0.25 | 16:05 | 4.97 |  |
|  | 18:02 | 5.07 | 0.25 | 18:03 | 4.95 |  |
| 21-Jun | 8:10 | 5.39 | 1.50 | 8:11 | 4.93 |  |
|  | 10:03 | 5.02 | <0.25 | 10:04 | 4.99 |  |
|  | 12:02 | 5.08 | 0.25 | 12:03 | 5.00 |  |
|  | 14:02 | 5.09 | 0.25 | 14:03 | 4.95 |  |
|  | 16:02 | 5.05 | 0.25 | 16:02 | 4.96 |  |
|  | 18:02 | 5.07 | 0.25 | 18:03 | 4.97 |  |

| **pH 6 Station 1** | | | | | | |
| --- | --- | --- | --- | --- | --- | --- |
| **Date** | **Initial Time** | **Initial pH** | **HCl added (mL)** | **Final Time** | **Final pH** | **Notes** |
| 23-Jun | 8:00 | - | 16.00 | ~8:30 | 5.98 | Started at ~8:30  No acid added  No acid added |
|  | 10:00 | 6.22 | 2.50 | 10:03 | 5.99 |  |
|  | 12:00 | 6.00 | - | 12:00 | - |  |
|  | 14:00 | 6.05 | 0.25 | 14:02 | 5.95 |  |
|  | 16:00 | 5.97 | - | 16:00 | - |  |
|  | 18:00 | 6.05 | 1.00 | 18:02 | 5.96 |  |
| 24-Jun | 8:00 | 6.21 | 2.25 | 8:04 | 5.96 | No acid added  No acid added |
|  | 10:00 | 5.97 | - | 10:00 | - |  |
|  | 12:00 | 6.10 | 1.00 | 12:01 | 5.95 |  |
|  | 14:00 | 5.96 | - | 14:00 | - |  |
|  | 16:00 | 6.02 | <0.25 | 16:00 | 6.00 |  |
|  | 18:00 | 6.02 | <0.25 | 18:01 | 6.00 |  |
| 25-Jun | 8:03 | 6.23 | 2.25 | 8:05 | 5.98 | No acid added    No acid added |
|  | 10:00 | 5.99 | - | 10:00 | - |  |
|  | 12:00 | 6.05 | 0.50 | 12:03 | 5.99 |  |
|  | 14:00 | 6.04 | 0.50 | 14:01 | 6.00 |  |
|  | 16:00 | 6.04 | 0.50 | 16:00 | 5.95 |  |
|  | 18:00 | 5.97 | - | 18:00 | - |  |
| 26-Jun | 8:00 | 6.26 | 2.50 | 8:01 | 5.98 | No acid added   No acid added |
|  | 10:00 | 6.00 | - | 10:00 | - |  |
|  | 12:00 | 6.01 | <0.25 | 12:01 | 5.99 |  |
|  | 14:00 | 6.07 | 0.50 | 14:00 | 5.98 |  |
|  | 16:00 | 6.00 | - | 16:00 | - |  |
|  | 18:00 | 6.01 | <0.25 | 18:02 | 5.99 |  |
| 27-Jun | 10:04 | 6.31 | 3.25 | 8:01 | 5.98 | No acid added    No acid added |
|  | 10:00 | 6.02 | <0.25 | 10:00 | 5.98 |  |
|  | 12:00 | 6.00 | - | 12:00 | - |  |
|  | 14:00 | 6.04 | 0.75 | 14:01 | 5.98 |  |
|  | 16:00 | 6.04 | 0.50 | 16:00 | 5.99 |  |
|  | 18:00 | 6.00 | - | 18:00 | - |  |
| 28-Jun | 8:00 | 6.18 | 2.50 | 8:02 | 6.00 | No acid added |
|  | 10:00 | 6.02 | 0.25 | 10:06 | 6.00 |  |
|  | 12:00 | 6.01 | <0.25 | 12:02 | 5.99 |  |
|  | 14:00 | 6.00 | - | 14:00 | - |  |
|  | 16:00 | 6.03 | 0.50 | 16:02 | 6.00 |  |
|  | 18:00 | 6.01 | <0.25 | 18:01 | 5.98 |  |
| 29-Jun | 8:02 | 6.15 | 2.50 | 8:04 | 6.00 | No acid added |
|  | 10:00 | 6.03 | 0.50 | 10:03 | 5.98 |  |
|  | 12:00 | 6.02 | 0.50 | 12:01 | 5.95 |  |
|  | 14:00 | 5.97 | - | 14:03 | - |  |
|  | 16:00 | 6.06 | 1.00 | 16:02 | 5.99 |  |
|  | 18:00 | 6.02 | 0.25 | 18:01 | 5.99 |  |
| 30-Jun | 8:01 | 6.17 | 2.50 | 8:04 | 5.95 | No acid added  No acid added |
|  | 10:00 | 5.98 | - | 10:00 | - |  |
|  | 12:00 | 6.00 | - | 12:00 | - |  |
|  | 14:03 | 6.04 | 0.50 | 14:04 | 6.00 |  |
|  | 16:00 | 6.06 | 0.75 | 16:02 | 5.99 |  |
|  | 18:01 | 6.02 | 0.25 | 18:02 | 6.00 |  |

| **pH 6 Station 1 (Continued)** | | | | | | |
| --- | --- | --- | --- | --- | --- | --- |
| **Date** | **Initial Time** | **Initial pH** | **HCl added (mL)** | **Final Time** | **Final pH** | **Notes** |
| 1-Jul | 8:00 | 6.12 | 2.00 | 8:02 | 6.00 | No acid added |
|  | 10:02 | 6.04 | 0.75 | 10:04 | 5.98 |  |
|  | 12:00 | 6.01 | 0.25 | 12:01 | 5.99 |  |
|  | 14:03 | 6.00 | - | 14:03 | - |  |
|  | 16:02 | 6.02 | 0.25 | 16:03 | 6.00 |  |
|  | 18:04 | 6.03 | 0.50 | 18:05 | 6.00 |  |
| 2-Jul | 8:04 | 6.14 | 2.00 | 8:06 | 6.00 | No acid added |
|  | 10:01 | 6.04 | 0.75 | 10:02 | 5.98 |  |
|  | 12:03 | 5.99 | - | 12:03 | - |  |
|  | 14:03 | 6.01 | 0.25 | 14:04 | 5.99 |  |
|  | 16:00 | 6.04 | 0.75 | 16:00 | 5.98 |  |
|  | 18:00 | 6.02 | 0.25 | 18:00 | 5.99 |  |
| 3-Jul | 8:05 | 6.11 | 2.00 | 8:06 | 5.99 | No acid added |
|  | 10:02 | 6.00 | - | 10:02 | - |  |
|  | 12:02 | 6.03 | 0.50 | 12:02 | 6.00 |  |
|  | 14:03 | 6.02 | 0.25 | 14:04 | 5.99 |  |
|  | 16:02 | 6.01 | 0.25 | 16:03 | 5.99 |  |
|  | 18:02 | 6.02 | 0.25 | 18:03 | 6.00 |  |
| 4-Jul | 8:00 | 6.13 | 2.50 | 8:01 | 6.00 | No acid added |
|  | 10:04 | 6.02 | 0.50 | 10:05 | 5.99 |  |
|  | 12:00 | 6.04 | 0.75 | 12:00 | 5.99 |  |
|  | 14:02 | 6.00 | - | 14:02 | - |  |
|  | 16:02 | 6.02 | 0.50 | 16:03 | 6.00 |  |
|  | 18:00 | 6.02 | 0.50 | 18:00 | 6.00 |  |
| 5-Jul | 8:00 | 6.13 | 2.50 | 8:01 | 6.00 | No acid added |
|  | 10:00 | 6.03 | 0.75 | 10:01 | 5.98 |  |
|  | 12:02 | 6.00 | - | 12:02 | - |  |
|  | 14:02 | 6.02 | 0.25 | 14:03 | 5.99 |  |
|  | 16:00 | 6.04 | 0.75 | 16:01 | 6.00 |  |
|  | 18:00 | 6.02 | 0.25 | 18:00 | 5.98 |  |
| 6-Jul | 8:04 | 6.08 | 2.00 | 8:05 | 6.00 | No acid added    No acid added |
|  | 10:00 | 6.03 | 0.75 | 10:02 | 5.99 |  |
|  | 12:03 | 6.00 | - | 12:03 | - |  |
|  | 14:00 | 6.03 | 0.50 | 14:01 | 6.00 |  |
|  | 16:00 | 6.02 | 0.25 | 16:01 | 5.98 |  |
|  | 18:02 | 6.00 | - | 18:02 | - |  |
| 7-Jul | 8:00 | 6.13 | 2.50 | 8:01 | 6.00 | No acid added    No acid added |
|  | 10:01 | 6.00 | - | 10:01 | - |  |
|  | 12:00 | 6.01 | 0.50 | 12:03 | 6.00 |  |
|  | 14:00 | 6.04 | 0.50 | 14:02 | 5.99 |  |
|  | 16:00 | 6.01 | 0.50 | 16:02 | 5.95 |  |
|  | 18:02 | 5.98 | - | 18:02 | - |  |
| 8-Jul | 8:04 | 6.08 | 2.00 | 8:06 | 5.99 | No acid added  No acid added |
|  | 10:02 | 6.01 | 0.50 | 10:03 | 5.99 |  |
|  | 12:00 | 6.02 | 0.50 | 12:01 | 5.99 |  |
|  | 14:03 | 6.00 | - | 14:03 | - |  |
|  | 16:00 | 6.02 | 0.50 | 16:01 | 5.99 |  |
|  | 18:02 | 6.00 | - | 18:02 | - |  |

| **pH 6 Station 1 (Continued)** | | | | | | |
| --- | --- | --- | --- | --- | --- | --- |
| **Date** | **Initial Time** | **Initial pH** | **HCl added (mL)** | **Final Time** | **Final pH** | **Notes** |
| 9-Jul | 8:04 | 6.10 | 2.25 | 8:05 | 6.00 | No acid added |
|  | 10:00 | 6.03 | 0.50 | 10:01 | 5.98 |  |
|  | 12:02 | 6.00 | - | 12:02 | - |  |
|  | 14:03 | 6.01 | 0.50 | 14:04 | 5.99 |  |
|  | 16:09 | 6.02 | 0.50 | 16:09 | 6.00 |  |
|  | 18:01 | 6.02 | 0.50 | 18:01 | 5.99 |  |
| 10-Jul | 8:03 | 6.08 | 2.00 | 8:04 | 5.98 | No acid added No acid added  No acid added |
|  | 10:01 | 5.99 | - | 10:01 | - |  |
|  | 12:01 | 6.00 | - | 12:01 | - |  |
|  | 14:02 | 6.01 | 0.25 | 14:03 | 5.98 |  |
|  | 16:01 | 5.99 | - | 16:01 | - |  |
|  | 18:03 | 6.01 | 0.25 | 18:04 | 5.99 |  |
| 11-Jul | 8:05 | 6.07 | 1.75 | 8:06 | 5.98 | No acid added    No acid added |
|  | 10:02 | 5.99 | - | 10:02 | - |  |
|  | 12:02 | 6.01 | 0.25 | 12:04 | 5.98 |  |
|  | 14:00 | 6.02 | 0.50 | 14:00 | 6.00 |  |
|  | 16:00 | 6.01 | 0.25 | 16:01 | 5.97 |  |
|  | 18:02 | 5.99 | - | 18:02 | - |  |
| 12-Jul | 8:00 | 6.08 | 2.00 | 8:03 | 5.98 | No acid added No acid added   No acid added |
|  | 10:03 | 5.99 | - | 10:03 | - |  |
|  | 12:02 | 6.00 | - | 12:02 | - |  |
|  | 14:03 | 6.02 | 0.50 | 14:05 | 5.99 |  |
|  | 16:00 | 6.03 | 0.75 | 16:00 | 6.00 |  |
|  | 18:03 | 6.00 | - | 18:03 | - |  |
| 13-Jul | 8:07 | 6.06 | 1.50 | 8:09 | 6.00 | Initial approximate@ 10:01, wrong electrode plugged in No acid added @12:00  No acid added @14:003 |
|  | 10:01 | ~6.03 | 1.00 | 10:03 | 5.99 |  |
|  | 12:00 | 6.00 | - | 12:00 | - |  |
|  | 14:03 | 6.00 | - | 14:03 | - |  |
|  | 16:03 | 6.01 | <0.25 | 16:04 | 6.00 |  |
|  | 18:02 | 6.01 | <0.25 | 18:03 | 5.99 |  |

| **pH 6 Station 2** | | | | | | |
| --- | --- | --- | --- | --- | --- | --- |
| **Date** | **Initial Time** | **Initial pH** | **HCl added (mL)** | **Final Time** | **Final pH** | **Notes** |
| 23-Jun | 8:00 | - | 14.00 | ~8:30 | 6.00 | Started at ~8:30 |
|  | 10:03 | 6.56 | 3.50 | 10:05 | 5.92 |  |
|  | 12:02 | 6.03 | <0.25 | 12:03 | 5.97 |  |
|  | 14:03 | 6.05 | 0.25 | 14:03 | 5.99 |  |
|  | 16:01 | 6.04 | 0.25 | 16:02 | 6.00 |  |
|  | 18:02 | 6.05 | 0.25 | 18:04 | 5.99 |  |
| 24-Jun | 8:04 | 6.25 | 2.00 | 8:05 | 5.81 | Overshot No acid added  No acid added  No acid added |
|  | 10:02 | 5.87 | - | 10:02 | - |  |
|  | 12:01 | 5.94 | - | 12:01 | - |  |
|  | 14:01 | 6.00 | - | 14:01 | - |  |
|  | 16:01 | 6.05 | 0.25 | 16:02 | 5.98 |  |
|  | 18:01 | 6.06 | 0.25 | 18:02 | 5.98 |  |
| 25-Jun | 8:05 | 6.25 | 2.00 | 8:07 | 5.87 | Overshot No acid added |
|  | 10:01 | 5.96 | - | 10:01 | - |  |
|  | 12:04 | 6.01 | <0.25 | 12:04 | 5.99 |  |
|  | 14:01 | 6.04 | 0.25 | 14:02 | 6.00 |  |
|  | 16:00 | 6.05 | 0.25 | 16:01 | 6.00 |  |
|  | 18:02 | 6.03 | 0.25 | 18:03 | 5.97 |  |
| 26-Jun | 8:02 | 6.23 | 1.50 | 8:03 | 5.98 | No acid added |
|  | 10:00 | 6.00 | - | 10:00 | - |  |
|  | 12:02 | 6.05 | 0.25 | 12:03 | 5.99 |  |
|  | 14:01 | 6.04 | 0.25 | 14:01 | 5.96 |  |
|  | 16:00 | 6.03 | 0.25 | 16:01 | 5.95 |  |
|  | 18:02 | 6.01 | <0.25 | 18:03 | 5.98 |  |
| 27-Jun | 8:02 | 6.19 | 1.00 | 8:03 | 6.00 | No acid added |
|  | 10:01 | 6.10 | 0.75 | 10:01 | 5.99 |  |
|  | 12:01 | 6.05 | 0.50 | 12:02 | 5.99 |  |
|  | 14:02 | 6.04 | 0.50 | 14:03 | 5.96 |  |
|  | 16:01 | 6.02 | 0.25 | 16:01 | 5.94 |  |
|  | 18:01 | 6.00 | - | 18:01 | - |  |
| 28-Jun | 8:03 | 6.23 | 1.50 | 8:04 | 5.95 | No acid added |
|  | 10:06 | 6.03 | 0.25 | 10:07 | 5.98 |  |
|  | 12:02 | 6.03 | 0.25 | 12:03 | 5.95 |  |
|  | 14:01 | 6.01 | <0.25 | 14:02 | 5.99 |  |
|  | 16:02 | 6.03 | 0.25 | 16:03 | 5.96 |  |
|  | 18:03 | 5.99 | - | 18:03 | - |  |
| 29-Jun | 8:05 | 6.19 | 1.50 | 8:06 | 5.97 |  |
|  | 10:04 | 6.01 | 0.25 | 10:04 | 5.99 |  |
|  | 12:02 | 6.03 | 0.25 | 12:03 | 5.98 |  |
|  | 14:03 | 6.02 | 0.25 | 14:04 | 5.98 |  |
|  | 16:02 | 6.03 | 0.25 | 16:03 | 5.99 |  |
|  | 18:02 | 6.03 | 0.25 | 18:03 | 6.00 |  |
| 30-Jun | 8:05 | 6.21 | 1.75 | 8:07 | 5.98 |  |
|  | 10:01 | 6.02 | 0.25 | 10:02 | 5.98 |  |
|  | 12:01 | 6.03 | 0.25 | 12:02 | 6.00 |  |
|  | 14:01 | 6.04 | 0.50 | 14:01 | 5.98 |  |
|  | 16:02 | 6.02 | 0.25 | 16:04 | 5.99 |  |
|  | 18:02 | 6.03 | 0.25 | 18:03 | 6.00 |  |
| **pH 6 Station 2 (Continued)** | | | | | | |
| **Date** | **Initial Time** | **Initial pH** | **HCl added (mL)** | **Final Time** | **Final pH** | **Notes** |
| 1-Jul | 8:02 | 6.22 | 1.75 | 8:04 | 5.99 | No acid added |
|  | 10:04 | 6.03 | 0.25 | 10:05 | 5.98 |  |
|  | 12:02 | 6.03 | 0.25 | 12:02 | 6.00 |  |
|  | 14:01 | 6.04 | 0.50 | 14:01 | 5.95 |  |
|  | 16:01 | 6.00 | - | 16:01 | - |  |
|  | 18:01 | 6.04 | 0.25 | 18:02 | 5.99 |  |
| 2-Jul | 8:01 | 6.21 | 1.75 | 8:02 | 5.98 |  |
|  | 10:02 | 6.05 | 0.50 | 10:04 | 5.98 |  |
|  | 12:01 | 6.03 | 0.25 | 12:01 | 5.99 |  |
|  | 14:01 | 6.04 | 0.50 | 14:02 | 5.97 |  |
|  | 16:01 | 6.02 | 0.25 | 16:01 | 5.98 |  |
|  | 18:01 | 6.02 | 0.25 | 18:01 | 6.00 |  |
| 3-Jul | 8:01 | 6.20 | 1.75 | 8:02 | 5.97 |  |
|  | 10:00 | 6.03 | 0.25 | 10:01 | 5.98 |  |
|  | 12:00 | 6.03 | 0.25 | 12:00 | 6.00 |  |
|  | 14:01 | 6.03 | 0.25 | 14:02 | 5.99 |  |
|  | 16:00 | 6.03 | 0.25 | 16:01 | 6.00 |  |
|  | 18:00 | 6.02 | 0.25 | 18:00 | 5.99 |  |
| 4-Jul | 8:01 | 6.20 | 2.00 | 8:02 | 6.00 |  |
|  | 10:01 | 6.03 | 0.25 | 10:02 | 5.99 |  |
|  | 12:01 | 6.03 | 0.25 | 12:01 | 5.99 |  |
|  | 14:00 | 6.03 | 0.25 | 14:00 | 6.00 |  |
|  | 16:00 | 6.04 | 0.50 | 16:01 | 5.98 |  |
|  | 18:01 | 6.02 | 0.25 | 18:01 | 5.97 |  |
| 5-Jul | 8:02 | 6.19 | 2.00 | 8:03 | 5.98 |  |
|  | 10:02 | 6.02 | 0.25 | 10:02 | 6.00 |  |
|  | 12:00 | 6.04 | 0.50 | 12:01 | 5.97 |  |
|  | 14:00 | 6.02 | 0.25 | 14:01 | 5.99 |  |
|  | 16:01 | 6.02 | 0.25 | 16:02 | 6.00 |  |
|  | 18:01 | 6.03 | 0.50 | 18:01 | 5.99 |  |
| 6-Jul | 8:00 | 6.19 | 2.00 | 8:01 | 5.98 |  |
|  | 10:06 | 6.01 | 0.25 | 10:07 | 5.99 |  |
|  | 12:00 | 6.01 | 0.25 | 12:01 | 5.99 |  |
|  | 14:01 | 6.02 | 0.25 | 14:02 | 6.00 |  |
|  | 16:01 | 6.02 | 0.25 | 16:02 | 6.00 |  |
|  | 18:00 | 6.03 | 0.50 | 18:01 | 6.00 |  |
| 7-Jul | 8:01 | 6.18 | 2.00 | 8:02 | 5.95 | No acid added   No acid added |
|  | 10:00 | 5.98 | - | 10:00 | - |  |
|  | 12:05 | 6.02 | 0.25 | 12:06 | 5.99 |  |
|  | 14:03 | 6.02 | 0.50 | 14:03 | 5.98 |  |
|  | 16:02 | 6.00 | - | 16:03 | - |  |
|  | 18:00 | 6.04 | 0.50 | 18:01 | 5.97 |  |
| 8-Jul | 8:00 | 6.18 | 2.00 | 8:01 | 5.94 | No acid added |
|  | 10:00 | 5.99 | - | 10:00 | - |  |
|  | 12:02 | 6.02 | 0.25 | 12:03 | 5.99 |  |
|  | 14:00 | 6.02 | 0.25 | 14:00 | 5.99 |  |
|  | 16:01 | 6.02 | 0.25 | 16:02 | 6.00 |  |
|  | 18:00 | 6.03 | 0.25 | 18:01 | 6.00 |  |
| **pH 6 Station 2 (Continued)** | | | | | | |
| **Date** | **Initial Time** | **Initial pH** | **HCl added (mL)** | **Final Time** | **Final pH** | **Notes** |
| 9-Jul | 8:00 | 6.21 | 2.00 | 8:02 | 5.99 |  |
|  | 10:01 | 6.02 | 0.25 | 10:02 | 6.00 |  |
|  | 12:00 | 6.03 | 0.25 | 12:01 | 6.00 |  |
|  | 14:00 | 6.02 | 0.25 | 14:01 | 6.00 |  |
|  | 16:11 | 6.03 | 0.25 | 16:12 | 5.98 |  |
|  | 18:02 | 6.02 | 0.25 | 18:02 | 5.96 |  |
| 10-Jul | 8:00 | 6.19 | 1.75 | 8:00 | 5.99 |  |
|  | 10:00 | 6.01 | 0.25 | 10:00 | 5.98 |  |
|  | 12:00 | 6.01 | 0.25 | 12:00 | 5.98 |  |
|  | 14:00 | 6.03 | 0.25 | 14:00 | 5.99 |  |
|  | 16:00 | 6.03 | 0.25 | 16:00 | 6.00 |  |
|  | 18:00 | 6.03 | 0.25 | 18:00 | 5.99 |  |
| 11-Jul | 8:00 | 6.20 | 1.75 | 8:02 | 5.98 | No acid added |
|  | 10:00 | 6.01 | 0.25 | 10:00 | 5.99 |  |
|  | 12:00 | 6.02 | 0.50 | 12:01 | 5.99 |  |
|  | 14:01 | 6.01 | 0.25 | 14:01 | 5.98 |  |
|  | 16:01 | 6.00 | - | 16:01 | - |  |
|  | 18:00 | 6.03 | 0.50 | 18:00 | 6.00 |  |
| 12-Jul | 8:04 | 6.21 | 2.00 | 8:06 | 6.00 | No acid added |
|  | 10:00 | 6.01 | 0.25 | 10:01 | 5.99 |  |
|  | 12:00 | 6.02 | 0.50 | 12:01 | 5.98 |  |
|  | 14:01 | 6.01 | 0.25 | 14:01 | 5.98 |  |
|  | 16:01 | 6.00 | - | 16:01 | - |  |
|  | 18:00 | 6.03 | 0.50 | 18:00 | 5.98 |  |
| 13-Jul | 8:02 | 6.19 | 1.50 | 8:04 | 6.00 | No acid added |
|  | 10:01 | 6.03 | 0.50 | 10:05 | 5.98 |  |
|  | 12:01 | 6.01 | 0.25 | 12:01 | 5.99 |  |
|  | 14:00 | 6.01 | 0.25 | 14:01 | 5.98 |  |
|  | 16:00 | 6.00 | - | 16:00 | - |  |
|  | 18:00 | 6.04 | 0.50 | 18:00 | 5.98 |  |

| **pH 6 Station 3** | | | | | | |
| --- | --- | --- | --- | --- | --- | --- |
| **Date** | **Initial Time** | **Initial pH** | **HCl added (mL)** | **Final Time** | **Final pH** | **Notes** |
| 23-Jun | 8:00 | - | 13.00 | ~8:30 | 6.00 | Started at ~8:30     No acid added |
|  | 10:06 | 6.48 | 4.50 | 10:08 | 5.97 |  |
|  | 12:03 | 6.01 | <0.25 | 12:05 | 5.99 |  |
|  | 14:04 | 6.02 | 0.25 | 14:05 | 5.99 |  |
|  | 16:03 | 6.02 | 0.25 | 16:03 | 5.97 |  |
|  | 18:04 | 6.00 | - | 18:04 | - |  |
| 24-Jun | 8:06 | 6.18 | 2.00 | 8:08 | 5.95 | No acid added |
|  | 10:06 | 5.97 | - | 10:03 | - |  |
|  | 12:02 | 6.01 | <0.25 | 12:03 | 5.99 |  |
|  | 14:02 | 6.01 | <0.25 | 14:03 | 5.99 |  |
|  | 16:02 | 6.02 | <0.25 | 16:03 | 6.00 |  |
|  | 18:03 | 6.02 | <0.25 | 18:04 | 6.00 |  |
| 25-Jun | 8:07 | 6.19 | 2.00 | 8:08 | 5.93 | No acid added  No acid added |
|  | 10:02 | 5.96 | - | 10:02 | - |  |
|  | 12:05 | 6.00 | - | 12:05 | - |  |
|  | 14:02 | 6.03 | 0.25 | 14:03 | 6.00 |  |
|  | 16:01 | 6.03 | 0.25 | 16:02 | 6.00 |  |
|  | 18:04 | 6.03 | 0.25 | 18:05 | 5.99 |  |
| 26-Jun | 8:03 | 6.16 | 1.50 | 8:04 | 6.00 | No acid added Last pH digit not recorded at 18:00, likely higher, ~5.99 |
|  | 10:01 | 6.02 | 0.25 | 10:03 | 6.00 |  |
|  | 12:03 | 6.05 | 0.25 | 12:04 | 5.99 |  |
|  | 14:02 | 6.00 | - | 14:02 | - |  |
|  | 16:01 | 6.04 | 0.25 | 16:02 | 6.00 |  |
|  | 18:03 | 6.03 | 0.25 | 18:05 | 5.90 |  |
| 27-Jun | 8:04 | 6.17 | 1.75 | 8:05 | 6.00 | No acid added |
|  | 10:02 | 6.03 | 0.25 | 10:02 | 6.00 |  |
|  | 12:03 | 6.04 | 0.25 | 12:04 | 5.98 |  |
|  | 14:04 | 6.01 | <0.25 | 14:05 | 5.98 |  |
|  | 16:02 | 6.00 | - | 16:02 | - |  |
|  | 18:02 | 6.03 | 0.25 | 18:03 | 5.99 |  |
| 28-Jun | 8:05 | 6.17 | 2.00 | 8:06 | 6.00 |  |
|  | 10:08 | 6.04 | 0.25 | 10:04 | 5.99 |  |
|  | 12:04 | 6.03 | 0.25 | 12:04 | 5.99 |  |
|  | 14:03 | 6.02 | <0.25 | 14:04 | 6.00 |  |
|  | 16:03 | 6.03 | 0.25 | 16:04 | 6.00 |  |
|  | 18:04 | 6.03 | 0.25 | 18:05 | 6.00 |  |
| 29-Jun | 8:07 | 6.16 | 1.75 | 8:08 | 5.99 |  |
|  | 10:05 | 6.03 | 0.25 | 10:06 | 6.00 |  |
|  | 12:03 | 6.02 | 0.50 | 12:04 | 5.99 |  |
|  | 14:04 | 6.01 | <0.25 | 14:05 | 5.99 |  |
|  | 16:03 | 6.01 | <0.26 | 16:04 | 5.98 |  |
|  | 18:03 | 6.01 | <0.27 | 18:04 | 5.99 |  |
| 30-Jun | 8:07 | 6.14 | 1.75 | 8:08 | 5.99 | No acid added  No acid added |
|  | 10:02 | 6.00 | - | 10:02 | - |  |
|  | 12:02 | 6.03 | 0.25 | 12:03 | 6.00 |  |
|  | 14:02 | 6.04 | 0.50 | 14:03 | 5.99 |  |
|  | 16:04 | 6.00 | - | 16:04 | - |  |
|  | 18:04 | 6.01 | <0.25 | 18:05 | 6.00 |  |
| **pH 6 Station 3 (Continued)** | | | | | | |
| **Date** | **Initial Time** | **Initial pH** | **HCl added (mL)** | **Final Time** | **Final pH** | **Notes** |
| 1-Jul | 8:04 | 6.17 | 2.00 | 8:05 | 5.99 |  |
|  | 10:06 | 6.01 | <0.25 | 10:07 | 6.00 |  |
|  | 12:03 | 6.03 | 0.25 | 12:04 | 5.99 |  |
|  | 14:02 | 6.02 | 0.25 | 14:02 | 6.00 |  |
|  | 16:01 | 6.02 | 0.25 | 16:02 | 5.99 |  |
|  | 18:03 | 6.01 | 0.25 | 18:04 | 5.98 |  |
| 2-Jul | 8:03 | 6.13 | 1.75 | 8:04 | 5.99 |  |
|  | 10:06 | 6.01 | <0.25 | 10:06 | 6.00 |  |
|  | 12:02 | 6.01 | <0.26 | 12:02 | 6.00 |  |
|  | 14:02 | 6.02 | 0.25 | 14:03 | 6.00 |  |
|  | 16:02 | 6.03 | 0.50 | 16:02 | 6.00 |  |
|  | 18:02 | 6.01 | <0.25 | 18:02 | 5.99 |  |
| 3-Jul | 8:03 | 6.12 | 1.75 | 8:04 | 5.99 | No acid added |
|  | 10:01 | 6.01 | 0.25 | 10:02 | 5.99 |  |
|  | 12:03 | 6.02 | 0.25 | 12:03 | 5.99 |  |
|  | 14:02 | 6.01 | 0.25 | 14:03 | 5.98 |  |
|  | 16:01 | 6.01 | 0.25 | 16:02 | 5.99 |  |
|  | 18:01 | 6.00 | - | 18:01 | - |  |
| 4-Jul | 8:03 | 6.12 | 1.75 | 8:04 | 5.99 | No acid added    No acid added |
|  | 10:03 | 6.01 | 0.25 | 10:04 | 5.99 |  |
|  | 12:02 | 6.00 | - | 12:02 | - |  |
|  | 14:01 | 6.03 | 0.50 | 14:01 | 5.98 |  |
|  | 16:01 | 6.00 | - | 16:01 | - |  |
|  | 18:02 | 6.02 | 0.25 | 18:03 | 5.99 |  |
| 5-Jul | 8:04 | 6.12 | 1.75 | 8:05 | 6.00 |  |
|  | 10:03 | 6.01 | <0.25 | 10:03 | 6.00 |  |
|  | 12:01 | 6.02 | 0.25 | 12:02 | 6.00 |  |
|  | 14:01 | 6.02 | 0.25 | 14:02 | 5.99 |  |
|  | 16:02 | 6.02 | 0.25 | 16:03 | 6.00 |  |
|  | 18:02 | 6.02 | 0.25 | 18:02 | 5.99 |  |
| 6-Jul | 8:02 | 6.10 | 1.50 | 8:03 | 5.99 | No acid added  No acid added  No acid added |
|  | 10:09 | 6.00 | - | 10:09 | - |  |
|  | 12:01 | 6.02 | 0.50 | 12:02 | 5.99 |  |
|  | 14:02 | 6.00 | - | 14:02 | - |  |
|  | 16:02 | 6.02 | 0.25 | 16:03 | 5.99 |  |
|  | 18:02 | 6.00 | - | 18:02 | - |  |
| 7-Jul | 8:03 | 6.11 | 1.75 | 8:04 | 6.00 | No acid added  No acid added  No acid added |
|  | 10:03 | 6.00 | - | 10:03 | - |  |
|  | 12:01 | 6.02 | 0.50 | 12:08 | 5.99 |  |
|  | 14:04 | 6.00 | - | 14:04 | - |  |
|  | 16:04 | 6.01 | 0.25 | 16:05 | 5.99 |  |
|  | 18:01 | 6.00 | - | 18:01 | - |  |
| 8-Jul | 8:02 | 6.10 | 1.75 | 8:03 | 5.98 | No acid added   No acid added |
|  | 10:01 | 5.99 | - | 10:01 | - |  |
|  | 12:03 | 6.01 | 0.25 | 12:04 | 5.99 |  |
|  | 14:02 | 6.00 | - | 14:02 | - |  |
|  | 16:02 | 6.01 | <0.25 | 16:03 | 6.00 |  |
|  | 18:01 | 6.02 | 0.25 | 18:02 | 5.99 |  |
| **pH 6 Station 3 (Continued)** | | | | | | |
| **Date** | **Initial Time** | **Initial pH** | **HCl added (mL)** | **Final Time** | **Final pH** | **Notes** |
| 9-Jul | 8:02 | 6.09 | 1.75 | 8:03 | 5.98 | No acid added  No acid added |
|  | 10:02 | 6.00 | - | 10:02 | - |  |
|  | 12:01 | 6.00 | - | 12:01 | - |  |
|  | 14:01 | 6.01 | <0.25 | 14:02 | 6.00 |  |
|  | 16:13 | 6.02 | 0.25 | 16:14 | 5.99 |  |
|  | 18:03 | 6.02 | 0.25 | 18:04 | 5.98 |  |
| 10-Jul | 8:01 | 6.08 | 1.25 | 8:02 | 6.00 | No acid added    No acid added |
|  | 10:01 | 6.00 | - | 10:01 | - |  |
|  | 12:00 | 6.01 | 0.25 | 12:01 | 6.00 |  |
|  | 14:01 | 6.01 | 0.25 | 14:01 | 5.99 |  |
|  | 16:01 | 6.00 | - | 16:01 | - |  |
|  | 18:01 | 6.01 | 0.25 | 18:01 | 5.98 |  |
| 11-Jul | 8:03 | 6.07 | 1.25 | 8:04 | 5.99 | No acid added   No acid added  No acid added |
|  | 10:01 | 6.00 | - | 10:01 | - |  |
|  | 12:01 | 6.01 | 0.25 | 12:02 | 5.99 |  |
|  | 14:02 | 6.01 | 0.25 | 14:03 | 5.98 |  |
|  | 16:02 | 5.99 | - | 16:02 | - |  |
|  | 18:02 | 6.00 | - | 18:01 | - |  |
| 12-Jul | 8:06 | 6.09 | 1.75 | 8:07 | 5.98 | No acid added  No acid added |
|  | 10:02 | 5.99 | - | 10:02 | - |  |
|  | 12:02 | 6.00 | - | 12:02 | - |  |
|  | 14:02 | 6.01 | 0.25 | 14:03 | 6.00 |  |
|  | 16:02 | 6.01 | 0.25 | 16:03 | 5.99 |  |
|  | 18:01 | 6.01 | 0.25 | 18:02 | 5.99 |  |
| 13-Jul | 8:05 | 6.08 | 1.25 | 8:06 | 6.00 | No acid added  No acid added |
|  | 10:06 | 6.00 | - | 10:06 | - |  |
|  | 12:02 | 6.02 | 0.25 | 12:02 | 5.99 |  |
|  | 14:02 | 6.00 | - | 14:02 | - |  |
|  | 16:01 | 6.01 | <0.25 | 16:02 | 6.00 |  |
|  | 18:01 | 6.01 | 0.25 | 18:01 | 5.99 |  |
