# Supplementary material for: Experimental dissolution of fossil bone under variable pH conditions
Source: PLoS One. 2022 Oct 13;17(10):e0274084. doi: 10.1371/journal.pone.0274084 (PMC9560490; doi:10.1371/journal.pone.0274084)
Supplement: S2 Appendix — (DOCX) [file pone.0274084.s002.docx]

| **pH 4 ICP-MS Data** | | | | | | | | | | |
| --- | --- | --- | --- | --- | --- | --- | --- | --- | --- | --- |
|  | **Na** | | **Mg** | | **Al** | | **K** | | **Mn** | |
|  | Conc.(ppm) | RSD | Conc.(ppm) | RSD | Conc.(ppm) | RSD | Conc.(ppm) | RSD | Conc.(ppm) | RSD |
| **pH 4 Blank Initial** | 7.41 | 0.11 | 18.550 | 0.348 | 0.14 | 1.96 | 4.239 | 0.329 | <0.010 | N/A |
| **pH 4 Blank Final** | 8.34 | 0.29 | 19.805 | 0.900 | 0.11 | 5.70 | 4.298 | 0.474 | <0.001 | N/A |
| **pH 4 F1 initial (aqueous)** | 7.44 | 1.02 | 18.64 | 0.47 | 0.15 | 1.09 | 3.02 | 1.10 | <0.010 | N/A |
| **pH 4 F2 initial (aqueous)** | 7.40 | 0.82 | 18.52 | 0.38 | 0.15 | 8.68 | 3.43 | 0.63 | <0.010 | N/A |
| **pH 4 F3 initial (aqueous)** | 7.43 | 0.90 | 18.57 | 0.92 | 0.13 | 7.23 | 3.49 | 0.79 | <0.010 | N/A |
| **pH 4 F1 final (aqueous)** | 9.25 | 0.33 | 21.204 | 0.569 | 1.82 | 0.89 | 48.592 | 0.153 | 1.69 | 0.44 |
| **pH 4 F2 final (aqueous)** | 9.18 | 0.57 | 19.099 | 1.083 | 1.91 | 1.82 | 151.737 | 0.415 | 1.23 | 0.62 |
| **pH 4 F3 final (aqueous)** | 8.99 | 1.96 | 18.740 | 1.305 | 1.55 | 2.08 | 93.147 | 0.934 | 1.19 | 1.20 |
| **F1 initial (fossil)** | 3188.77 | 1.08 | 223.75 | 1.09 | 1343.04 | 0.41 | 157.24 | 0.66 | 281.32 | 0.35 |
| **F2 initial (fossil)** | 2321.37 | 1.31 | 91.06 | 0.81 | 5085.45 | 1.06 | 39.07 | 3.35 | 69.89 | 0.99 |
| **F3 initial (fossil)** | 2707.66 | 1.58 | 51.12 | 1.19 | 564.41 | 1.04 | 16.29 | 0.92 | 129.87 | 0.74 |
| **pH 4 F1 final (fossil)** | 3935.13 | 0.42 | 1682.970 | 0.502 | 10821.40 | 1.07 | 920.422 | 1.086 | 1225.32 | 0.25 |
| **pH 4 F2 final (fossil)** | 3646.55 | 1.21 | 1367.206 | 0.604 | 15975.43 | 1.14 | 921.023 | 1.002 | 1040.52 | 0.18 |
| **pH 4 F3 final (fossil)** | 4379.18 | 1.24 | 1274.962 | 0.668 | 3841.60 | 1.45 | 352.489 | 4.728 | 1555.91 | 0.58 |

| **pH 4 ICP-MS Data (Continued)** | | | | | | | | | | |
| --- | --- | --- | --- | --- | --- | --- | --- | --- | --- | --- |
|  | **Fe** | | **Sr** | | **Ba** | | **Ca** | | **P** | |
|  | Conc.(ppm) | RSD | Conc.(ppm) | RSD | Conc.(ppm) | RSD | Conc.(ppm) | RSD | Conc.(ppm) | RSD |
| **pH 4 Blank Initial** | 0.007 | 8.033 | 0.11 | 1.89 | 0.090 | 2.029 | 35.973 | 0.613 | <0.07 | N/A |
| **pH 4 Blank Final** | <0.007 | N/A | 0.12 | 1.54 | 0.094 | 1.231 | 37.203 | 1.407 | <0.07 | N/A |
| **pH 4 F1 initial (aqueous)** | 0.01 | 87.75 | 0.11 | 0.51 | 0.09 | 0.26 | 37.530 | 1.516 | <0.07 | N/A |
| **pH 4 F2 initial (aqueous)** | 0.01 | 10.49 | 0.11 | 0.72 | 0.09 | 1.52 | 36.469 | 1.203 | <0.07 | N/A |
| **pH 4 F3 initial (aqueous)** | 0.01 | 7.43 | 0.11 | 0.63 | 0.09 | 1.19 | 36.799 | 1.915 | <0.05 | N/A |
| **pH 4 F1 final (aqueous)** | 2.082 | 0.295 | 2.70 | 0.67 | 0.786 | 1.139 | 352.872 | 0.948 | 10.931 | 0.471 |
| **pH 4 F2 final (aqueous)** | 1.710 | 0.707 | 2.21 | 1.09 | 0.493 | 1.251 | 289.074 | 1.159 | 14.433 | 1.348 |
| **pH 4 F3 final (aqueous)** | 1.553 | 1.397 | 2.02 | 0.79 | 0.436 | 0.396 | 235.176 | 1.496 | 18.787 | 0.928 |
| **F1 initial (fossil)** | 5185.17 | 0.41 | 841.87 | 0.29 | 1294.90 | 0.50 | 41.957 | 1.381 | 11.813 | 1.501 |
| **F2 initial (fossil)** | 2793.69 | 0.44 | 808.62 | 0.55 | 1154.93 | 0.98 | 32.393 | 0.710 | 13.890 | 1.424 |
| **F3 initial (fossil)** | 788.55 | 0.94 | 322.97 | 0.74 | 4908.43 | 0.70 | 26.395 | 0.687 | 10.226 | 0.686 |
| **pH 4 F1 final (fossil)** | 20283.920 | 1.049 | 2775.19 | 0.43 | 3624.153 | 0.722 | 32.663 | 1.150 | 10.924 | 1.730 |
| **pH 4 F2 final (fossil)** | 15121.564 | 1.143 | 3012.59 | 1.06 | 4795.890 | 1.281 | 22.566 | 1.175 | 7.824 | 0.394 |
| **pH 4 F3 final (fossil)** | 9052.393 | 0.939 | 3516.42 | 1.21 | 4600.741 | 1.020 | 38.791 | 1.290 | 13.478 | 1.076 |

| **pH 5 ICP-MS Data** | | | | | | | | | | |
| --- | --- | --- | --- | --- | --- | --- | --- | --- | --- | --- |
|  | **Na** | | **Mg** | | **Al** | | **K** | | **Mn** | |
|  | Conc.(ppm) | RSD | Conc.(ppm) | RSD | Conc.(ppm) | RSD | Conc.(ppm) | RSD | Conc.(ppm) | RSD |
| **pH 5 Blank Initial** | 8.10 | 0.56 | 19.277 | 0.503 | 0.14 | 3.61 | 2.964 | 0.626 | <0.001 | N/A |
| **pH 5 Blank Final** | 7.10 | 0.98 | 17.207 | 1.116 | 0.13 | 0.36 | 2.675 | 1.413 | <0.01 | N/A |
| **pH 5 F1 initial (aqueous)** | 8.12 | 1.21 | 19.241 | 1.057 | 0.15 | 2.07 | 3.533 | 0.236 | <0.001 | N/A |
| **pH 5 F2 initial (aqueous)** | 8.09 | 0.44 | 19.367 | 0.528 | 0.15 | 6.59 | 6.241 | 0.529 | 0.00 | 21.92 |
| **pH 5 F3 initial (aqueous)** | 8.07 | 0.05 | 19.143 | 0.710 | 0.13 | 7.53 | 4.847 | 1.199 | 0.00 | 11.19 |
| **pH 5 F1 final (aqueous)** | 6.73 | 1.12 | 18.130 | 1.221 | 0.16 | 2.06 | 62.204 | 0.999 | 1.49 | 0.72 |
| **pH 5 F2 final (aqueous)** | 7.73 | 0.79 | 17.524 | 0.219 | 0.11 | 2.26 | 114.183 | 0.987 | 0.55 | 0.85 |
| **pH 5 F3 final (aqueous)** | 7.06 | 0.90 | 17.340 | 1.463 | 0.13 | 1.21 | 80.052 | 1.170 | 1.38 | 1.01 |
| **F1 initial (fossil)** | 3188.77 | 1.08 | 223.75 | 1.09 | 1343.04 | 0.41 | 157.24 | 0.66 | 281.32 | 0.35 |
| **F2 initial (fossil)** | 2321.37 | 1.31 | 91.06 | 0.81 | 5085.45 | 1.06 | 39.07 | 3.35 | 69.89 | 0.99 |
| **F3 initial (fossil)** | 2707.66 | 1.58 | 51.12 | 1.19 | 564.41 | 1.04 | 16.29 | 0.92 | 129.87 | 0.74 |
| **pH 5 F1 final (fossil)** | 1614.58 | 3.00 | 779.880 | 1.899 | 5146.79 | 2.86 | 515.399 | 4.668 | 578.39 | 1.32 |
| **pH 5 F2 final (fossil)** | 4117.23 | 1.09 | 1733.291 | 1.265 | 20249.38 | 0.28 | 1192.892 | 1.173 | 1313.37 | 0.62 |
| **pH 5 F3 final (fossil)** | 4670.16 | 0.82 | 1451.465 | 1.409 | 4413.74 | 2.11 | 437.981 | 2.704 | 1807.38 | 0.60 |

| **pH 5 ICP-MS Data (Continued)** | | | | | | | | | | |
| --- | --- | --- | --- | --- | --- | --- | --- | --- | --- | --- |
|  | **Fe** | | **Sr** | | **Ba** | | **Ca** | | **P** | |
|  | Conc.(ppm) | RSD | Conc.(ppm) | RSD | Conc.(ppm) | RSD | Conc.(ppm) | RSD | Conc.(ppm) | RSD |
| **pH 5 Blank Initial** | <0.007 | 23.522 | 0.12 | 0.60 | 0.091 | 1.237 | 16.768 | 1.577 | 6.686 | 2.263 |
| **pH 5 Blank Final** | <0.01 | N/A | 0.11 | 1.63 | 0.086 | 2.369 | 36.072 | 1.095 | <0.05 | N/A |
| **pH 5 F1 initial (aqueous)** | <0.007 | 11.786 | 0.11 | 0.52 | 0.091 | 1.791 | 36.830 | 0.386 | <0.07 | 572.779 |
| **pH 5 F2 initial (aqueous)** | 0.009 | 13.066 | 0.13 | 2.55 | 0.101 | 1.548 | 37.505 | 0.501 | 0.086 | 8.496 |
| **pH 5 F3 initial (aqueous)** | 0.010 | 2.775 | 0.13 | 0.84 | 0.098 | 1.593 | 37.988 | 1.315 | 0.096 | 17.148 |
| **pH 5 F1 final (aqueous)** | 0.685 | 0.520 | 2.22 | 1.22 | 0.388 | 2.591 | 385.902 | 1.123 | <0.05 | N/A |
| **pH 5 F2 final (aqueous)** | 0.163 | 1.328 | 1.18 | 0.84 | 0.210 | 1.657 | 166.550 | 0.784 | 0.668 | 4.234 |
| **pH 5 F3 final (aqueous)** | 0.861 | 0.802 | 1.84 | 0.40 | 0.202 | 1.008 | 259.942 | 1.373 | 0.149 | 16.819 |
| **F1 initial (fossil)** | 5185.17 | 0.41 | 841.87 | 0.29 | 1294.90 | 0.50 | 41.957 | 1.381 | 11.813 | 1.501 |
| **F2 initial (fossil)** | 2793.69 | 0.44 | 808.62 | 0.55 | 1154.93 | 0.98 | 32.393 | 0.710 | 13.890 | 1.424 |
| **F3 initial (fossil)** | 788.55 | 0.94 | 322.97 | 0.74 | 4908.43 | 0.70 | 26.395 | 0.687 | 10.226 | 0.686 |
| **pH 5 F1 final (fossil)** | 9558.714 | 1.845 | 1327.94 | 1.12 | 1707.298 | 1.140 | 38.391 | 0.401 | 8.662 | 0.260 |
| **pH 5 F2 final (fossil)** | 19027.764 | 0.680 | 3848.82 | 1.16 | 6220.693 | 0.970 | 35.831 | 0.350 | 12.301 | 1.585 |
| **pH 5 F3 final (fossil)** | 10459.350 | 0.763 | 4169.41 | 1.09 | 5514.097 | 0.156 | 39.114 | 0.670 | 10.957 | 2.066 |

| **pH 6 ICP-MS Data** | | | | | | | | | | |
| --- | --- | --- | --- | --- | --- | --- | --- | --- | --- | --- |
|  | **Na** | | **Mg** | | **Al** | | **K** | | **Mn** | |
|  | Conc.(ppm) | RSD | Conc.(ppm) | RSD | Conc.(ppm) | RSD | Conc.(ppm) | RSD | Conc.(ppm) | RSD |
| **pH 6 Blank Initial** | 7.373 | 0.596 | 17.438 | 0.235 | 0.135 | 4.479 | 2.385 | 0.274 | <0.01 | N/A |
| **pH 6 Blank Final** | 8.201 | 0.745 | 19.760 | 0.240 | 0.071 | 3.196 | 2.602 | 0.398 | 0.002 | 13.289 |
| **pH 6 F1 initial (aqueous)** | 7.11 | 1.04 | 17.644 | 0.776 | 0.14 | 2.53 | 3.261 | 1.120 | <0.01 | N/A |
| **pH 6 F2 initial (aqueous)** | 6.91 | 1.86 | 17.153 | 1.669 | 0.14 | 2.72 | 2.547 | 1.442 | <0.01 | N/A |
| **pH 6 F3 initial (aqueous)** | 7.04 | 0.20 | 17.358 | 0.715 | 0.11 | 2.63 | 2.642 | 0.317 | <0.01 | N/A |
| **pH 6 F1 final (aqueous)** | 8.43 | 0.59 | 21.455 | 0.478 | 0.03 | 12.14 | 40.691 | 0.732 | 1.16 | 0.61 |
| **pH 6 F2 final (aqueous)** | 8.59 | 0.52 | 20.285 | 0.443 | 0.03 | 4.46 | 182.965 | 0.369 | 0.73 | 0.09 |
| **pH 6 F3 final (aqueous)** | 7.95 | 0.07 | 18.883 | 0.126 | 0.02 | 12.99 | 125.620 | 0.485 | 1.21 | 0.75 |
| **F1 initial (fossil)** | 3188.77 | 1.08 | 223.75 | 1.09 | 1343.04 | 0.41 | 157.24 | 0.66 | 281.32 | 0.35 |
| **F2 initial (fossil)** | 2321.37 | 1.31 | 91.06 | 0.81 | 5085.45 | 1.06 | 39.07 | 3.35 | 69.89 | 0.99 |
| **F3 initial (fossil)** | 2707.66 | 1.58 | 51.12 | 1.19 | 564.41 | 1.04 | 16.29 | 0.92 | 129.87 | 0.74 |
| **pH 6 F1 final (fossil)** | 3378.76 | 0.31 | 2132.979 | 0.812 | 372.24 | 2.39 | 167.328 | 11.619 | 1141.35 | 0.37 |
| **pH 6 F2 final (fossil)** | 2380.17 | 1.01 | 2615.843 | 0.902 | 217.96 | 3.26 | 152.369 | 13.709 | 1092.17 | 0.61 |
| **pH 6 F3 final (fossil)** | 3145.36 | 0.35 | 1295.695 | 0.526 | 296.87 | 2.45 | 146.201 | 7.450 | 1517.19 | 0.89 |

| **pH 6 ICP-MS Data (Continued)** | | | | | | | | | | |
| --- | --- | --- | --- | --- | --- | --- | --- | --- | --- | --- |
|  | **Fe** | | **Sr** | | **Ba** | | **Ca** | | **P** | |
|  | Conc.(ppm) | RSD | Conc.(ppm) | RSD | Conc.(ppm) | RSD | Conc.(ppm) | RSD | Conc.(ppm) | RSD |
| **pH 6 Blank Initial** | 0.003 | 36.989 | 0.109 | 0.821 | 0.085 | 0.502 | 36.806 | 0.373 | <0.05 | N/A |
| **pH 6 Blank Final** | <0.13 | N/A | 0.124 | 1.475 | 0.088 | 1.211 | 44.384 | 0.681 | <0.030 | N/A |
| **pH 6 F1 initial (aqueous)** | 0.001 | 8.643 | 0.11 | 0.46 | 0.086 | 1.336 | 36.238 | 0.502 | <0.05 | N/A |
| **pH 6 F2 initial (aqueous)** | 0.002 | 11.339 | 0.11 | 0.53 | 0.085 | 0.216 | 36.503 | 1.812 | <0.05 | N/A |
| **pH 6 F3 initial (aqueous)** | <0.01 | 4.813 | 0.11 | 0.40 | 0.085 | 1.989 | 36.337 | 1.404 | <0.05 | N/A |
| **pH 6 F1 final (aqueous)** | <0.13 | N/A | 1.85 | 0.16 | 0.291 | 0.539 | 317.180 | 0.392 | <0.030 | 114.680 |
| **pH 6 F2 final (aqueous)** | <0.13 | N/A | 1.61 | 0.64 | 0.236 | 0.645 | 229.564 | 0.641 | 0.060 | 39.463 |
| **pH 6 F3 final (aqueous)** | <0.13 | N/A | 1.48 | 0.05 | 0.213 | 1.646 | 217.989 | 0.439 | <0.030 | 148.712 |
| **F1 initial (fossil)** | 5185.17 | 0.41 | 841.87 | 0.29 | 1294.90 | 0.50 | 41.957 | 1.381 | 11.813 | 1.501 |
| **F2 initial (fossil)** | 2793.69 | 0.44 | 808.62 | 0.55 | 1154.93 | 0.98 | 32.393 | 0.710 | 13.890 | 1.424 |
| **F3 initial (fossil)** | 788.55 | 0.94 | 322.97 | 0.74 | 4908.43 | 0.70 | 26.395 | 0.687 | 10.226 | 0.686 |
| **pH 6 F1 final (fossil)** | 8705.756 | 0.678 | 3276.16 | 0.80 | 3302.639 | 0.806 | 39.788 | 0.678 | 10.008 | 1.700 |
| **pH 6 F2 final (fossil)** | 7994.756 | 0.271 | 2994.60 | 0.91 | 2590.900 | 0.929 | 42.096 | 0.549 | 7.719 | 0.902 |
| **pH 6 F3 final (fossil)** | 8778.912 | 0.862 | 2980.93 | 0.88 | 14783.962 | 1.354 | 35.631 | 0.523 | 9.541 | 0.723 |

| **Apatite and Calcite Mineral ICP-MS Data** | | | | | | | | | | |
| --- | --- | --- | --- | --- | --- | --- | --- | --- | --- | --- |
|  | **Na** | | **Mg** | | **Al** | | **K** | | **Mn** | |
|  | Conc.(ppm) | RSD | Conc.(ppm) | RSD | Conc.(ppm) | RSD | Conc.(ppm) | RSD | Conc.(ppm) | RSD |
| **Wards apatite** | 573.69 | 2.53 | 306.27 | 3.48 | 140.57 | 5.30 | <10.31 | N/A | 156.11 | 2.77 |
| **Wards calcite** | <11.45 | N/A | 438.60 | 2.24 | 11.33 | 20.36 | <9.28 | N/A | 261.34 | 1.33 |
| **Eisco apatite** | 97.74 | 2.92 | 32.58 | 0.87 | 37.27 | 11.32 | <11.00 | N/A | 404.59 | 0.99 |

| **Apatite and Calcite Mineral ICP-MS Data (Continued)** | | | | | | | | | | |
| --- | --- | --- | --- | --- | --- | --- | --- | --- | --- | --- |
|  | **Fe** | | **Sr** | | **Ba** | | **Ca** | | **P** | |
|  | Conc.(ppm) | RSD | Conc.(ppm) | RSD | Conc.(ppm) | RSD | Conc.(ppm) | RSD | Conc.(ppm) | RSD |
| **Wards apatite** | 576.40 | 2.63 | 1730.36 | 2.94 | 10.36 | 5.21 | 37.32 | 1.03 | 16.75 | 2.24 |
| **Wards calcite** | 16.82 | 1.63 | 140.63 | 1.70 | <0.38 | N/A | 37.76 | 0.43 | <0.02 | N/A |
| **Eisco apatite** | 164.37 | 0.95 | 728.43 | 1.22 | 10.14 | 4.11 | 37.03 | 0.65 | 16.78 | 0.46 |
