# Supplementary material for: Experimental dissolution of fossil bone under variable pH conditions
Source: PLoS One. 2022 Oct 13;17(10):e0274084. doi: 10.1371/journal.pone.0274084 (PMC9560490; doi:10.1371/journal.pone.0274084)
Supplement: S3 Appendix — (DOCX) [file pone.0274084.s003.docx]

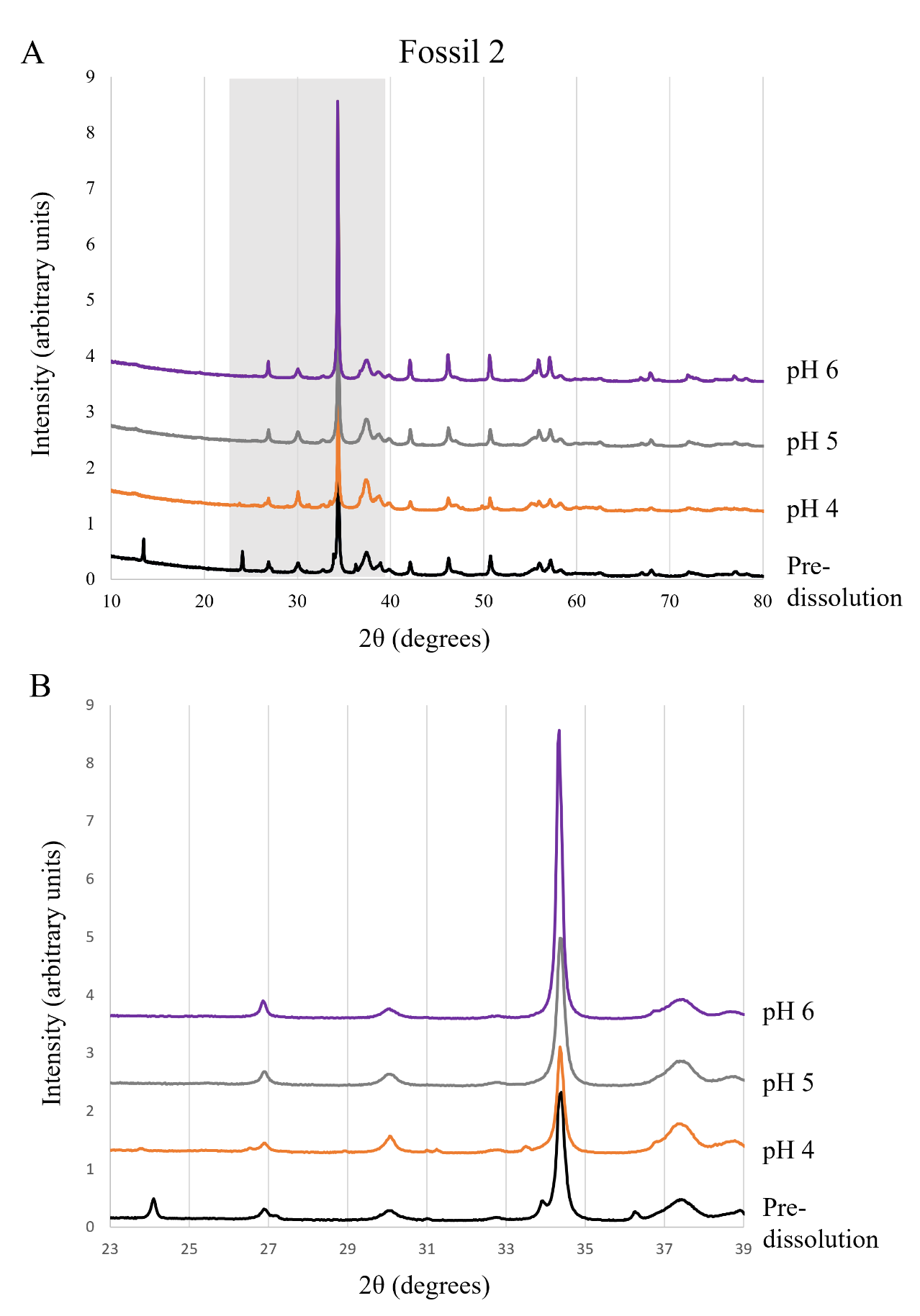


F2 stacked diffractogram patterns. B is the detailed view of the gray area in A (23-39 degrees).


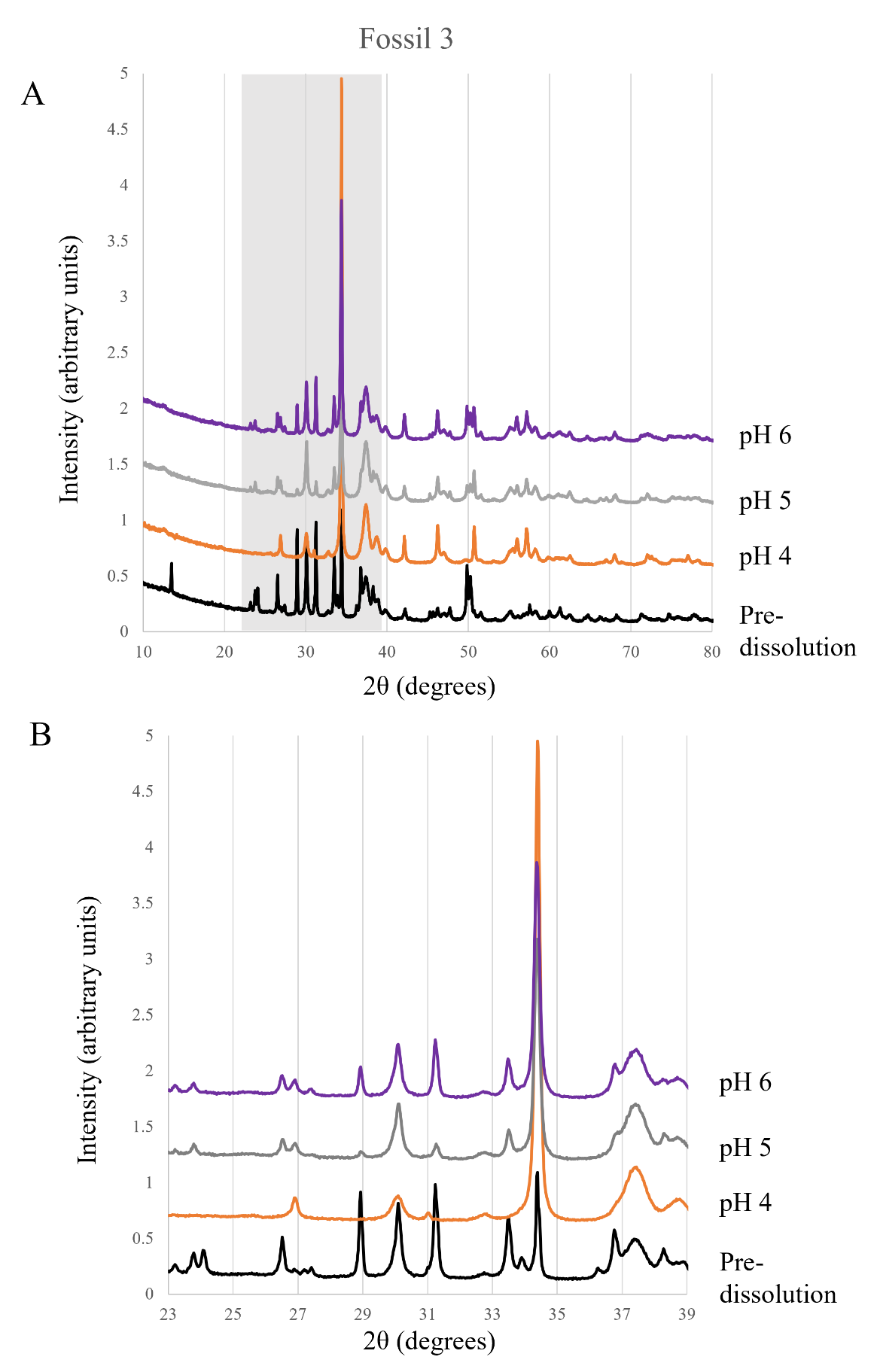


F3 stacked diffractogram patterns. B is the detailed view of the gray area in A (23-39 degrees).


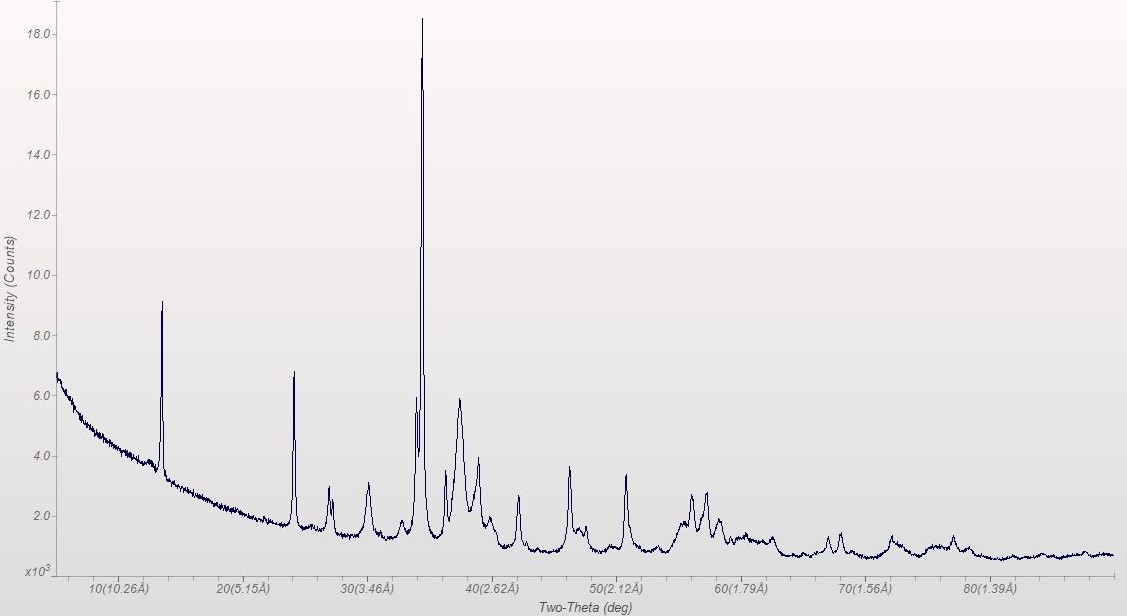


Diffractogram of pre-dissolution F1.


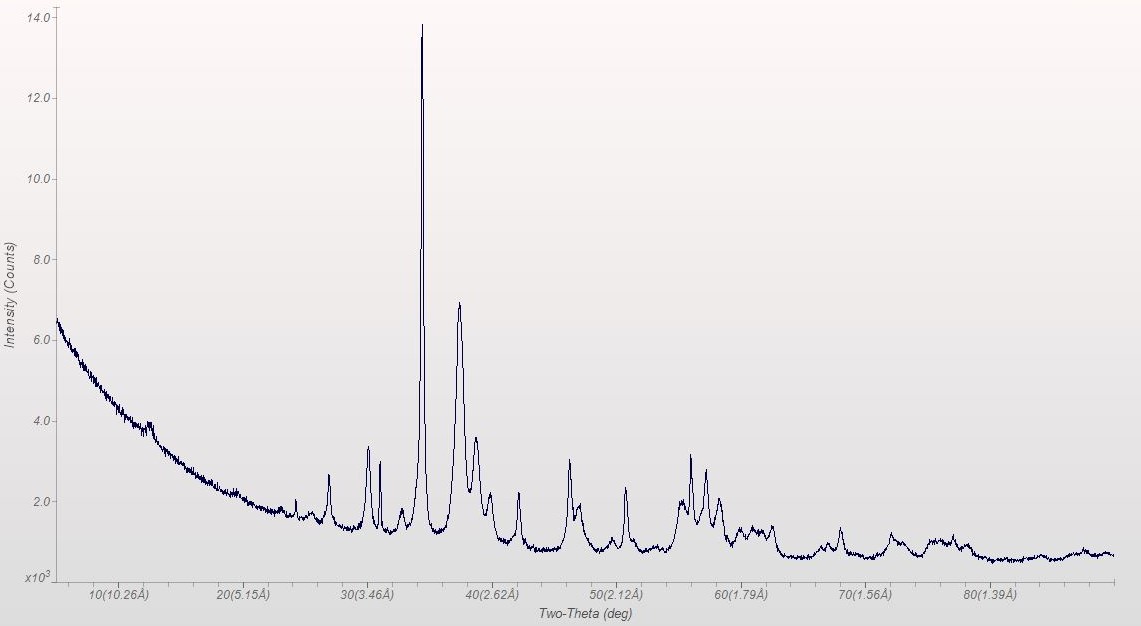


Diffractogram of F1 dissolved at pH 4.


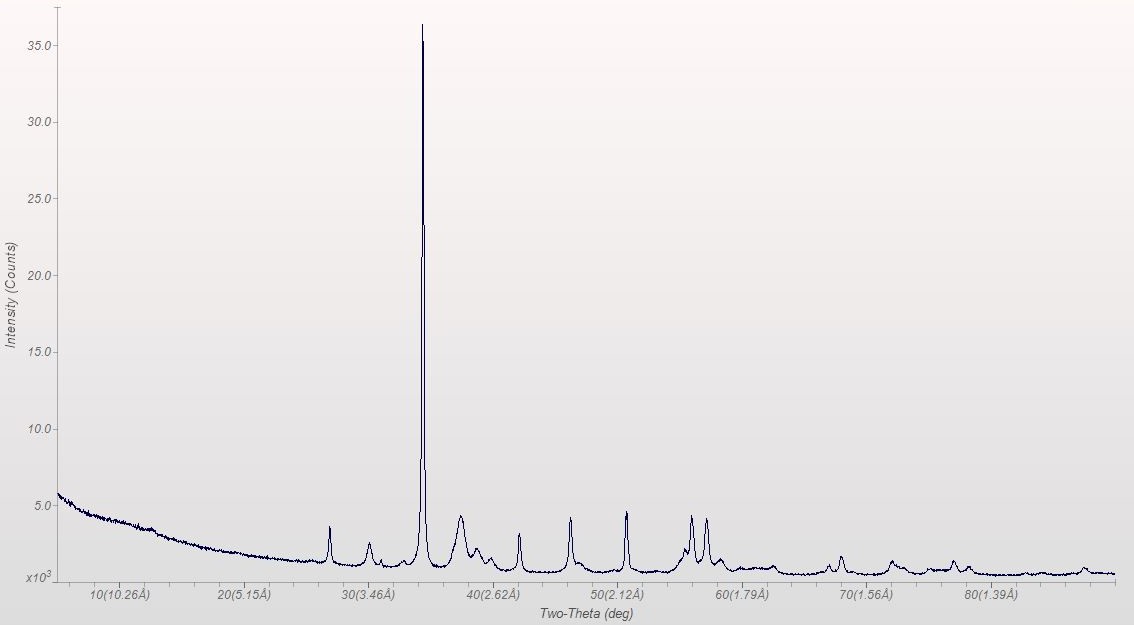


Diffractogram of F1 dissolved at pH 5.


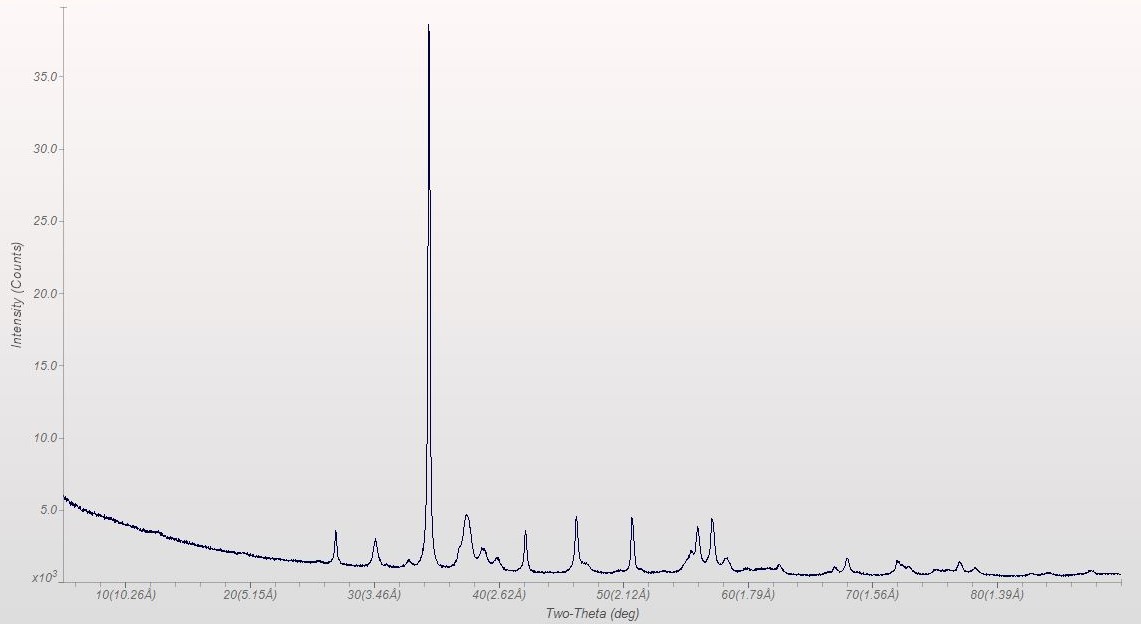


Diffractogram of F1 dissolved at pH 6.


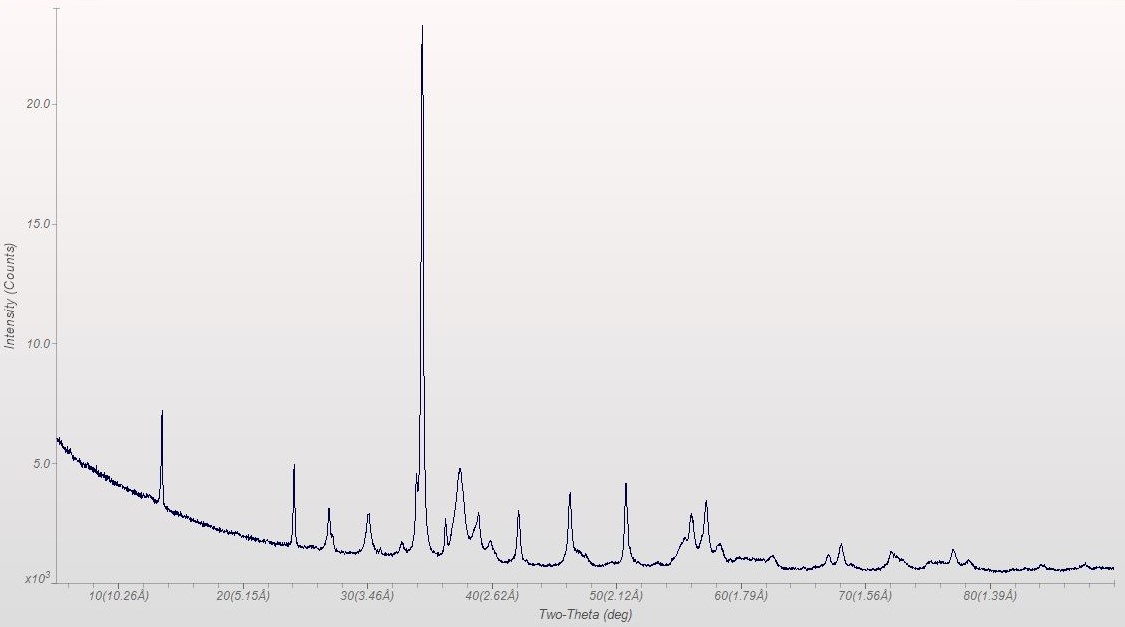


Diffractogram of pre-dissolution F2.


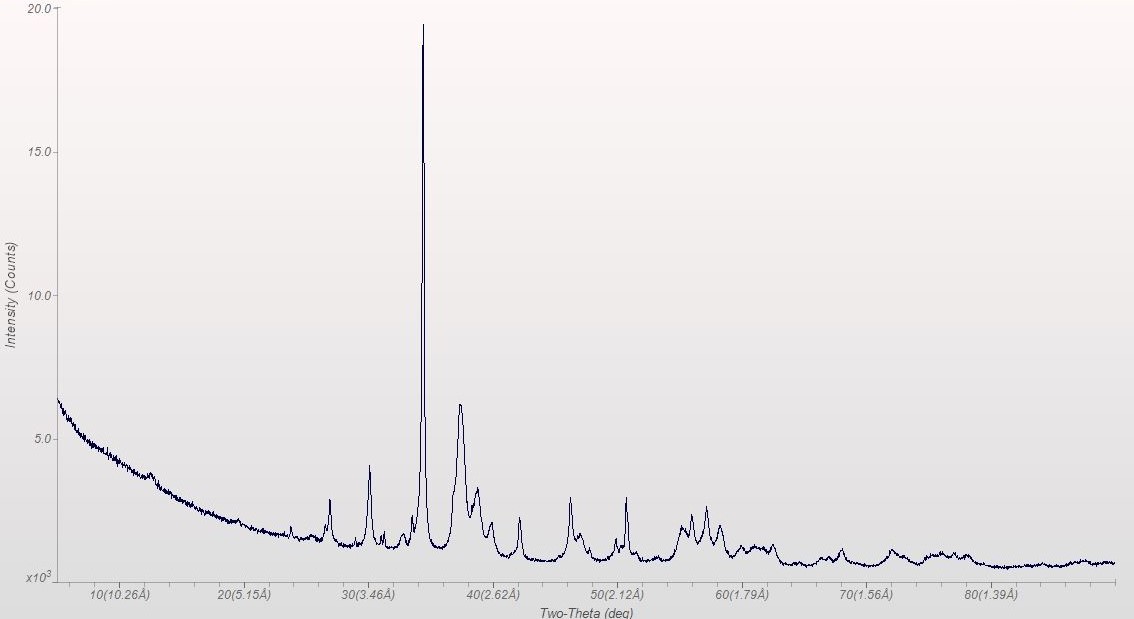


Diffractogram of F2 dissolved at pH 4.


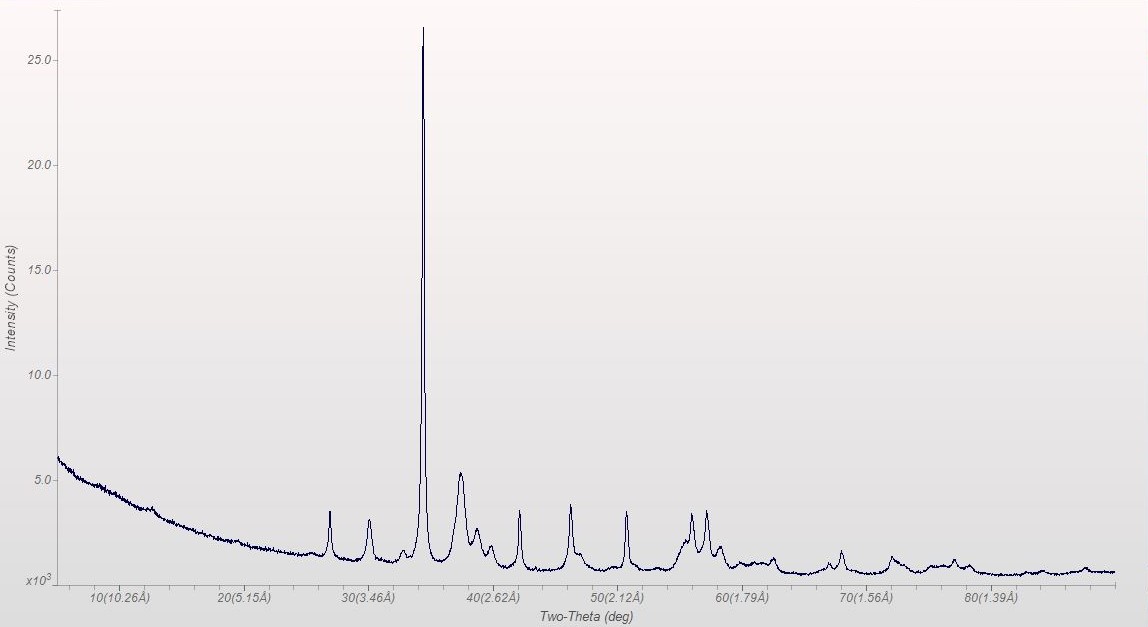


Diffractogram of F2 dissolved at pH 5.


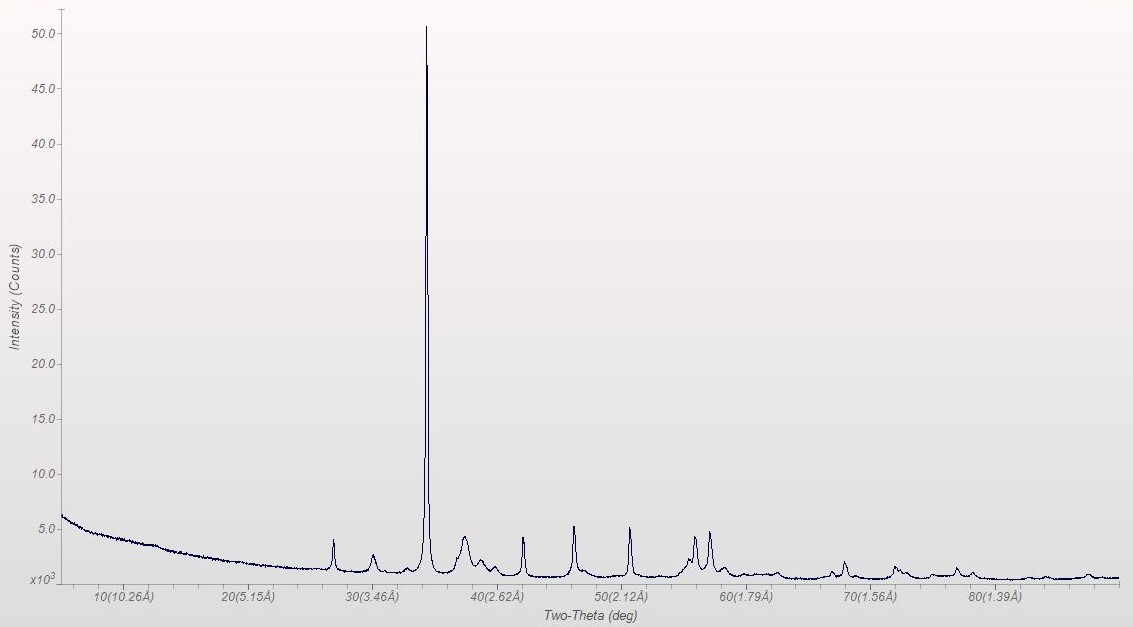


Diffractogram of F2 dissolved at pH 6.


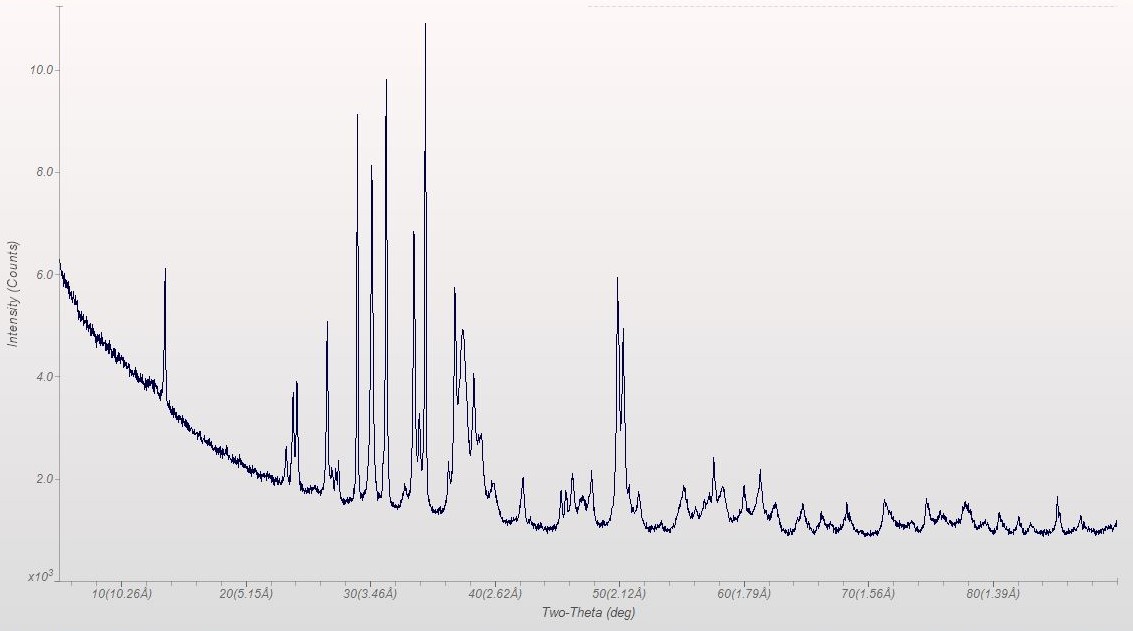


Diffractogram of pre-dissolution F3.


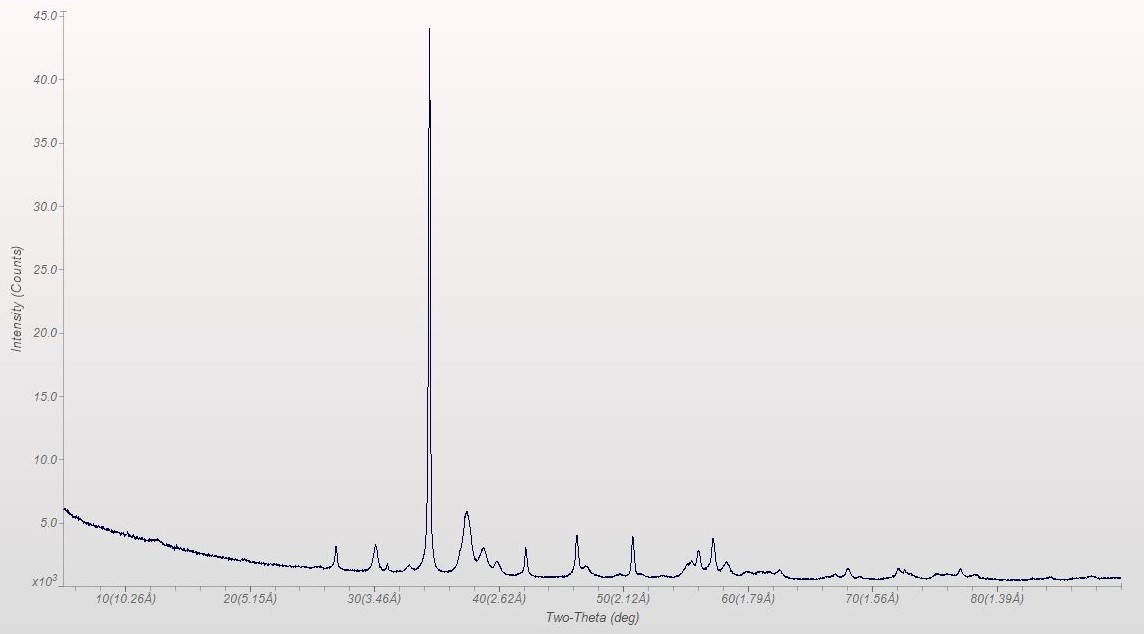


Diffractogram of F3 dissolved at pH 4.


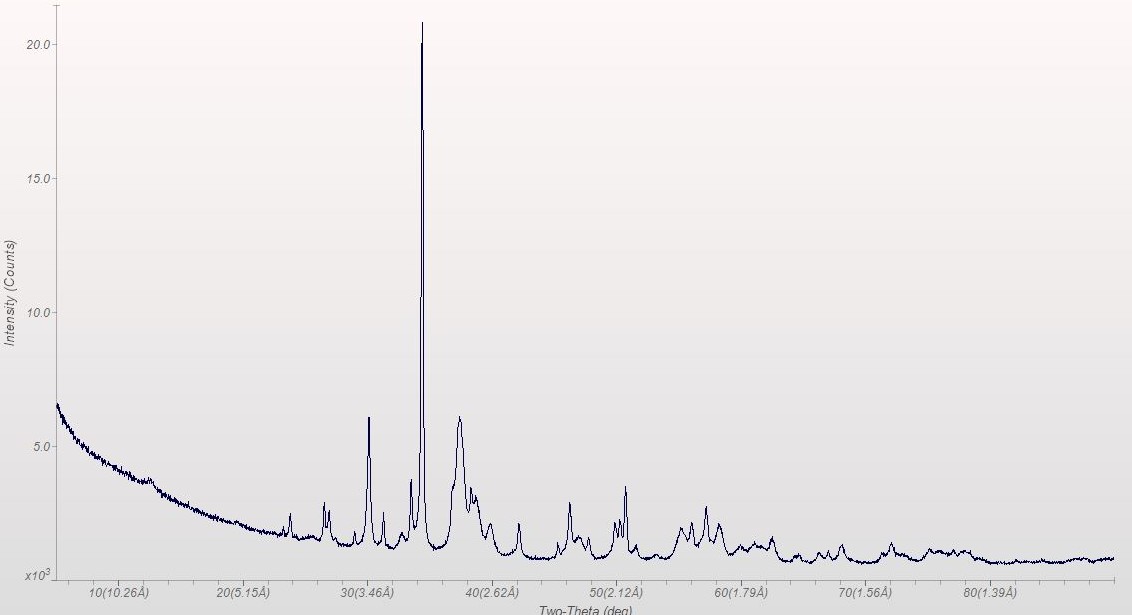


Diffractogram of F3 dissolved at pH 5.


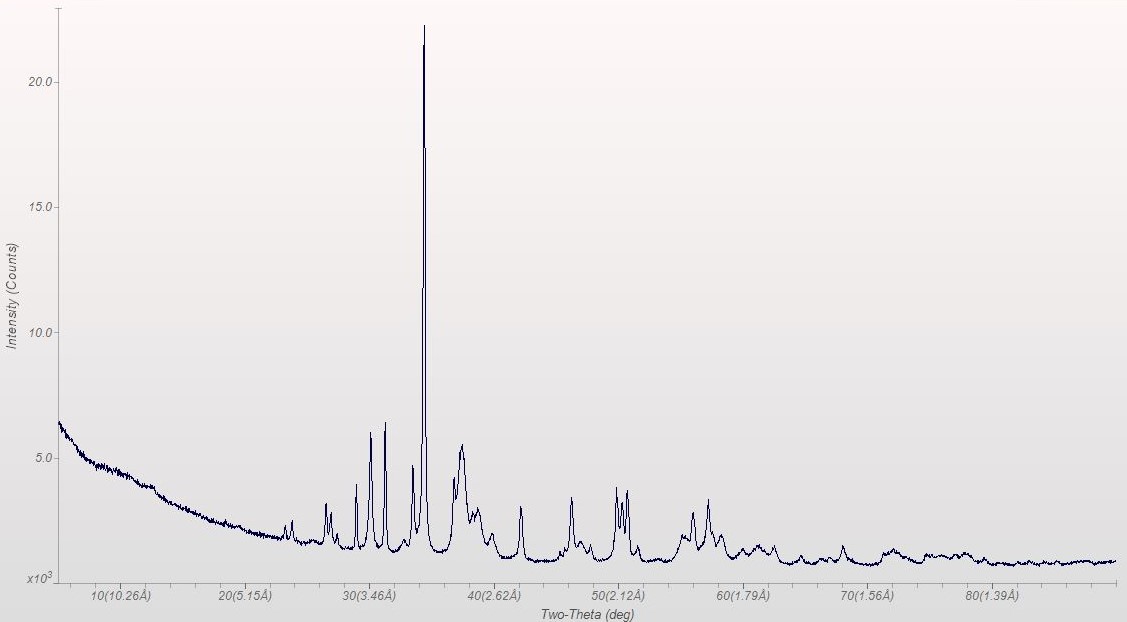


Diffractogram of F3 dissolved at pH 6.

| **F1 Mineralogical Composition** | | | | |
| --- | --- | --- | --- | --- |
| **Mineral** | **F1 initial (wt. %)** | **pH 4 (wt. %)** | **pH 5 (wt. %)** | **pH 6 (wt. %)** |
| Calcite | 39.68 | 35.66 | 56.5 | 62.7 |
| Gypsum | 11.9 | 0 | 0 | 0 |
| Carbonated fluorapatite | 47.24 | 36.57 | 0 | 31.8 |
| Hydroxylapatite | 0 | 27.43 | 43.1 | 4.1 |
| Fluorapatite | 0 | 0 | 0 | 1.5 |
| Quartz | 0 | 0 | 0.5 | 0 |
| Iron Sulfide | 1.18 | 0.34 | 0 | 0 |
| Barite | 0 | 0 | 0 | 0 |

| **F2 Mineralogical Composition** | | | | |
| --- | --- | --- | --- | --- |
| **Mineral** | **F2 initial (wt. %)** | **pH 4 (wt. %)** | **pH 5 (wt. %)** | **pH 6 (wt. %)** |
| Calcite | 44.7 | 42.3 | 54.4 | 56.7 |
| Gypsum | 6.5 | 0 | 0 | 0 |
| Carbonated fluorapatite | 0 | 57.7 | 38.3 | 0 |
| Hydroxylapatite | 48.8 | 0 | 5.3 | 20.5 |
| Fluorapatite | 0 | 0 | 0 | 22.7 |
| Quartz | 0 | 0 | 0 | 0.2 |
| Iron Sulfide | 0 | 0 | 2 | 0 |
| Barite | 0 | 0 | 0 | 0 |

| **F3 Mineralogical Composition** | | | | |
| --- | --- | --- | --- | --- |
| **Mineral** | **F3 initial (wt. %)** | **pH 4 (wt. %)** | **pH 5 (wt. %)** | **pH 6 (wt. %)** |
| Calcite | 16.04 | 19.9 | 29.3 | 42.2 |
| Gypsum | 8.18 | 0 | 0 | 0 |
| Carbonated fluorapatite | 20.28 | 15.2 | 46.4 | 27.5 |
| Hydroxylapatite | 39.96 | 17.2 | 6.7 | 0 |
| Fluorapatite | 0 | 47.1 | 8.9 | 22.8 |
| Quartz | 0 | 0.5 | 0 | 0 |
| Iron Sulfide | 0 | 0 | 4.9 | 0 |
| Barite | 15.54 | 0 | 3.8 | 7.5 |
