# Supplementary material for: Experimental dissolution of fossil bone under variable pH conditions
Source: PLoS One. 2022 Oct 13;17(10):e0274084. doi: 10.1371/journal.pone.0274084 (PMC9560490; doi:10.1371/journal.pone.0274084)
Supplement: S4 Appendix — (DOCX) [file pone.0274084.s004.docx]

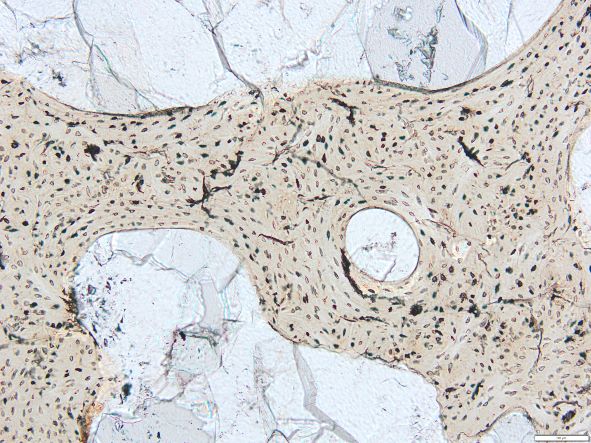


F1 pre-dissolution anterior end cap trabecular bone in plane polarized light. The scale bar in the bottom right is 100 μm.


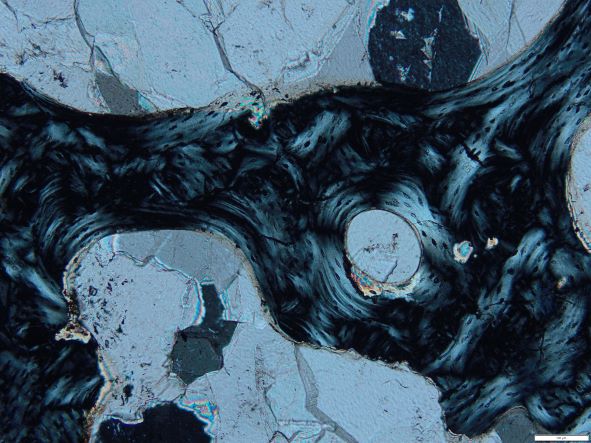


F1 pre-dissolution anterior end cap trabecular bone in cross-polarized light. The scale bar in the bottom right is 100 μm.


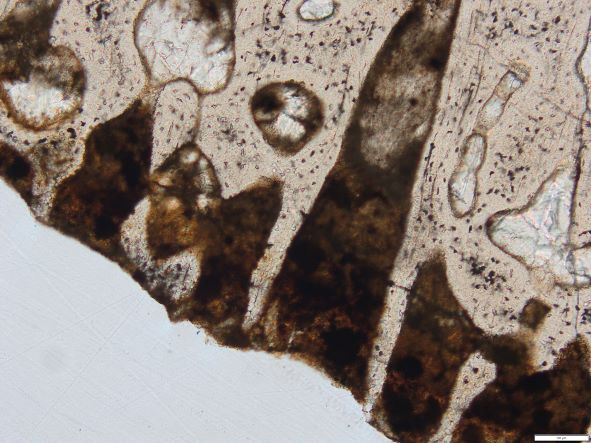


Edge of F1 pre-dissolution posterior end cap cortical bone in plane polarized light. The scale bar in the bottom right is 100 μm.


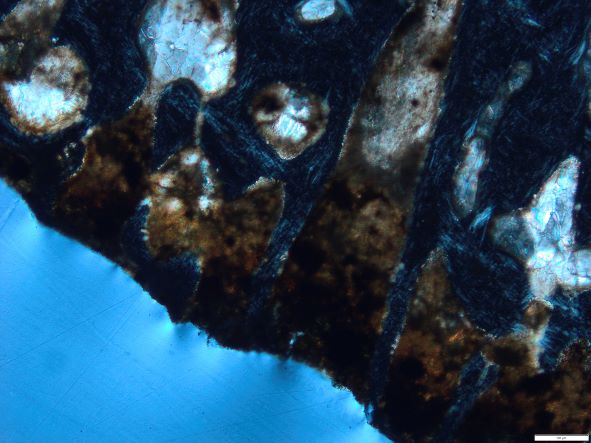


Edge of F1 pre-dissolution posterior end cap cortical bone in cross polarized light. The scale bar in the bottom right is 100 μm.


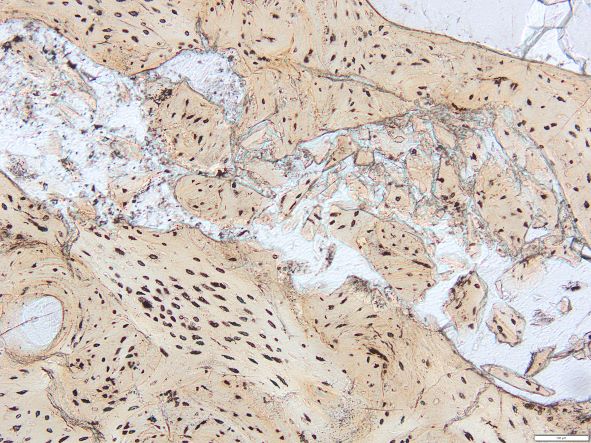


F1 trabecular bone dissolved at pH 4 in plane polarized light. The scale bar in the bottom right is 100 μm.


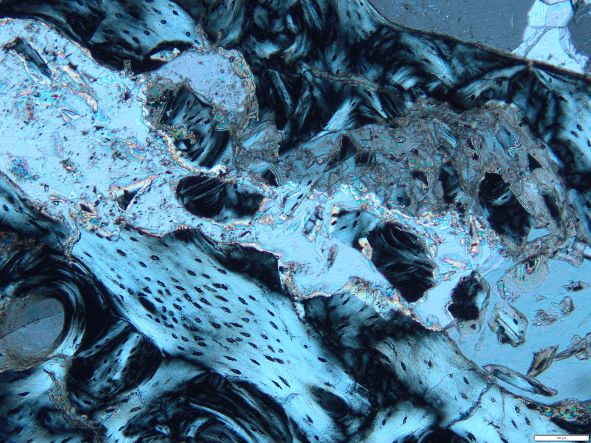


F1 trabecular bone dissolved at pH 4 in cross-polarized light. The scale bar in the bottom right is 100 μm.


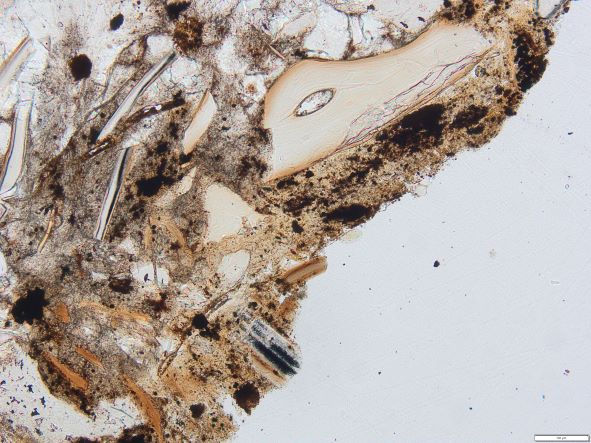


Edge of F1 dissolved at pH 4 in plane polarized light. The scale bar in the bottom right is 100 μm.


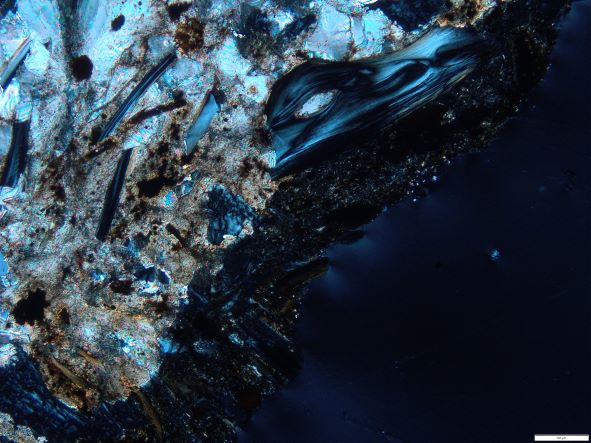


Edge of F1 dissolved at pH 4 in cross polarized light. The scale bar in the bottom right is 100 μm.


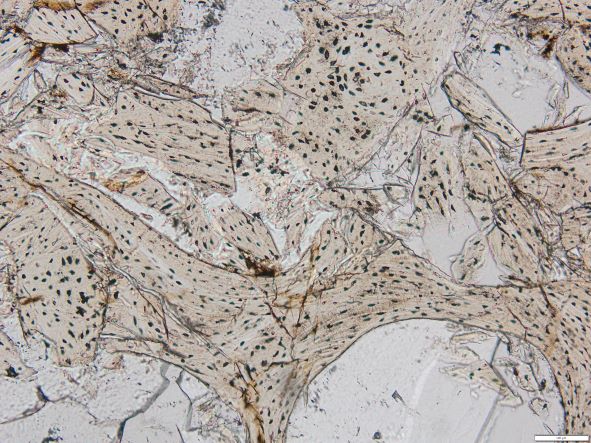


F1 trabecular bone dissolved at pH 5 in plane polarized light. The scale bar in the bottom right is 100 μm.


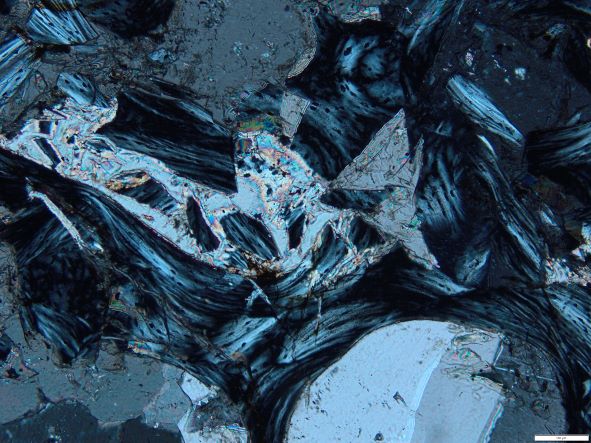


F1 trabecular bone dissolved at pH 5 in cross polarized light. The scale bar in the bottom right is 100 μm.


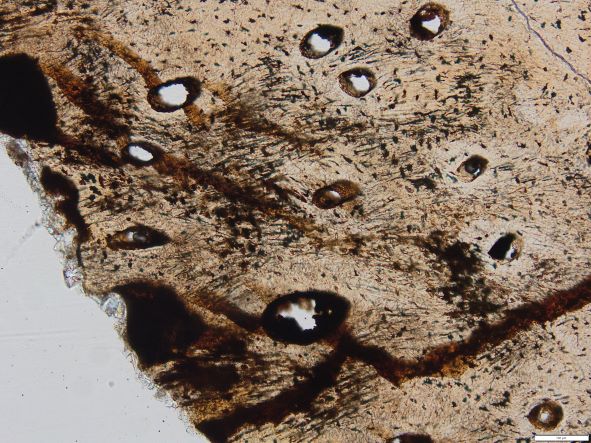


Edge of F1 cortical bone dissolved at pH 5 in plane polarized light. The scale bar in the bottom right is 100 μm.


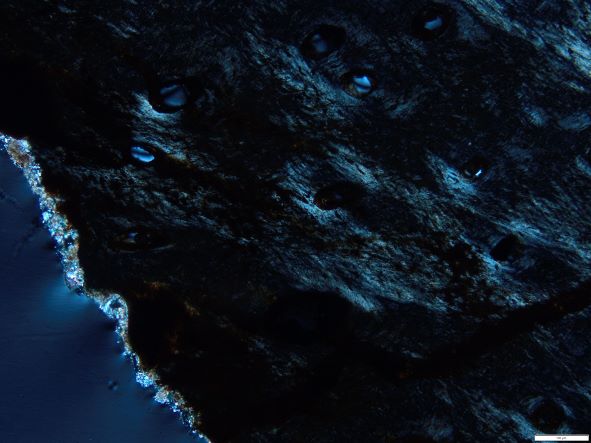


Edge of F1 cortical bone dissolved at pH 5 in cross polarized light. The scale bar in the bottom right is 100 μm.


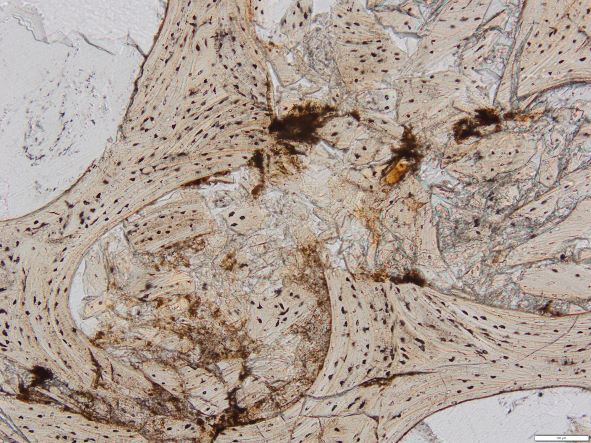


F1 trabecular bone dissolved at pH 6 in plane polarized light. The scale bar in the bottom right is 100 μm.


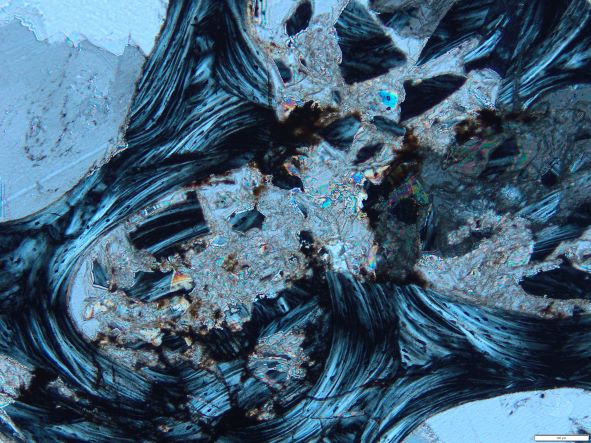


F1 trabecular bone dissolved at pH 6 in cross polarized light. The scale bar in the bottom right is 100 μm.


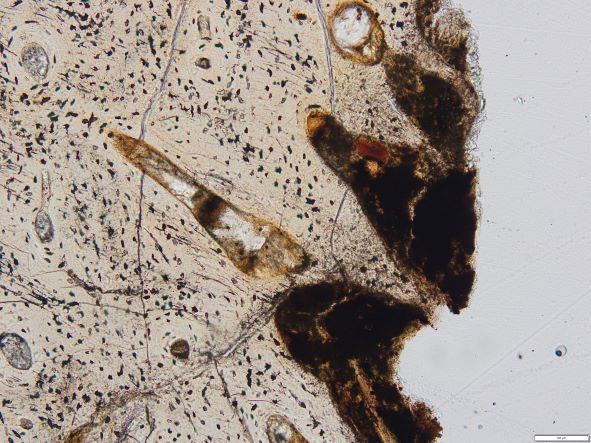


Edge of F1 cortical bone dissolved at pH 6 in plane polarized light. The scale bar in the bottom right is 100 μm.


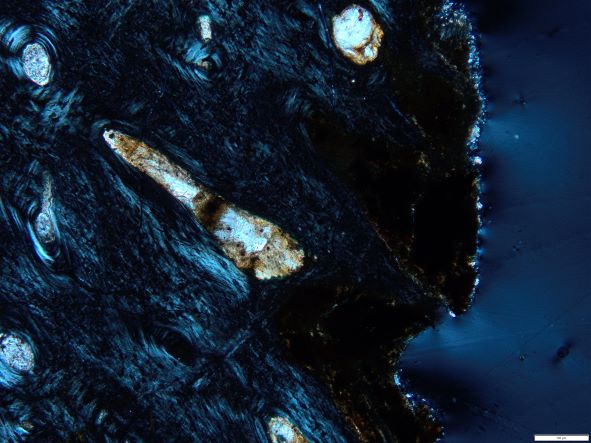


Edge of F1 cortical bone dissolved at pH 6 in cross polarized light. The scale bar in the bottom right is 100 μm.


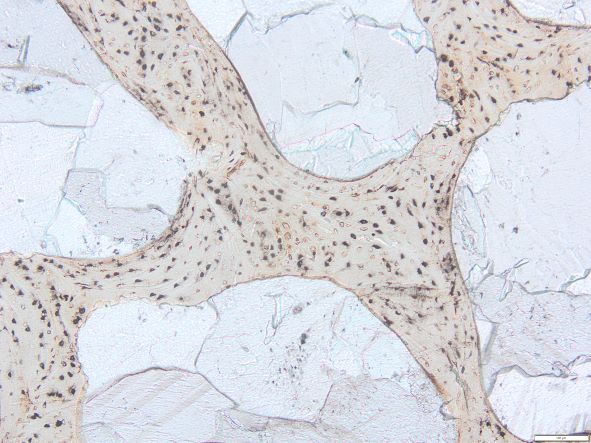


F2 pre-dissolution anterior end cap trabecular bone in plane polarized light. The scale bar in the bottom right is 100 μm.


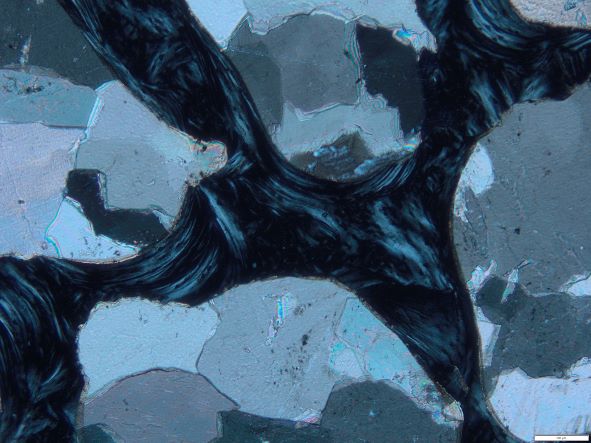


F2 pre-dissolution anterior end cap trabecular bone in cross polarized light. The scale bar in the bottom right is 100 μm.


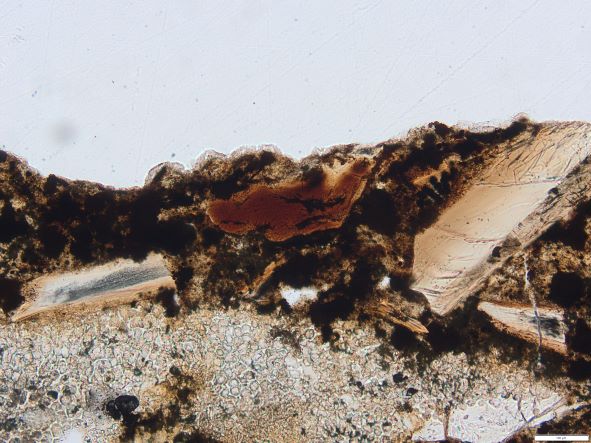


Edge of F2 pre-dissolution anterior end cap in plane polarized light. The scale bar in the bottom right is 100 μm.


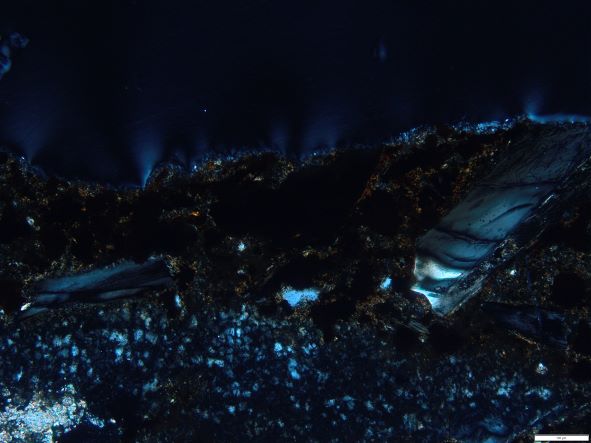


Edge of F2 pre-dissolution anterior end cap in cross polarized light. The scale bar in the bottom right is 100 μm.


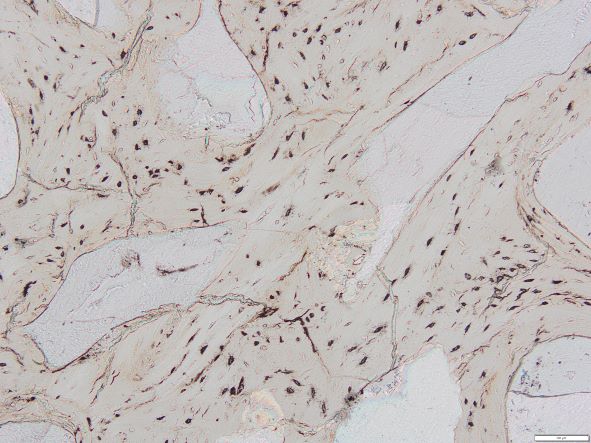


F2 pre-dissolution posterior end cap trabecular bone in plane polarized light. The scale bar in the bottom right is 100 μm.


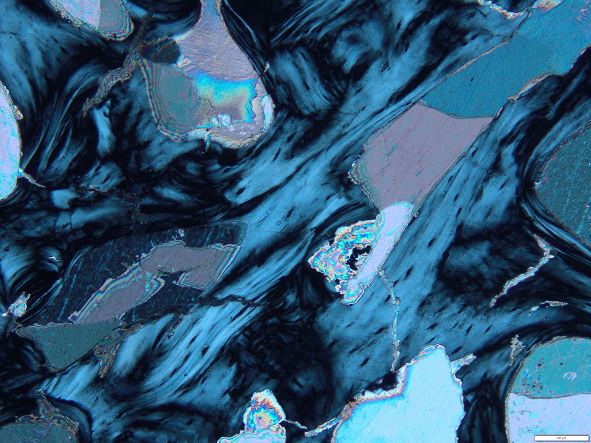


F2 pre-dissolution posterior end cap trabecular bone in cross polarized light. The scale bar in the bottom right is 100 μm.


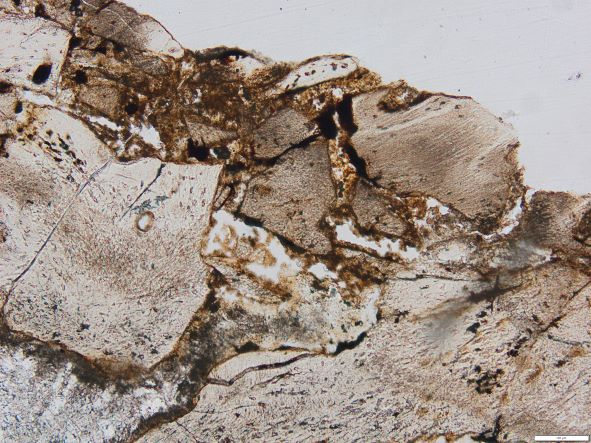


Edge of F2 pre-dissolution posterior end cap cortical bone in plane polarized light. The scale bar in the bottom right is 100 μm.


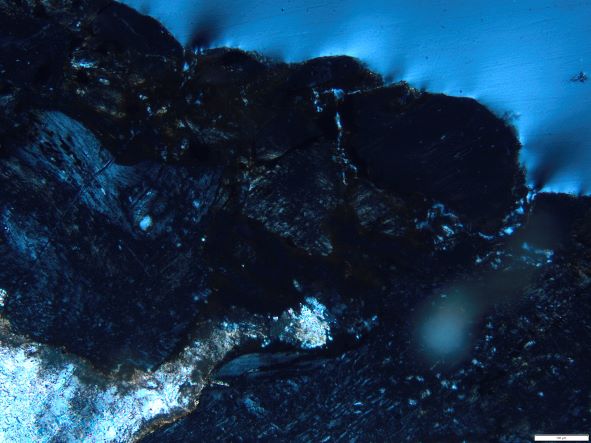


Edge of F2 pre-dissolution posterior end cap cortical bone in cross polarized light. The scale bar in the bottom right is 100 μm.


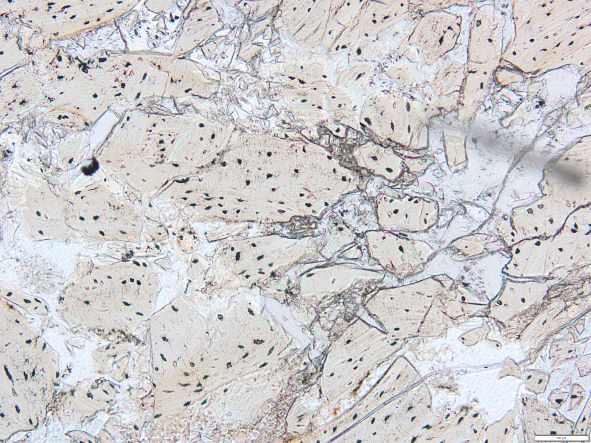


F2 trabecular bone dissolved at pH 4 in plane polarized light. The scale bar in the bottom right is 100 μm.


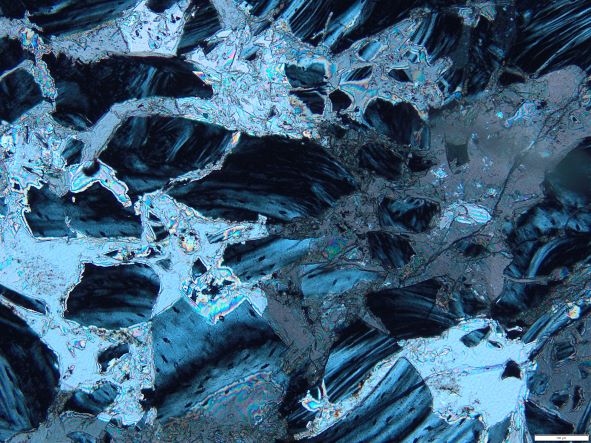


F2 trabecular bone dissolved at pH 4 in cross polarized light. The scale bar in the bottom right is 100 μm.


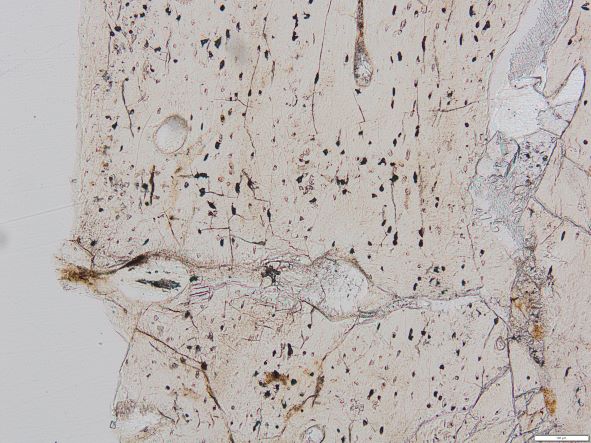


Edge of F2 cortical bone dissolved at pH 4 in plane polarized light. The scale bar in the bottom right is 100 μm.


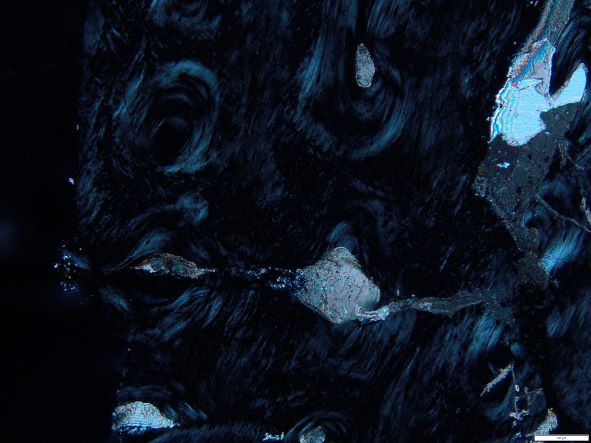


Edge of F2 cortical bone dissolved at pH 4 in cross polarized light. The scale bar in the bottom right is 100 μm.


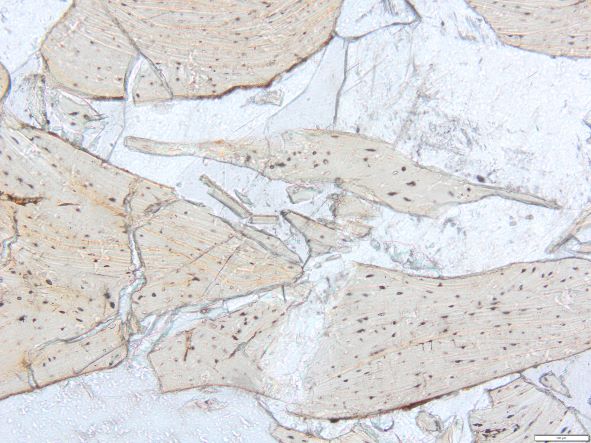


F2 trabecular bone dissolved at pH 5 in plane polarized light. The scale bar in the bottom right is 100 μm.


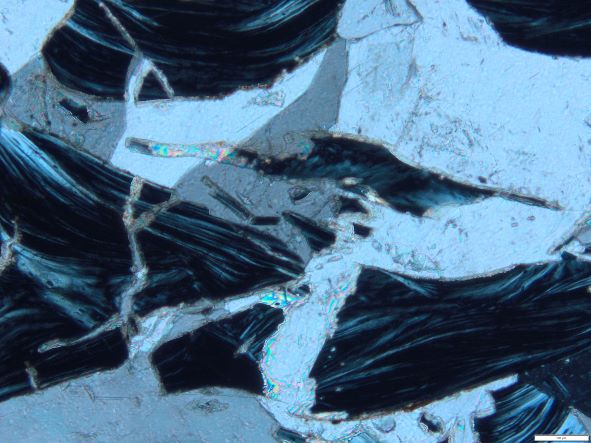


F2 trabecular bone dissolved at pH 5 in cross polarized light. The scale bar in the bottom right is 100 μm.


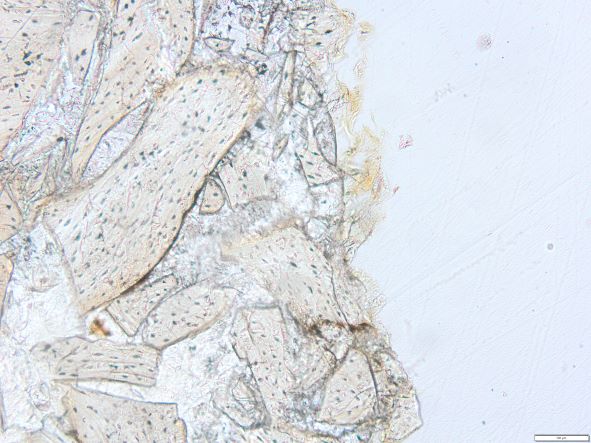


Edge of F2 dissolved at pH 5 in plane polarized light. The scale bar in the bottom right is 100 μm.


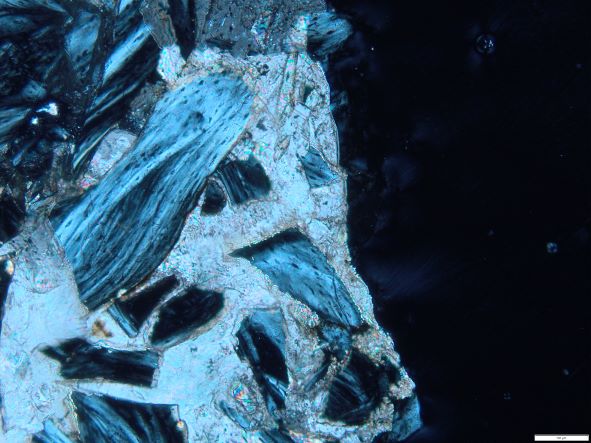


Edge of F2 dissolved at pH 5 in cross polarized light. The scale bar in the bottom right is 100 μm.


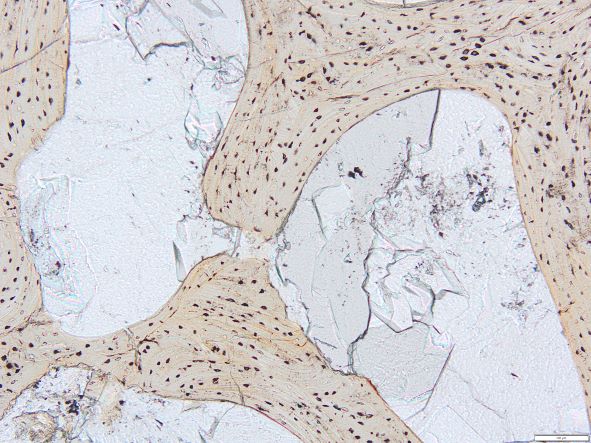


F2 trabecular bone dissolved at pH 6 in plane polarized light. The scale bar in the bottom right is 100 μm.


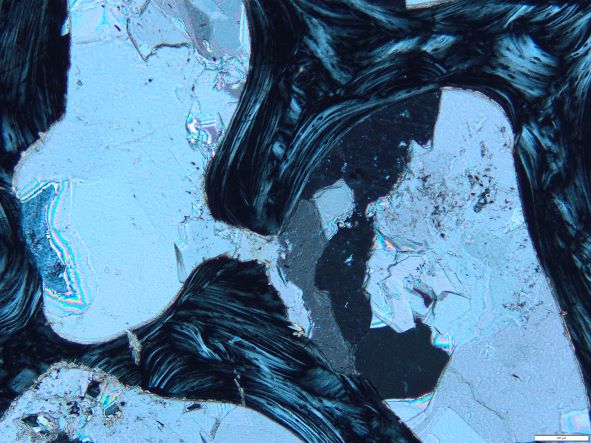


F2 trabecular bone dissolved at pH 6 in cross polarized light. The scale bar in the bottom right is 100 μm.


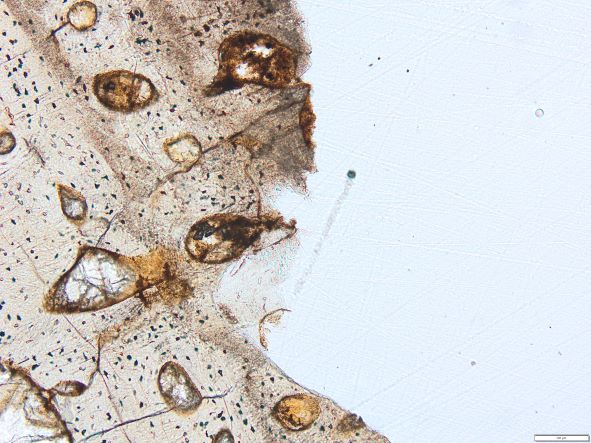


Edge of F2 cortical bone dissolved at pH 6 in plane polarized light. The scale bar in the bottom right is 100 μm.


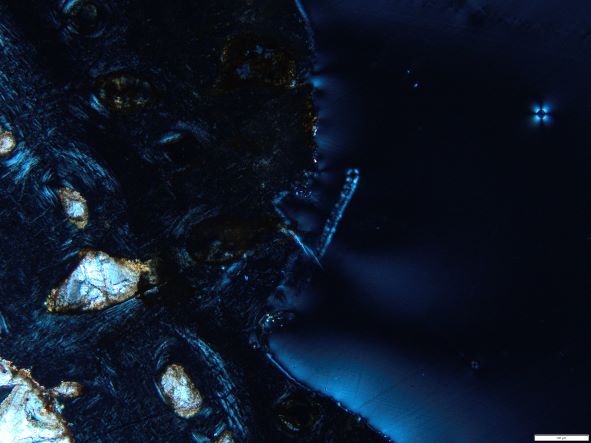


Edge of F2 cortical bone dissolved at pH 6 in cross polarized light. The scale bar in the bottom right is 100 μm.


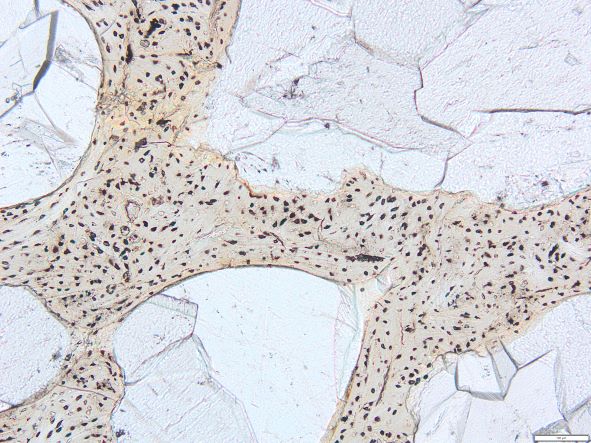


F3 pre-dissolution anterior end cap trabecular bone in plane polarized light. The scale bar in the bottom right is 100 μm.


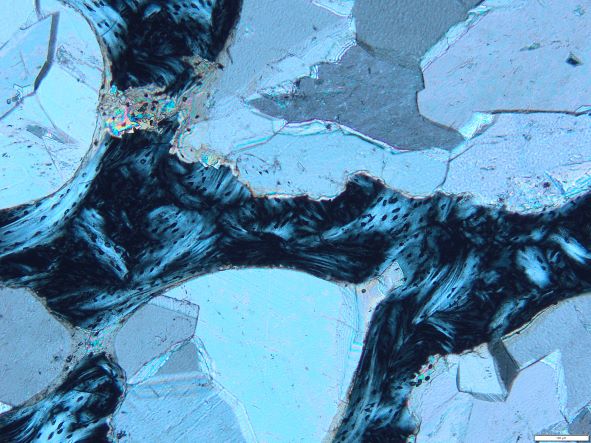


F3 pre-dissolution anterior end cap trabecular bone in cross polarized light. The scale bar in the bottom right is 100 μm.


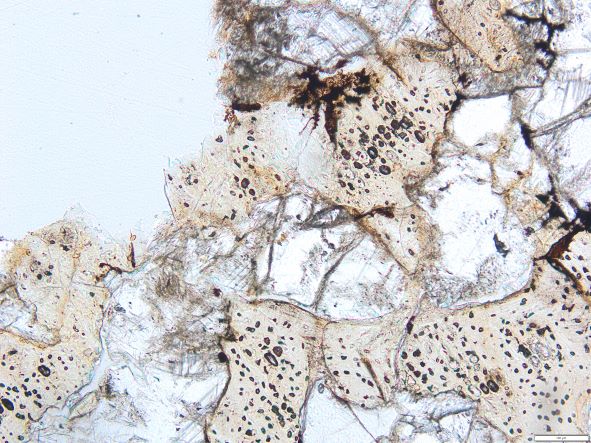


Edge of F3 pre-dissolution anterior end cap in plane polarized light. The scale bar in the bottom right is 100 μm.


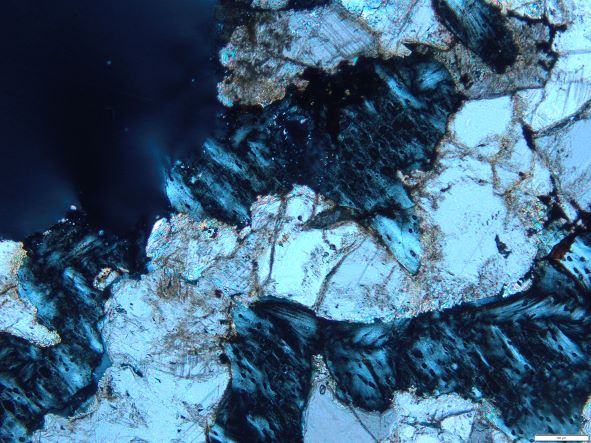


Edge of F3 pre-dissolution anterior end cap in cross polarized light. The scale bar in the bottom right is 100 μm.


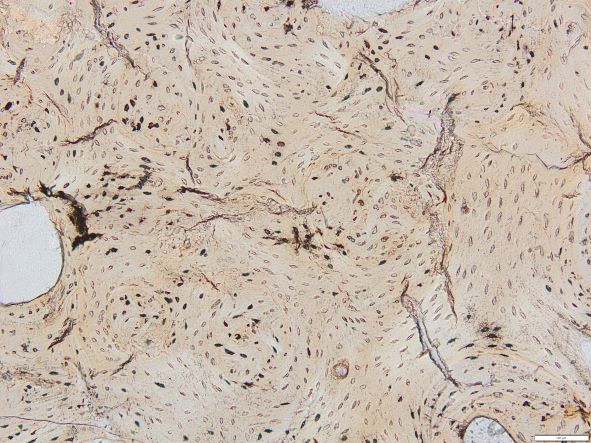


F3 pre-dissolution posterior end cap cortical bone, adjacent to trabecular bone, in plane polarized light. The scale bar in the bottom right is 100 μm.


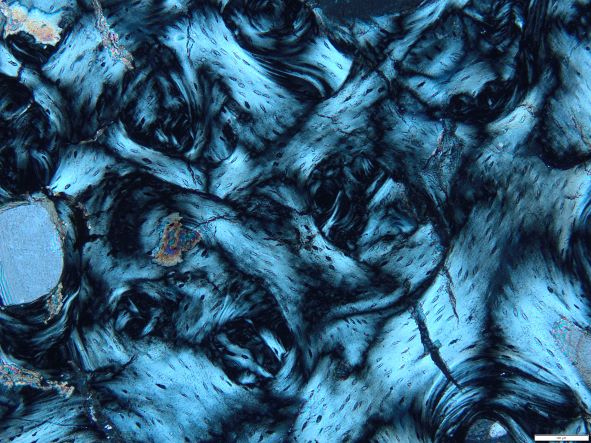


F3 pre-dissolution posterior end cortical bone, adjacent to trabecular bone, in cross polarized light. The scale bar in the bottom right is 100 μm.


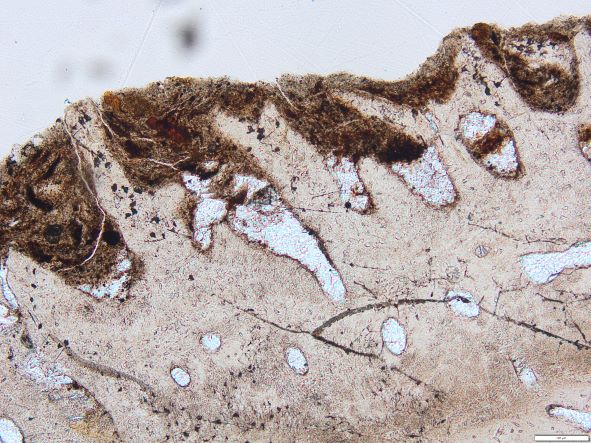


Edge of F3 pre-dissolution posterior end cap cortical bone in plane polarized light. The scale bar in the bottom right is 100 μm.


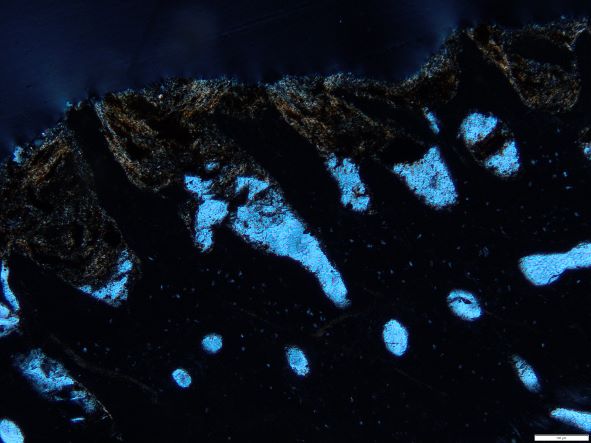


Edge of F3 pre-dissolution posterior end cap cortical bone in cross polarized light. The scale bar in the bottom right is 100 μm.


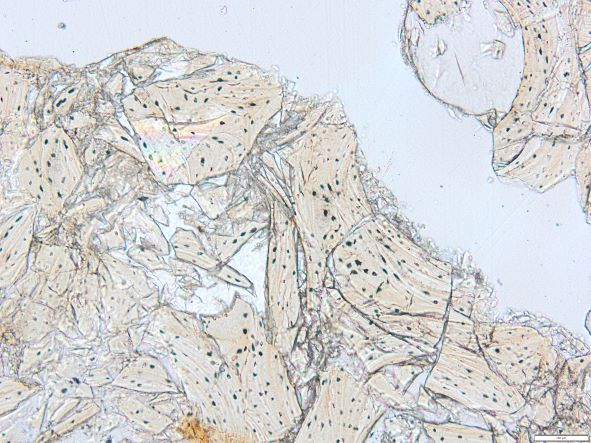


F3 trabecular bone dissolved at pH 4 in plane polarized light. The scale bar in the bottom right is 100 μm.


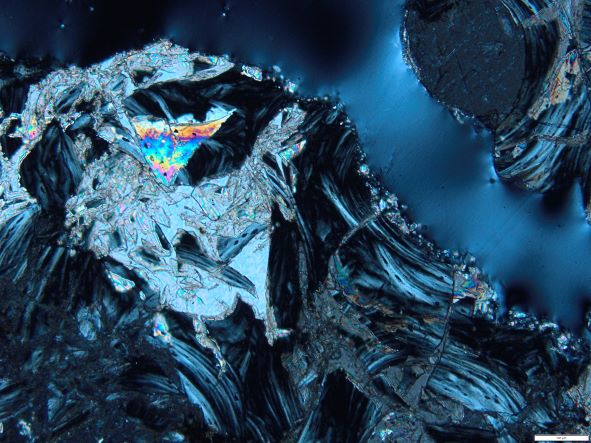


F3 trabecular bone dissolved at pH 4 in cross polarized light. The scale bar in the bottom right is 100 μm.


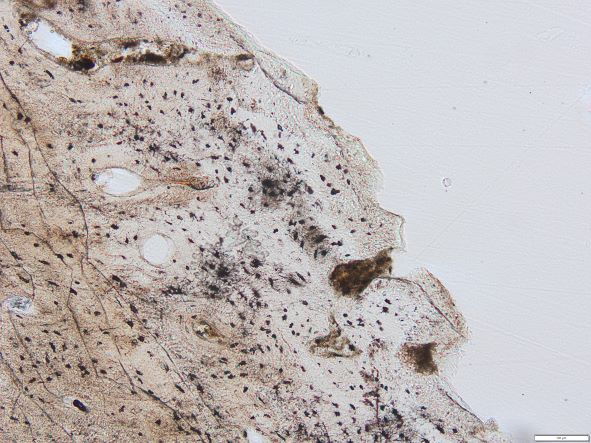


Edge of F3 cortical bone dissolved at pH4 in plane polarized light. The scale bar in the bottom right is 100 μm.


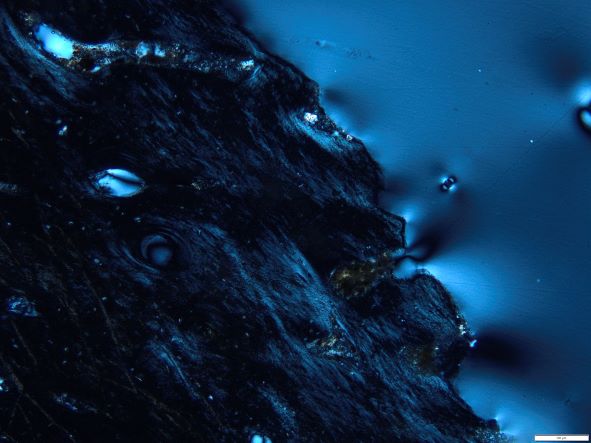


Edge of F3 cortical bone dissolved at pH 4 in cross polarized light. The scale bar in the bottom right is 100 μm.


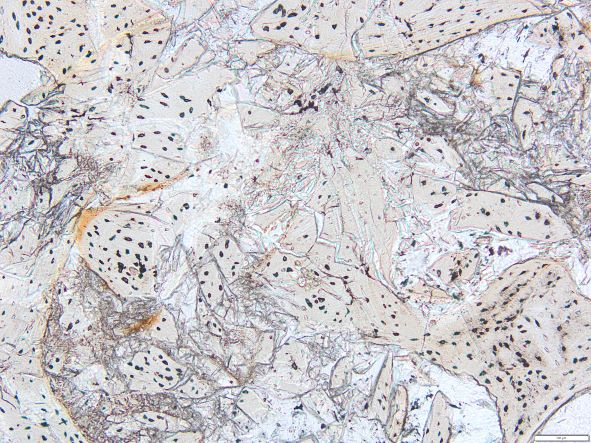


F3 trabecular bone dissolved at pH 5 in plane polarized light. The scale bar in the bottom right is 100 μm.


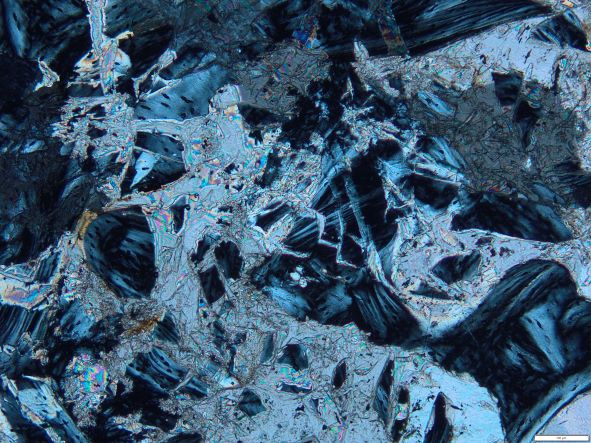


F3 trabecular bone dissolved at pH 5 in cross polarized light. The scale bar in the bottom right is 100 μm.


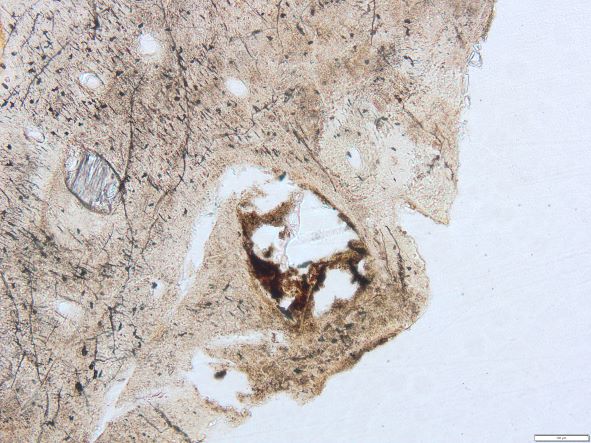


Edge of F3 cortical bone dissolved at pH 5 in plane polarized light. The scale bar in the bottom right is 100 μm.


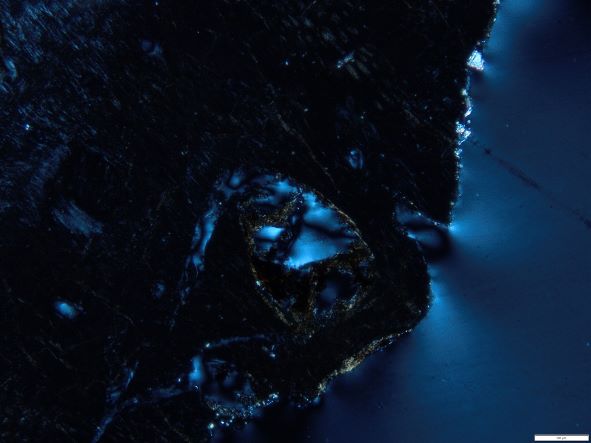


Edge of F3 cortical bone dissolved at pH 5 in cross polarized light. The scale bar in the bottom right is 100 μm.


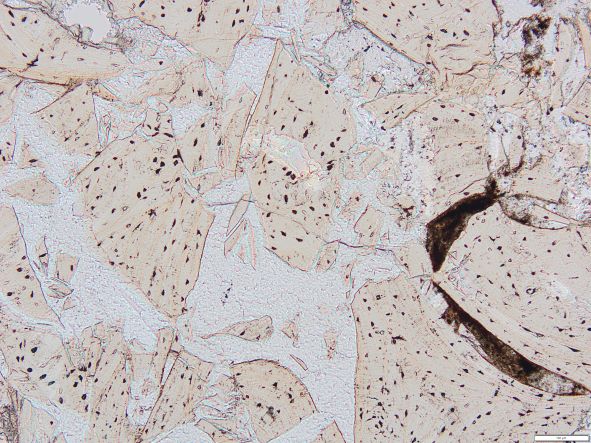


F3 trabecular bone dissolved at pH 6 in plane polarized light. The scale bar in the bottom right is 100 μm.


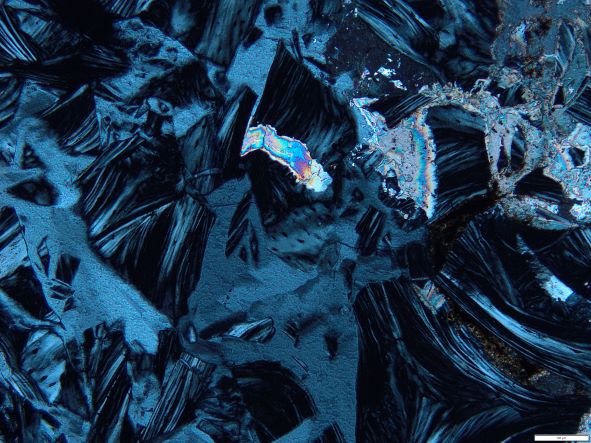


F3 trabecular bone dissolved at pH 6 in cross polarized light. The scale bar in the bottom right is 100 μm.


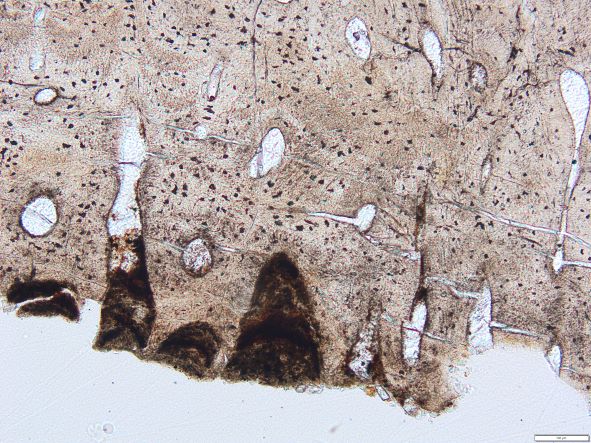


Edge of F3 cortical bone dissolved at pH 6 in plane polarized light. The scale bar in the bottom right is 100 μm.


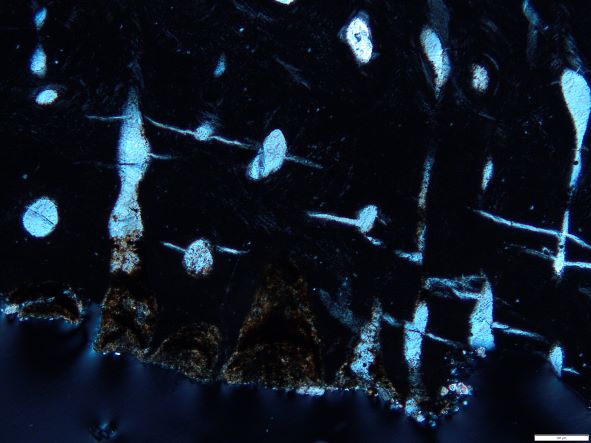


Edge of F3 cortical bone dissolved at pH 6 in cross polarized light. The scale bar in the bottom right is 100 μm.
