# Supplementary material for: Experimental dissolution of fossil bone under variable pH conditions
Source: PLoS One. 2022 Oct 13;17(10):e0274084. doi: 10.1371/journal.pone.0274084 (PMC9560490; doi:10.1371/journal.pone.0274084)
Supplement: S5 Appendix — (DOCX) [file pone.0274084.s005.docx]

#create object for pH4 mass loss for F1, F2, and F3

ph4<- c(0.8029, 0.6721, 0.4766)

#create object for pH 5 mass loss for F1, F2, and F3

ph5<- c(0.8353, 0.2893, 0.5216)

#create object for pH 6 mass loss for F1, F2, ad F3

ph6<- c(0.4592, 0.3387, 0.2846)

#paired t test for the pH4 and pH5 mass loss data

t.test(ph4, ph5, paired=TRUE)

#paired t test for the pH4 and pH6 mass loss data

t.test(ph4, ph6, paired=TRUE)

#paired t test for the pH5and pH6 mass loss data

t.test(ph5, ph6, paired=TRUE)

#entering ICP-MS data for t tests

#P water data

p4w<- c(10.86, 14.36, 18.74)

p5w<- c(6.62, 7.22, 6.69)

p6w<- c(0.00, 0.03, 0.00)

#t test for P water data

t.test (p4w, p5w, paired=TRUE)

t.test (p5w, p6w, paired=TRUE)

t.test (p4w, p6w, paired=TRUE)

#Ca water data

ca4w<- c(314.11, 251.38, 197.15)

ca5w<- c(329.77, 109.74, 202.65)

ca6w<- c(273.36, 185.48, 174.07)

#t test for calcium water data

t.test(ca4w, ca5w, paired=TRUE)

t.test(ca5w, ca6w, paired=TRUE)

t.test(ca4w, ca6w, paired=TRUE)

#P solid data

p4s<- c(-0.89, -6.07, 3.25)

p5s<- c(-3.15, -1.59, 0.73)

p6s<- c(-1.81, -6.17, -0.69)

#t test for P solid data

t.test (p4s, p5s, paired=TRUE)

t.test (p5s, p6s, paired=TRUE)

t.test (p4s, p6s, paired=TRUE)

#Ca solid data

ca4s<- c(-9.29, -9.83, 12.40)

ca5s<- c(-3.57, 3.44, 12.72)

ca6s<- c(-2.17, 9.70, 9.24)

#t test for calcium solid data

t.test(ca4s, ca5s, paired=TRUE)

t.test(ca5s, ca6s, paired=TRUE)

t.test(ca4s, ca6s, paired=TRUE)
